# Evidence-based surgical procedures to optimize caesarean outcomes: an overview of systematic reviews

**DOI:** 10.1016/j.eclinm.2024.102632

**Published:** 2024-05-19

**Authors:** Celina Gialdini, Monica Chamillard, Virginia Diaz, Julia Pasquale, Shakila Thangaratinam, Edgardo Abalos, Maria Regina Torloni, Ana Pilar Betran

**Affiliations:** a Centro Rosarino de Estudios Perinatales (CREP), Rosario, Argentina; b Facultat de Ciències de la Salut Blanquerna, Universitat Ramon Llull, Barcelona, Spain; c Institute of Metabolism and Systems Research, University of Birmingham, Birmingham, UK; d Centro de Estudios de Estado y Sociedad (CEDES), Buenos Aires, Argentina; e Evidence Based Healthcare Post-Graduate Program, Department of Medicine, São Paulo Federal University, São Paulo, Brazil; f UNDP/UNFPA/UNICEF/WHO/World Bank Special Programme of Research, Development and Research Training in Human Reproduction, Department of Sexual and Reproductive Health and Research, World Health Organization, Geneva, Switzerland

**Keywords:** Caesarean section, Surgery, Public health, Systematic review, Maternal health

## Abstract

**Background:**

Caesarean section (CS) is the most performed major surgery worldwide. Surgical techniques used for CS vary widely and there is no internationally accepted standardization. We conducted an overview of systematic reviews (SR) of randomized controlled trials (RCT) to summarize the evidence on surgical techniques or procedures related to CS.

**Methods:**

Searches were conducted from database inception to 31 January 2024 in Cochrane Database of Systematic Reviews, PubMed, EMBASE, Lilacs and CINAHL without date or language restrictions. AMSTAR 2 and GRADE were used to assess the methodological quality of the SRs and the certainty of evidence at outcome level, respectively. We classified each procedure-outcome pair into one of eight categories according to effect estimates and certainty of evidence. The overview was registered at PROSPERO (CRD 42023208306).

**Findings:**

The analysis included 38 SRs (16 Cochrane and 22 non-Cochrane) published between 2004–2024 involving 628 RCT with a total of 190,349 participants. Most reviews were of low or critically low quality (AMSTAR 2). The SRs presented 345 procedure-outcome comparisons (237 procedure versus procedure, 108 procedure versus no treatment/placebo). There was insufficient or inconclusive evidence for 256 comparisons, clear evidence of benefit for 40, possible benefit for 17, no difference of effect for 13, clear evidence of harm for 14, and possible harm for 5. We found no SRs for 7 pre-defined procedures. Skin cleansing with chlorhexidine, Joel-Cohen-based abdominal incision, uterine incision with blunt dissection and cephalad-caudal expansion, cord traction for placental extraction, manual cervical dilatation in pre-labour CS, changing gloves, chromic catgut suture for uterine closure, non-closure of the peritoneum, closure of subcutaneous tissue, and negative pressure wound therapy are procedures associated with benefits for relevant outcomes.

**Interpretation:**

Current evidence suggests that several CS surgical procedures improve outcomes but also reveals a lack of or inconclusive evidence for many commonly used procedures. There is an urgent need for evidence-based guidelines standardizing techniques for CS, and trials to fill existing knowledge gaps.

**Funding:**

UNDP-UNFPA-UNICEF-WHO-World Bank Special Programme of Research, Development and Research Training in Human Reproduction (HRP), a cosponsored programme executed by the 10.13039/100004423World Health Organization (WHO).

**Research in context**

**Evidence before this study**

Caesarean section has become one of the most performed surgeries worldwide. Over the last decades, its safety has significantly improved due to advances in surgical and anaesthetic techniques. Numerous systematic reviews provide evidence on the multiple procedures involved in a caesarean section. However, there is no previous overview of systematic reviews nor internationally accepted standardization of this operation. This situation hinders understanding the short and long-term maternal and neonatal effects of individual surgical procedures, and their interactions.

**Added value of this study**

This is the first overview of systematic reviews on surgical procedures used during a caesarean section. Our units of reporting were individual procedure-outcome pairs (e.g., changing gloves as a procedure and surgical site infection as an outcome). We classified each procedure-outcome pair in one of eight categories according to the effectiveness of the procedure for the specific outcome and the quality of the evidence. This approach provides a useful and more objective understanding of the value of each procedure to inform decision-making in clinical practice.

**Implications of all the available evidence**

Implementing evidence-based procedures for caesarean sections can improve safety maternal and perinatal outcomes, and optimise the use of available resources. This overview identified surgical procedures with clear evidence of benefit which should be universally adopted because of their value in optimizing outcomes, and several commonly used procedures with clear evidence of harm which should be avoided. The overview also identified numerous procedures currently used worldwide, with a lack of conclusive evidence from systematic reviews for several important outcomes. Bridging this gap for evidence and guidance requires future rigorous research, and the development of international recommendations for standardized evidence-based caesarean section.

## Introduction

A caesarean section (CS) can be a life-saving procedure when certain complications arise during pregnancy or childbirth and it is currently the most common major surgery in the world.^1^ In the last three decades, the use of CS has increased to unprecedented levels, from a global average of about 6% in 1990 to 21% in 2018, reaching over 50% in several middle-income countries.^2^ In an increasing number of countries, what was once a rare operation has become the most common mode of giving birth.^2^

Over the last decades, the safety of CS has significantly improved due to advances in surgical and anaesthetic techniques, and new modifications continue to be tested.^3^, ^4^, ^5^, ^6^ In addition, many non-surgical procedures (e.g., home preoperative washing/shaving, fasting, prophylactic antibiotics) have been introduced immediately before and after a CS to further improve outcomes and recovery.^7^, ^8^, ^9^, ^10^, ^11^, ^12^

Yet, some surgical and non-surgical procedures involved in a CS are of uncertain or unknown value because either their effectiveness has not been sufficiently and rigorously evaluated in randomized controlled trials (RCT) or the results of individual trials have not been pooled in meta-analyses.^4^^,^^5^ Currently, there is no internationally accepted standardization of all the procedures involved in conducting intra-partum or pre-labour CS. The use of surgical and medical procedures for CS varies widely not only in different settings but also between surgeons working in the same facility.^12^

Despite continuous scientific and medical developments, a CS is not without risks.^13^ As with any surgery, a CS is associated with short-term risks for the mother (e.g., infection, haemorrhage, organ injury, anaesthetic complications) and the baby (e.g., iatrogenic prematurity, breastfeeding and respiratory difficulties) as well as long-term risks (e.g., chronic maternal pain, childhood asthma and obesity) and complications in subsequent pregnancies (e.g., uterine rupture, placenta praevia/accreta).^14^^,^^15^ These risks are higher in women with limited access to comprehensive obstetric care, and particularly in low- and middle-income countries (LMIC) where women are most vulnerable to unsafe procedures.^16^ Projections show that by 2030, almost 30% of women worldwide will give birth by CS, which represents 38 million caesareans annually, of which 33.5 million will be in LMIC.^2^ Although individual level risks may be low, the risks associated with CS can have significant health and financial impacts at population and health-systems level. As CS use continues to grow, it is crucial to identify and implement evidence-based practices that optimize patient outcomes and minimize risks, thus reducing preventable morbidity and mortality and avoiding misuse of resources in limited and overburdened health systems.

There are no previous overviews that compiled the evidence from systematic reviews (SRs) on all procedures (surgical, medical, anaesthetic) involved in a CS. This gap led us to perform a series of overviews to summarize the most up-to-date evidence-based procedures related to CS. We also aimed to identify evidence gaps to guide future research. This manuscript summarizes the findings of SRs of randomized trials on surgical procedures related to CS. The overviews of SRs on medical and anaesthetic procedures related to CS are reported elsewhere.

## Methods

We conducted this overview according to the recommendations proposed by the Cochrane Handbook for Systematic Reviews of Interventions.^17^ and present it according to the PRIOR reporting guideline.^18^

The protocol for this overview was registered at Prospero (CRD 42023208306, https://www.crd.york.ac.uk/prospero/display_record.php?RecordID=208306).

### Types of studies

We included all published SRs of RCTs that examined the effectiveness and/or safety of patient-focused surgical procedures related to CS in humans. We excluded SRs of studies with other designs (e.g., cohorts, case-controls or before-and-after), and SR protocols.

### Type of participants

We included SRs of studies involving women of any age, race, socioeconomic condition or parity, with a singleton or multiple pregnancy at any gestational age, with any foetal presentation, undergoing primary or repeat, elective or emergency CS in the first or second stage of a spontaneous or induced labour, under any type of anaesthesia. SRs that only included studies that assessed procedures in patients with specific health conditions (e.g., diabetes, obesity, HIV) were excluded.

### Type of interventions

We included SRs that assessed at least one of a pre-specified list of CS related surgical procedures conducted in the operating theatre by health care providers: skin cleansing; use of adhesive drape; abdominal incision (skin incision, subcutaneous incision, fascial incision, rectus incision/dissection, peritoneum incision); use of retractors; use of aspiration device throughout the surgery; use of surgical swabs; bladder flap; uterine incision and expansion; foetal extraction; timing of umbilical cord clamping; placental extraction; cervical dilatation; uterine cleansing; uterine exteriorization; uterine closure; abdominal irrigation; changing gloves/double gloving; abdominal closure (peritoneum closure, rectus closure, fascial closure, subcutaneous closure, skin closure); use of drains; wound dressing.

The list of procedures was developed based on the Coronis Trial,^19^ international guidelines,^7^, ^8^, ^9^, ^10^, ^11^, ^12^ clinical and research experience of the overview authors, and informal consultation with international health professionals working in the field. As comparators we utilized the alternative interventions used by the original systematic review authors. Depending on the review, this could refer to no treatment or another treatment or intervention.

### Type of outcomes

Although there are core outcome sets for specific aspects of a caesarean, there is no published core outcome set for CS.^20^^,^^21^ The pre-specified maternal and perinatal outcomes used in this overview were compiled based on the same sources described for the list of interventions.

The pre-specified maternal outcomes were: febrile morbidity (fever, wound infection, endometritis, thrombophlebitis, peritonitis, urinary tract infection, need for antibiotics other than prophylaxis, sepsis); haemorrhagic morbidity (postpartum haemorrhage, anaemia, blood transfusion, need for additional uterotonic other than prophylaxis); pain (wound pain, pelvic pain, dysuria, headache, need for additional analgesics); short-term recovery (length of hospital stay/prolonged hospital stay >7 postpartum days, ambulation, breastfeeding, ability to care of the baby without help, self-care without help, bonding, maternal depression, wound dehiscence); long-term complications (chronic pain, incisional hernia, intra-abdominal adhesions, sub-fertility, dyspareunia, future pregnancy complications); other (nausea, vomiting, operating time, readmission to hospital after discharge); satisfaction with care (women and providers); acceptability; severe morbidity (hysterectomy, visceral damage, intensive care unit admission, deep vein thrombosis, pulmonary embolism, shock, cardiac arrest, pulmonary oedema, central venous access, respiratory failure, need for cardiopulmonary reanimation, seizures, encephalopathy, non-anaesthetic intubation, need for additional surgical procedures or return to operating room, re-laparotomy, arterial ligation, B-Lynch, curettage, maternal near-miss); maternal death.

The pre-specified neonatal outcomes were: severe morbidity (respiratory distress syndrome, transient tachypnoea, low Apgar scores, infections/HIV, neonatal intensive care unit admission, neonatal trauma); long-term outcomes; stillbirth; neonatal death; perinatal death.

### Search methods for identification of reviews

We created a comprehensive search strategy (Annex 2) using appropriate key words (and synonyms) for CS and the list of interventions. We ran the search in five electronic databases (Cochrane Database of Systematic Reviews, PubMed, EMBASE, Lilacs and CINAHL) from database inception to 31 January 2024, without language or date restrictions. The citations were uploaded in Covidence (https://www.covidence.org/) and duplicates deleted. We complemented the search by screening the reference lists of international guidelines and overviews of systematic reviews.

### Process of review selection and data extraction

The titles and abstracts of all retrieved citations were screened independently by two reviewers working in pairs (MC, VD, CG, JP, APB) to select potentially relevant studies for full-text reading. Full-text evaluation was conducted independently by two reviewers working in pairs (MC, VD, CG, JP, APB) and the SRs that fulfilled the aforementioned selection criteria were included in the overview. Conflicts were resolved through discussion with a third overview author. Reasons for exclusion were recorded.

For each included SR, we extracted data pertaining to all pre-specified procedures and outcomes as reported in the original SR. One reviewer extracted data and a second reviewer checked for accuracy. Disagreements were resolved by discussion. The data was extracted into a data collection form specifically created for this overview.

### Methodological quality of systematic reviews

Two reviewers independently used the AMSTAR 2 tool^22^^,^^23^ to assess the overall quality of all included SRs. The following critical domains were assessed when reported using the online tool (https://amstar.ca/Amstar_Checklist.php):

1. Protocol registered before commencement of the review
2. Adequacy of the literature search
3. Justification for excluding individual studies
4. Risk of bias from individual studies being included in the review
5. Appropriateness of meta-analytical methods
6. Consideration of risk of bias when interpreting the results of the review
7. Assessment of presence and likely impact of publication bias

### Quality or certainty of evidence extracted from included reviews

For each procedure-outcome pair, we used the GRADE assessment provided in the SRs.^24^ If it was not provided, two overview authors conducted the GRADE assessment independently. Disagreements were resolved by discussion until consensus was reached; if needed, a third overview author was called to arbitrate.

### Selection in case of duplicated comparisons and outcomes

If more than one SR reported evidence for the same procedure-outcome pair, we applied the following selection rules. The selection of reviews was conducted at outcome level:

1. We prioritized direct over indirect evidence: if one SR reported evidence specifically on CS and another SR reported on any surgeries (including CS), we selected the SR with evidence specifically on CS.
2. When evidence was available from a Cochrane review (CR) and a non-Cochrane review (NCR) and the search dates of the reviews were <24 months apart, we selected the CR.
3. When evidence was available from a CR and a NCR and the NCR was more recent than the CR (search date difference ≥24 months), we selected the NCR.
4. When evidence was available from two or more NCRs:
  4.1 We selected the most recent review (search date ≥24 months apart).
  4.2 If search dates were <24 months apart, we selected the NCR with the highest GRADE assessment for the outcome of interest.
  4.3 If no GRADE assessment was reported in one of the reviews, we selected the NCR with the GRADE available for the outcome of interest.
  4.4 If the outcome had the same GRADE assessment in both reviews, we selected the SR with the highest AMSTAR 2 score.
5. When evidence was available from two or more CRs, we applied the same rules described for NCR.
6. For reviews with different search dates (≥24 months apart), but including the same studies, we selected the review with the highest AMSTAR 2 score.
7. For SRs with network meta-analysis, only direct comparisons were included and the same selections rules were applied.

### Data synthesis

We defined our unit of analysis as the “procedure-outcome” pair. Examples would be single versus double layer for uterine closure (procedure) and blood loss (outcome); or changing gloves (procedure) and endometritis (outcome).

We structured data synthesis as in other overviews.^25^, ^26^, ^27^, ^28^ We classified each procedure-outcome pair into one of eight possible categories according to the pooled effect estimate and the certainty of the evidence. Box 1 presents the definitions of each category and corresponding standardised statements used in the text based on the recommendations of the Cochrane Effective Practice and Organisation of Care (EPOC)^29^ and GRADE working group.^30^ Categorization was conducted independently by two reviewers. Disagreements were resolved through discussion; when consensus was not reached, a third reviewer was called to arbitrate.

Box 1 **Categories for classification of each procedure-outcome pair in this overview (based on EPOC,29 GRADE30 and previous publications25–28).**

**Table:** 

Category		Effect estimate (RR/OR/WMD)	Certainty of the evidence	Terminology in text
	Clear evidence of benefit	Effect of benefit and the 95% CI not crossing the line of no effect	High or moderate	High certainty: *“Reduces/Increases …”* Moderate certainty: *“Probably reduces/increases …”*
	Possible benefit	Effect of benefit and the 95% CI not crossing the line of no effect	Low	*“May reduce/increase… “*
	Clear evidence of no difference of effect	Effect near the line of no effect and a narrow 95% CI crossing the line of no effect between 0.75 and 1.25	High or moderate	*“Have no effect …”*
	Possible evidence of no difference of effect	Effect near the line of no effect and a narrow 95% CI crossing the line of no effect between 0.75 and 1.25	Low	*“May have no effect …”*
	Clear evidence of harm	Effect of harm and the 95% CI not crossing the line of no effect	High or moderate	High certainty: *“Reduces/Increases …”* Moderate certainty: *“Probably reduces/increases...”*
	Possible Harm	Effect of harm and the 95% CI not crossing the line of no effect	Low	*“May reduce/increase …”*
	No conclusion possible	Any effect estimates and a wide 95% CI crossing the line of no effect substantially	High, moderate or low	*“There is insufficient evidence …”*
		Any effect estimates	Very low	*“It is uncertain whether …”*
	No systematic review	Not applicable	Not applicable	*“No systematic review …”*

CI, Confidence interval; OR, Odds ratio; RR, relative risk; WMD, weighted mean difference.

### Ethics

Ethical approval was not required because all data included is available in the public domain.

### Statistics

No statistical analysis was conducted in this overview.

### Role of funding source

The funders of this overview of systematic reviews had no role in the overview design, data collection, data analysis, data interpretation, or writing of the manuscript.

## Results

**Fig. 1:**
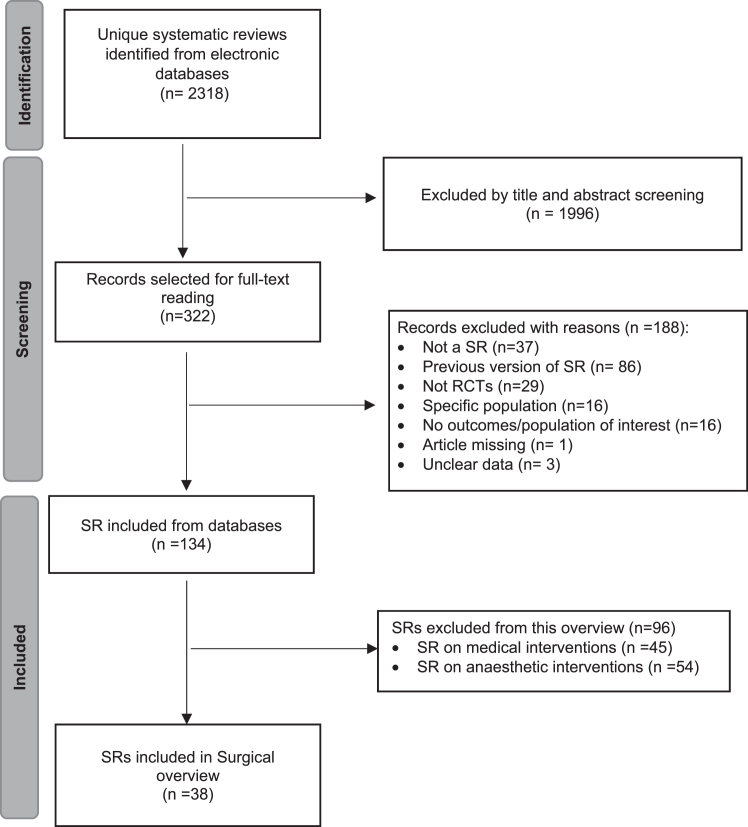
Flow diagram of the process of identification and selection of systematic reviews on surgical procedures for caesarean section.

We identified 2318 unique records from electronic databases. We excluded 1996 records by screening titles and abstracts, and selected 322 for full text evaluation. After exclusions (Annex 3) and identification of additional reviews from reference lists, 38 SRs assessing different surgical procedures at CS were included in the overview (Fig. 1).

### Description of included reviews

The overview includes 38 SRs^31^, ^32^, ^33^, ^34^, ^35^, ^36^, ^37^, ^38^, ^39^, ^40^, ^41^, ^42^, ^43^, ^44^, ^45^, ^46^, ^47^, ^48^, ^49^, ^50^, ^51^, ^52^, ^53^, ^54^, ^55^, ^56^, ^57^, ^58^, ^59^, ^60^, ^61^, ^62^, ^63^, ^64^, ^65^, ^66^, ^67^, ^68^ (16 Cochrane and 22 non-Cochrane) published between 2004–2024 involving 628 RCTs with a total of 190,349 participants. Table 1 summarises the main characteristics of the included SRs (see Annex 4 for details). About half of the SRs were conducted in the last five years (n = 21), included more than 10 RCT (n = 18), and included studies conducted in LMICs (n = 17). Most of the SRs included any type of CS (emergency or elective) (n = 27). Almost 80% of the SRs (N = 30) were rated as being of low or critically low methodological quality according to the AMSTAR 2 tool.

**Table 1:** Main characteristics of 38 systematic reviews on surgical procedures related to caesarean section.

Characteristic	N (%)	References
**Type of SR**		
Cochrane	16 (42%)	Anderson 2004, Anorlu 2008, Bamigboye 2014, Charoenkwan 2017, Dodd 2014, Dumville 2016, Gates 2013, Hadiati 2020, Hofmeyr 2008, Jacobs 2004, Liabsuetrakul, Mackeen 2012, Mathai 2013, Norman 2017, Norman 2022, Waterfall 2016
Non-Cochrane	22 (58%)	Agarwal 2020, Bhat 2022, Cornthwaite 2023, Eke 2015, Khanuja 2022, Mackeen 2022, Narice 2020, O'Neil 2014, Pergialiotis 2017, Pergialiotis 2021, Pergialiotis 2022, Quayum 2021, Raischer 2022, Rattanakanokchai 2021, Roberge 2014, Saad 2014, Sobodu 2024, Tan 2021, Waring 2018, Wijetunge 2021, Zaman 2021
**Year of publication**		
Prior to 2013	4 (11%)	Anderson 2004, Anorlu 2008, Hofmeyr 2008, Jacobs 2004,
2013–2017	13 (34%)	Bamigboye 2014, Charoenkwan 2017, Dodd 2014, Dumville 2016, Eke 2015, Gates 2013, Mackeen 2012, Mathai 2013, O'Neil 2014, Pergialiotis 2017, Roberge 2014, Saad 2014, Waterfall 2016,
2018–2024	21 (55%)	Agarwal 2020, Bhat 2022, Cornthwaite 2023, Hadiati 2020, Khanuja 2022, Liabsuetrakul 2018, Mackeen 2022, Narice 2020, Norman 2017, Norman 2022, Pergialiotis 2021, Pergialiotis 2022 Quayum 2021, Raischer 2022, Rattanakanokchai 2021, Tan 2021, Waring 2018, Wijetunge 2021, Ye Huang 2022, Zaman 2021
**Number of trials**		
0–5	7 (19%)	Agarwal 2020, Eke 2015, Mathai 2013, O'Neil 2014, Raischer 2022, Saad 2014, Sobodu 2024
6–10	13 (34%)	Anderson 2004, Gates 2013, Jacobs 2004, Khanuja 2022, Liabsuetrakul 2018, Mackeen 2012, Narice 2020, Pergialiotis 2017, Pergialiotis 2021, Rattanakanokchai 2021, Waring 2018, Waterfall 2016, Wijetunge 2021
>10	18 (47%)	Anorlu 2008, Bamigboye 2014, Bhat 2022, Cornthwaite 2023, Charoenkwan 2017, Dodd 2014, Dumville 2016, Hadiati 2020, Hofmeyr 2008, Mackeen 2022, Norman 2017, Norman 2022, Pergialiotis 2022, Quayum 2021, Roberge 2014, Tan 2021, Ye Huang 2022, Zaman 2021
**Total N participants**		
<5000	23 (61%)	Agarwal 2020, Anderson 2004, Anorlu 2008, Charoenkwan 2017, Cornthwaite 2023, Eke 2015, Hofmeyr 2008, Jacobs 2004, Liabsuetrakul 2018, Mackeen 2012, Mackeen 2022, Mathai 2013, Narice 2020, O'Neil 2014, Pergialiotis 2017, Pergialiotis 2021, Raischer 2022, Rattanakanokchai 2021, Saad 2014, Sobodu 2024, Waring 2018, Waterfall 2016, Wijetunge 2021, Zaman 2021
5001–10000	8 (21%)	Dumville 2016, Gates 2013, Hadiati 2020, Khanuja 2022, Norman 2017, Pergialiotis 2022, Ye Huang 2022
>10,000	7 (18%)	Bamigboye 2014, Bhat 2022, Dodd 2014, Norman 2022, Quayum 2021, Roberge 2014, Tan 2021
**Included trials from LMICs**		
Yes	17 (45%)	Anderson 2004, Bamigboye 2014, Bhat 2022, Cornthwaite 2023, Charoenkwan 2017, Dodd 2014, Hadiati 2020, Khanuja 2022, Liabsuetrakul 2018, Mathai 2013, Narice 2020, Norman 2017, Quayum 2021, Sobodu 2024, Rattanakanokchai 2021, Roberge 2014, Zaman 2021
No	11 (29%)	Agarwal 2020, Anorlu 2008, Dumville 2016, Eke 2015, Jacobs 2004, Norman 2022, Pergialiotis 2021, Saad 2014, Waring 2018, Wijetunge 2021, O'Neil 2014
Unclear	10 (26%)	Gates 2013, Hofmeyr 2008, Mackeen 2012, Mackeen 2022, Pergialiotis 2017, Pergialiotis 2022, Raischer 2022, Tan 2021, Waterfall 2016, Ye Huang 2022
**Type of CS included in SR**		
Elective only	3 (8%)	Charoenkwan 2017, Liabsuetrakul 2018, O'Neil 2014
Elective and emergency	27 (71%)	Agarwal 2020, Anderson 2004, Anorlu 2008, Bamigboye 2014, Cornthwaite 2023, Dodd 2014, Eke 2015, Gates 2013, Hadiati 2020, Hofmeyr 2008, Jacobs 2004, Khanuja 2022, Mackeen 2012, Narice 2020, Norman 2017, Pergialiotis 2017, Pergialiotis 2022, Quayum 2021, Rattanakanokchai 2021, Roberge 2014, Saad 2014, Sobodu 2024, Tan 2021, Waterfall 2016, Wijetunge 2021, Ye Huang 2022, Zaman 2021
Unclear	8 (21%)	Bhat 2022, Dumville 2016, Mackeen 2022, Mathai 2013, Norman 2022, Pergialiotis 2021, Raischer 2022, Waring 2018
**Quality of SR (AMSTAR 2 score)**		
Critically low	18 (47%)	Agarwal 2020, Anderson 2004, Anorlu 2008, Bhat 2022, Eke 2015, Jacobs 2004, Khanuja 2022, Pergialiotis 2017, Quayum 2021, Raischer 2022, Rattanakanokchai 2021, Roberge 2014, Saad 2014, Tan 2021, Waring 2018, Ye Huang 2022, Zaman 2021, O'Neil 2014
Low	12 (32%)	Bamigboye 2014, Dodd 2014, Gates 2013, Hofmeyr 2008, Mackeen 2012, Mackeen 2022, Mathai 2013, Pergialiotis 2021, Pergialiotis 2022, Sobodu 2024, Waterfall 2016, Wijetunge 2021
Moderate	3 (8%)	Cornthwaite 2023, Dumville 2016, Narice 2020
High	5 (13%)	Charoenkwan 2017, Hadiati 2020, Liabsuetrakul 2018, Norman 2017, Norman 2022

### Summary of effects

We retrieved 384 procedure-outcome comparisons from the included SRs. After excluding 39 overlapping comparisons, we present the effects of 345 procedure-outcomes pairs: 237 compared two different procedures, and 108 compared a procedure versus no treatment or placebo (NT/P).

Among the 345 comparisons, there was insufficient or inconclusive evidence of any effect for 256 comparisons. We found 40 comparisons with clear evidence of benefit (27 procedure versus procedure, 13 procedure versus NT/P), and 17 comparisons with evidence of a possible benefit (10 procedure versus procedure, 7 procedure versus NT/P). For 12 comparisons, there was clear evidence of no difference of effect (8 procedure versus procedure, 4 procedure versus NT/P), and for 1 procedure versus procedure comparison, there was evidence of possible no difference of effect. Finally, for 14 comparisons, there was clear evidence of harm (10 procedure versus procedure, 4 procedure versus NT/P), and for 5 comparisons, there was evidence of possible harm (4 procedure versus procedure, 1 procedure versus NT/P).

Table 2, Table 3, Table 4, Table 5, Table 6, Table 7, Table 8 summarize results for each procedure-outcome comparisons following the chronological order of surgical procedures. Annex 5 provides details for all comparisons, outcomes, estimates and references. For 7 pre-defined procedures, there were no SRs.

**Table 2:** Preparation for abdominal wall opening in caesarean section.

Procedure	Control	Outcome	Effect estimate [CI] (No. RTC/No. participants)	Certainty of evidence	Category
**Preparation**					
**Skin cleansing**^31^					
Chlorhexidine gluconate	Povidone iodine	SSI	0.72 [0.58, 0.91] (8/4323)	Moderate	
		Adverse events^a^	0.64 [0.28, 1.46] (3/1926)	Very low	
		Endometritis	0.95 [0.49, 1.86] (2/2484)	Low	
Chlorhexidine plus alcohol	Povidone iodine plus alcohol	SSI	0.62 [0.45, 0.87] (4/2663)	Low	
Chlorhexidine plus alcohol	Povidone iodine	SSI	0.84 [0.61, 1.15] (4/1660)	Very low	
Parachlorometa-xylenol with iodine	Iodine alone	SSI	0.33 [0.04, 2.99] (1/50)	Very low	
		Endomyometritis	0.88 [0.56, 1.38] (3/2484)	Very low	
**Adhesive drape**^31^					
Drape	No drape	SSI	1.29 [0.97, 1.71] (3/1373)	Low	
		Metritis	1.62 [0.29, 9.16] (1/79)	Very low	
		Length of stay (days)	0.10 [−0.27, 0.46] (1/603)	Moderate	
Drape: Chlorhexidine	No drape	SSI	1.11 [0.70, 1.76] (1/603)	Moderate	
Drape: Iodine	No drape	SSI	1.42 [0.98, 2.04] (1/691)	Very low	

CI, Confidence interval; RCT, Randomised controlled trial; SSI, Surgical site infection.

a Adverse events: organ damage, blood transfusion, sepsis, thromboembolism, organ failure, admission to high care unit or death.

**Table 3:** Techniques and materials for opening the abdominal wall in caesarean section.

Procedure	Control	Outcome	Effect estimate [CI] (No. RTC/No. participants)	Certainty of evidence	Category
**Abdominal incision**					
**Individual layer opening (technique and material)**					
**Combined layers opening**^32^^,^^33^					
Joel-Cohen-based incision	Pfannenstiel incision	Fever	0.47 [0.28, 0.81] (8/1412)	High	
		Blood loss (ml)	−64.45 [−91.34, −37.56] (4/481)	High	
		Time to oral intake (h)	−3.92 [−7.13, −0.71] (4/481)	Moderate	
		Postoperative stay (days)	−0.99 [−1.44, −0.54] (3/323)	Moderate	
		Operating time (min)	−18.65 [−24.84, −12.45] (4/481)	Moderate	
		Skin incision to delivery (min)	−3.84 [−5.41, −2.27] (4/575)	Moderate	
		Endometritis	0.34 [0.01, 8.17] (3/767)	Low	
		Blood transfusion	4.08 [0.46, 36.4] (3/681)	Low	
		Wound infection	1.43 [0.52, 3.91] (6/1071)	Moderate	
		Time to mobilization (h)	−2.86 [−11.29, 5.56] (2/208)	Low	
		Serious complications^a^	1.31 [0.29, 5.91] (4/913)	Low	
		Apgar score <7 at 5 min.	0.18 [0.01, 3.71] (1/158)	Low	
		NICU admission	1.19 [0.44, 3.20] (1/310)	Low	
		Postoperative pain	−14.18 [−18.31, −10.04] (1/172)	Very low	
Joel-Cohen-based (Modified Misgav-Ladach)	Lower midline incision	Blood loss (ml)	−93 [−132.72, −53.28] (1/339)	Moderate	
		Operating time (min)	−7.3 [−8.32, −6.28] (1/339)	Moderate	
		Time to mobilization (hours)	−16.06 [−18.22, −13.90] (1/339)	Moderate	
		Postoperative hospital stay (days)	−0.82 [−1.08, −0.56] (1/339)	Moderate	
		Fever	1.38 [0.75, 2.54] (1/339)	Low	
		Wound infection	1.14 [0.68, 1.91] (1/339)	Moderate	
		Postoperative anaemia	0.60 [0.22, 1.62] (1/339)	Low	
		Endometritis	2.50 [0.49, 12.74] (1/400)	Very low	
Muscle-cutting/Maylard incision	Pfannenstiel incision	Postoperative febrile morbidity	1.26 [0.08, 19.50] (1/97)	Very low	
		Wound infection	1.26 [0.27, 5.91] (1/97)	Very low	
		Blood transfusion	0.42 [0.02, 9.98] (1/97)	Very low	
		Postoperative hospital stay (days)	0.40 [−0.34, 1.14] (1/97)	Very low	
		Long-term complication	0.10 [−0.73, 0.93] (1/54)	Very low	
**Extraperitoneal technique**^33^					
Extraperitoneal CS	Intraperitoneal CS	Fever	0.42 [0.27, 0.65] (1/412)	High	
		Serious complications	0.12 [0.02, 0.88] (1/412)	Moderate	
		Maternal mortality	0.17 [0.02, 1.37] (1/412)	Low	
		Repeat wound operative procedures	1.5 [0.7, 3.2] (1/412)	Low	
**Type of scalpel (all layers)**^34^					
Scalpel	Electrosurgery	Wound infection^b^	1.07 [0.74, 1.54] (11/2178)	Very Low	
		Wound dehiscence^b^	1.21 [0.58, 2.50] (6/1064)	Very Low	

CI, Confidence interval; CS, Caesarean section; RCT, randomized controlled trial; SSI, Surgical site infection.

a Serious complications: organ damage, blood transfusion, sepsis, thromboembolism, organ failure, admission to high care unit or death.

b Estimates refer to all studies and all participants included in the systematic review but only 1 study with 130 participants involved patients undergoing a caesarean section.

**Table 4:** Intraabdominal procedures in caesarean section.

Procedure	Control	Outcome	Effect estimate [CI] (No. RTC/No. participants)	Certainty of evidence	Category
**Preparation**					
**Use of retractors**^35^					
O'ring retractor	Standard care	Need for uterine exteriorization	0.48 [0.33, 0.69] (4/1301)	Moderate	
		Adequate visualization	1.01 [1.04, 1.16] (2/352)	Moderate	
		SSI	0.76 [0.34, 1.79] (5/1515)	Low	
**Use of aspiration device**					
**Use of surgical swabs**					
**Bladder flap**^36^					
Bladder flap formation	Non formation	Skin incision to delivery (min)	1.27 [0.63, 1.92] (3/466)	Very Low	
		Blood loss (ml)	42.40 [−32.30, 117.0] (3/479)	Very Low	
		Bladder injury	0.96 [0.19, 4.84] (3/479)	Very Low	
		Total surgical time (min)	3.50 [−0.19, 7.16] (4/581)	Very Low	
		Duration of hospitalization (days)	0.07 [−0.50, 0.64] (2/364)	Very Low	
**Uterine incision**					
**Hysterotomy technique (location/length)**					
**Hysterotomy expansion**^37^, ^38^, ^39^					
Blunt dissection/expansion	Sharp dissection/expansion	Operative time (min)	−2.06 [−2.11, −2.01] (2/1276)	High	
		Mean blood loss (ml)	−55.00 [−79.48, −30.52] (2/1145)	Moderate	
		Blood transfusion	0.24 [0.09, 0.62] (2/1345)	Low	
		Febrile morbidity (incl. endometritis)	0.86 [0.70, 1.05] (4/1941)	Low	
		Maternal death or serious morbidity	3.00 [0.12, 73.20] (1/400)	Very Low	
		Unintended incision extension	0.47 (0.28, 0.79) (5/2608)	Very Low	
Cephalad-caudal expansion	Transversal expansion	Unintended incision extension	0.62 [0.45, 0.86] (6/2818)	Moderate	
Transverse blunt expansion	Cephalad-caudad blunt expansion	Mean blood loss (ml)	42.00 [1.31, 82.69] (1/811)	Moderate	
		Additional sutures	0.62 [0.31, 1.23) (4/1869)	Low	
		Blood transfusion	0.75 [0.28, 2.03] (4/2208)	Very Low	
		Duration of surgery (min)	−1.50 [−3.13, 0.13] (1/811)	Very Low	
**Materials for uterine incision**^37^					
Auto stapler	Conventional incision	Febrile morbidity	0.92 [0.38, 2.20] (2/300)	Very Low	
		Endometritis	0.20 [0.02, 1.65] (1/100)	Very Low	
		Blood transfusion	1.50 [0.26, 8.60] (1/100)	Very Low	
		Mean blood loss (ml)	−87.00 [−175.09, 1.09] (1/200)	Very Low	
		Duration of surgery (min)	3.30 [−0.02, 6.62] (1/197)	Very Low	
		Postnatal hospital stay (days)	0.00 [−0.28, 0.28] (1/200)	Very Low	
		Wound complications	1.5 [0.67, 3.35] (1/100)	Very Low	
**Technique forfoetal andplacentalextraction**					
**Technique for foetal extraction**					
**Techniques and instruments for assisting difficult foetal extraction**^40^^,^^41^					
Reverse breech	Head push	Wound infection	0.96 [0.58, 1.59] (4/357)	Low	
		Mean blood loss (ml)	−294.92 [−493.25, −96.59] (3/298)	Very Low	
		Operative time (min)	−14.99 [−27.67, −2.30] (4/357)	Very Low	
		Endometritis	0.52 [0.26, 1.05] (3/285)	Very Low	
		Blood transfusion	0.57 [0.20, 1.66] (2/177)	Very Low	
		Mean hospital stay (days)	−1.13 [−2.75, 0.48] (3/285)	Very Low	
		Infant birth trauma	1.55 [0.42, 5.73] (3/239)	Very Low	
		NICU Admission	0.53 [0.23, 1.22] (2/226)	Very Low	
		Early neonatal death	0.54 [0.23, 1.24] (1/108)	Very low	
		Average Apgar at 5 min	0.36 [−0.64, 1.36] (3/239)	Very Low	
Head push	Reverse breech	Extension of uterine incision	3.45 [2.41, 4.93] (7/739)	Low	
Tocolysis	Placebo	Maternal side-effects	NE (1/97)	Low	
Elective instrument	Fundal pressure	Infant birth trauma	NE (1/44)	Very Low	
		Extension of uterine incision	0.70 [0.13, 3.73] (1/44)	Very low	
Fetal pillow	No fetal pillow	Blood loss >1000 mL	0.19 [0.08, 0.48] (1/240)	Very Low	
		Operative time (min)	−21.20 [−23.20, −19.20] (1/240)	Low	
		Neonatal intensive care admission	0.62 [0.33, 1.18] (1/240)	Very low	
		Apgar score <3 at 5 min	0.43 [0.04, 4.33] (4/414)	Very low	
		Incision to delivery interval	−120.7 [−126.2, −115.2] (1/240)	Low	
Inflated fetal pillow	Non-inflated fetal pillow	Uterine incision extension	0.46 [0.2, 1.05] (1/60)	Low	
		Blood transfusion	0.13 [0.01, 1.26] (1/60)	Low	
		Postpartum pyrexia/sepsis	1.2 [0.41, 3.51] (1/60)	Low	
**Timing for umbilical cord clamping**					
**Placental extraction**^42^^,^^43^					
Manual placental removal	Cord traction/Spontaneous delivery	Endometritis	1.64 [1.42, 1.90] (13/4134)	High	
		Postoperative hospital stay (days)	0.39 [0.17, 0.61] (3/546)	High	
		Fever	1.14 [0.63, 2.08] (2/580)	Moderate	
		Blood loss	149.18 [−32.55, 330.92] (11/2678)	Low	
		Intraoperative duration	−0.89 [−2.34, 0.57] (12/2985)	Very low	
		Haemorrhage^a^	1.83 [1.20, 2.78] (3/1156)	High	
		Blood transfusion	1.20 [0.63, 2.28] (7/2508)	Moderate	
		Infectious morbidity	1.82 [0.94, 3.52] (10/3359)	Low	
**Other procedures**					
**Mechanical cervical dilatation**^44^					
Manual cervical dilatation	No dilatation	Blood loss (mL)	−48.49 [−88.75, −8.23] (1/400)	High	
		Retained products of conception	0.04 [0.00, 0.63] (1/447)	Moderate	
		Infectious morbidity	0.91 [0.51, 1.60] (1/400)	High	
		Haemoglobin drop (g/dL)	0.92 [0.64, 1.31] (2/722)	Low	
		Febrile morbidity	1.18 [0.76, 1.85] (7/2126)	Low	
		Wound infection	1.13 [0.44, 2.90] (5/1719)	Low	
		Urinary tract infection	0.92 [0.34, 2.53] (2/847)	Low	
		Operative time (min)	−0.05 [−2.62, 2.53] (4/1585)	Low	
		PPH ≥1000 mL	1.97 [0.48, 8.13] (1/47)	Very low	
		Blood transfusion	3.54 [0.37, 33.79] (2/847)	Very low	
		Secondary PPH	1.18 [0.07, 18.76] (1/447)	Very low	
		Endometritis	0.94 [0.35, 2.52] (4/1536)	Very low	
		Uterine subinvolution	0.34 [0.08, 1.36] (2/654)	Very low	
**Uterine cleansing**					
**Changing gloves**^45^^,^^46^					
Changing gloves at any time	Not changing gloves	SSI	0.41 [0.26, 0.65] (4/1036)	High	
		Endometritis	0.96 [0.78, 1.20] (5/1706)	High	
		Febrile morbidity	0.73 [0.30, 1.81] (3/744)	Moderate	
Changing gloves before placenta delivery	Not changing gloves	Endometritis	1.00 [0.80, 1.26] (3/979)	Moderate	
		Fever	1.30 [0.67, 2.49] (2/208)	Low	
		SSI	0.62 [0.15, 2.49] (2/208)	Very low	
Changing gloves after placenta delivery	Not changing gloves	SSI	0.39 [0.24, 0.63] (3/678)	High	
		Febrile morbidity	0.45 [0.19, 1.04] (2/586)	Low	
		Endometritis	0.63, [0.30, 1.36] (2/726)	Low	

BMI, Body Mass Index; CI, Confidence interval; NE, Not estimable; NICU, Neonatal Intensive Care Unit; PPH, Postpartum haemorrhage; RCT, Randomised controlled trial; SSI, Surgical site infection.

a Using fixed effects model.

**Table 5:** Uterine closure and other intraabdominal procedures in caesarean section.

Procedure	Control	Outcome	Effect estimate [CI] (No. RTC/No. participants)	Certainty of evidence	Category
**Uterine closure**					
**Technique**^37^^,^^47^, ^48^, ^49^, ^50^, ^51^, ^52^					
Single layer	Double layer	Duration of caesarean (min)	−2.25 [−3.29, −1.21] (10/6598)	Moderate	
		Febrile morbidity (including endometritis)	0.98 [0.85, 1.12] (9/13,890)	High	
		Wound infection	0.99 [0.89, 1.10] (5/13,389)	High	
		Dysmenorrhea	1.36 [1.02, 1.81] (4/7847)	High	
		Maternal infectious morbidity	0.94 [0.66, 1.34] (6/4844)	Moderate	
		Endometritis	1.04 [0.81, 1.34] (8/13,815)	Low	
		Blood loss	7.14 [−16.21, 30.50] (9/3106)	Low	
		Blood loss >500 mL	0.70 [0.42, 1.18] (1/339)	Low	
		Blood transfusion	0.86 [0.63, 1.17] (4/13,571)	Moderate	
		Operative procedure on wound	0.80 [0.53, 1.21] (3/12,604)	Moderate	
		Relaparotomy	0.85 [0.63, 1.16] (1/9286)	Moderate	
		Readmission rate	0.95 [0.64, 1.40] (2/5007)	Low	
		Complications in future pregnancy	3.21 [0.13, 77.55] (1/145)	Very low	
		Uterine dehiscence	1.88 [0.63, 5.62] (3/2379)	Low	
		Postoperative pain	0.88 [0.54, 1.42] (2/9444)	Low	
		Death or serious morbidity	1.04 [0.71, 1.54] (3/12,665)	Moderate	
		Hospital stay (days)	−0.12 [−0.30, 0.06] (6/5774)	Low	
Double-layer suture with the first layer locked	Double-layer suture with unlocked first layer	Risk of uterine scar dehiscence at next caesarean	2.14 [0.22, 21.10] (1/29)	Very low	
Uterine exteriorization	Intraperitoneal repair	Febrile morbidity >3 days	0.41 [0.17, 0.97] (1/308)	Low	
		Satisfaction with operation	0.92 [0.82, 1.04] (1/139)	Low	
		Pain at 6 h	1.64 [1.31, 2.03] (2/1637)	High	
		Intraoperative nausea and vomiting	2.09 [1.66, 2.63] (4/1454)	Moderate	
		Intraoperative nausea	1.08 [0.82–1.42] (7/2343)	Moderate	
		Intraoperative vomiting	1.94 (0.69–1.28) (6/2634)	Low	
		Endometritis	1.22 [0.96, 1.55] (11/18,339)	Low	
		Sepsis	0.94 [0.19, 4.57] (1/308)	Low	
		Wound infection	1.01 [0.71, 1.45] (11/18,662)	Low	
		Operative blood loss (mL)	17.11 [−23.15, 57.37] (6/504)	Moderate	
		Blood transfusion	1.11 [0.63, 1.94] (10/18,429)	Moderate	
		Blood loss (mL)	−40.8 [−90.42, 8.82] (9/2208)	Low	
		Intra-operative pain	1.76 [0.97, 3.20] (5/704)	Moderate	
		Nausea	1.18 [0.78, 1.80] (3/667)	Low	
		Postoperative nausea	1.36 [0.86, 2.14] (3/1975)	Moderate	
		Duration of operation (min)	1.70 [−0.72, 4.12] (16/19,399)	Very low	
		Hospital stay (days)	0.16 [−0.08, 0.41] (10/17,340)	Low	
		Hypotension	1.42 [0.90, 2.22] (6/2573)	Low	
Intraperitoneal repair	Uterine exteriorization	Return to bowel function	−0.76 [−1.36, −0.15] (6/7696)	Moderate	
		Rescue analgesia	0.44 [0.28, 0.68] (6/17,591)	Moderate	
**Suture material**^37^^,^^49^^,^^53^^,^^54^					
Multifilament suture	Monofilament suture	Endometritis	Not estimable (1/95)	Very Low	
		Wound infection	Not estimable (1/95)	Very Low	
		Maternal infection	Not estimable (1/95)	Very Low	
		Postpartum haemorrhage	7.09 [0.4, 136.2] (3/489)	Very Low	
		Blood transfusion	Not estimable (2/189)	Very Low	
		Uterine rupture	Not estimable (1/95)	Very Low	
		Uterine dehiscence	Not estimable (1/95)	Very Low	
Chromic suture	Multifilament suture	Blood transfusion	0.53 [0.30, 0.93] (1/9184)	High	
		Relaparotomy	0.58 [0.37, 0.89] (1/9544)	High	
		Febrile morbidity (including endometritis)	0.70 [0.49, 1.00] (1/9544)	Moderate	
		Endometritis	0.83 [0.49, 1.40] (1/9184)	Moderate	
		Wound infection	1.07 [0.84, 1.36] (1/9184)	Moderate	
		Maternal infection	0.33 [0.02, 6.52] (1/9184)	Moderate	
		Use of additional uterotonics	0.50 [0.22, 1.13] (1/9184)	Moderate	
		Uterine rupture	3.05 [0.32, 29.29] (1/3306)	Moderate	
		Uterine dehiscence	0.67 [0.11, 4.02] (1/3306)	Moderate	
		Operation time (min)	Not reported	Moderate	
		Operative procedure on the wound	0.64 [0.36, 1.13] (1/9544)	Moderate	
		Readmission	1.00 [0.58, 1.72] (1/9544)	Moderate	
		Postoperative pain	0.86 [0.70, 1.07] (1/9544)	Moderate	
		Maternal mortality or serious morbidity	0.68 [0.44, 1.06] (1/9544)	Moderate	
Non-barbed suture (Multifilament)	Barbed suture	Uterine repair time (min)	1.8 [1.6, 2.1] (3/272)	Moderate	
		Operation time (min)	1.9 [0.03, 3.8] (3/272)	Moderate	
		Endometritis	Not estimable (2/172)	Very Low	
		Wound infection	Not estimable (2/172)	Very Low	
		Maternal infection	0.3 [0.01, 7.9] (2/202)	Low	
		Estimated blood loss (mL)	46.17 [−13.55, 105.89] (3/272)	Very low	
		Use of additional uterotonics	0.9 [0.47, 1.79] (1/70)	Very Low	
		Blood transfusion	3.0 [0.3, 28.3] (2/136)	Low	
		Uterine rupture	Not estimable (1/102)	Very low	
		Uterine dehiscence	Not estimable (1/102)	Very low	
Barbed suture	Non-barbed (conventional suture)	Additional haemostatic sutures	0.39 [0.28, 0.54] (3/272)	Low	
		Combined postoperative morbidity	0.96 [0.46, 2.00] (3/272)	Very low	
**Other intraabdominal procedures**					
**Abdominal irrigation**^55^^,^^56^					
Irrigation (any type)	No irrigation	SSI	0.87 [0.68, 1.11] (14/6106)	Low	
		Abscess	0.91 [0.54, 1.54] (3/331)	Moderate	
		Reoperation	0.72 [0.28, 1.84] (2/3247)	Low	
		Maternal readmission	0.70 [0.10, 4.90] (2/3247)	Low	
		Adverse events	1.05 [0.76, 1.44) (3/403)	Low	
		Maternal mortality	0.86 [0.36, 2.04] (2/280)	Very Low	
		Hospital stay (days)	−0.13 [−0.38, 0.12] (7/1597)	Very Low	
Warm saline instillation	No treatment	Postoperative nausea	1.92 [1.37, 2.69) (2/666)	Low	
		Intraoperative nausea	1.68 [1.36, 2.06] (2/666)	High	
		Intraoperative emesis	1.70 [1.28, 2.25] (2/666)	High	
		Postoperative anti-emetics	1.84 [1.21, 2.78] (2/666)	High	
		Endometritis	0.95 [0.64, 1.40] (3/862)	Moderate	
		Wound infection	0.51 [0.09, 2.73] (2/626)	Low	
		Urinary tract infection	0.92 [0.66, 1.30] (2/626)	Moderate	
		Blood loss (mL)	−8.10 [−20.95, 4.47] (3/862)	Moderate	
		Operative time (min)	0.06 [−5.16, 5.29] (2/666)	Moderate	
		Postoperative emesis	1.65 [0.74.3.67) (2/666)	Moderate	
Antibacterial irrigation	Non-antibacterial irrigation	SSI	0.57 [0.44, 0.75] (30/5141)	Low	
		Hospital stay (days)	−0.85 [−1.60, −0.09] (7/635)	Low	
		Adverse events	0.55 [0.22, 1.34] (3/178)	Low	
		Maternal mortality	0.81 [0.48, 1.36] (11/1121)	Very Low	
		Wound dehiscence	1.26 [0.65, 2.45] (3/660)	Very low	
		Reoperation	1.26 [0.12, 13.60] (2/403)	Very Low	
Icodextrin	Lactated Ringer's solution	Adverse events	0.99 [0.96, 1.02] (2/875)	Moderate	
		Maternal mortality	0.0 [0.0, 0.0] (2/875)	Very low	
Standard irrigation	Pulsatile irrigation	SSI	0.34 [0.19, 0.62] (2/484)	Low	

SSI, Surgical site infection; NICU, Neonatal Intensive Care Unit; PPH, Postpartum haemorrhage; BMI, Body Mass Index; CS, caesarean section.

**Table 6:** Abdominal wall closure in caesarean section: peritoneum closure.

Procedure	Control	Outcome	Effect estimate [CI] (No. RTC/No. participants)	Certainty of evidence	Category
**Peritoneum closure**^57^					
Non-closure of parietal peritoneum only	Closure of both peritoneal layers	Postoperative pain	0.45 [0.31, 0.66] (1/325)	Moderate	
		Mobilization time (hours)	−1.89 [−3.18, −0.60] (1/110)	Moderate	
		Time to oral intake (hours)	−2.31 [−3.76, −0.86] (1/110)	Moderate	
		Operating time (min)	−5.10 [−8.71, −1.49] (1/248)	Very low	
		Fever	0.18 [0.01, 3.56] (1/40)	Low	
		Blood loss (mL)	56.97 [−28.08, 142.02] (1/110)	Low	
		Drop in haemoglobin (g/dL)	0.28 [−0.03, 0.59] (1/110)	Moderate	
		Endometritis	0.88 [0.53, 1.46] (1/248)	Very Low	
		Wound infection	0.95 [0.14, 6.66] (1/248)	Very low	
		Postoperative hospital stay (days)	−0.15 [−1.20, 0.91] (2/288)	Very Low	
		Time to flatus (hours)	−0.04 [−1.99, 1.91] (1/110)	Very low	
Non-closure of visceral peritoneum only	Closure of both peritoneal layers	Urinary frequency at 8 weeks	0.24 [0.13, 0.45] (1/582)	High	
		Urgency of urination	0.30 [0.18, 0.51] (1/582)	High	
		Stress incontinence	0.45 [0.21, 0.96] (1/582)	High	
		Operating time (min)	−6.30 [−9.22, −3.38] (1/544)	Low	
		Postoperative hospital stay (days)	−0.70 [−0.98, −0.42] (1/549)	Low	
		Wound infection	0.36 [0.14, 0.89] (2/789)	Very low	
		Adhesions	2.49 [1.49, 4.16] (2/157)	Very low	
		Postoperative fever	0.60 [0.29, 1.27] (3/889)	Very low	
		Endometritis	3.00 [0.12, 72.91] (1/240)	Very low	
Non-closure of both peritoneal layers	Closure of both peritoneal layers	Chronic pelvic pain	0.49 [0.25, 0.98] (1/112)	Low	
		Postoperative hospital stay (days)	−0.26 [−0.47, −0.05] (13/14,906)	Low	
		Operating time (min)	−5.81 [−7.68, −3.93] (16/15,480)	Low	
		Wound infection	0.96 [0.86, 1.07] (13/15,430)	High	
		Additional analgesia after 24–48 h	0.94 [0.79, 1.12] (1/9675)	High	
		Infectious morbidity	0.92 [0.72, 1.16] (11/14,985)	Moderate	
		Endometritis	1.07 [0.78, 1.46] (5/10,538)	Moderate	
		Blood transfusion >1 unit	0.98 [0.69, 1.39] (1/9675)	Moderate	
		Intervention for postpartum haemorrhage	0.99 [0.72, 1.38] (1/9675)	Moderate	
		Pain at 6 weeks postpartum	1.04 [0.80, 1.36] (1/9465)	Moderate	
		Hospital readmission	1.00 [0.67, 1.49] (1/9465)	Moderate	
		Maternal mortality	1.49 [0.25, 8.92] (1/9675)	Moderate	
		Adhesions	0.99 [0.76, 1.29] (4/282)	Low	
		Secondary infertility	0.89 [0.23, 3.44] (1/144)	Very low	
		Numbers of narcotic analgesics required	−0.18 [−0.39, 0.02] (1/1657)	Very Low	
		Uterine dehiscence	0.14 [0.01, 2.70] (1/100)	Very Low	

CI, Confidence Interval; RCT, Randomised controlled trial.

**Table 7:** Abdominal wall closure in caesarean section: layers other than the peritoneum.

Procedure	Control	Outcome	Effect estimate [CI] (No. RTC/No. participants)	Certainty of evidence	Category
**Techniquesand materials**					
**Fascia and muscle layers closure**					
**Subcutaneous closure**^58^^,^^59^					
Subcutaneous tissue closure	Non-closure	Seroma formation	0.53 [0.33, 0.84] (8/1979)	High	
		Any type of wound complications	0.66 [0.47, 0.93] (10/3811)	High	
		Wound infection	1.02 [0.69, 1.50] (5/1348)	Moderate	
		Endometritis	0.77 [0.46, 1.28] (1/590)	Moderate	
		Blood loss (mL)	9.00 [−24.29, 42.29] (1/590)	Moderate	
		Haematoma formation	0.74 [0.22, 2.42] (7/1663)	Moderate	
		Duration of surgery (min)	0.60 [−2.29, 3.49] (1/590)	Moderate	
**Skin closure**^60^, ^61^, ^62^, ^63^, ^64^					
Subcuticular PGA absorbable suture	Interrupted nylon suture	Hypertrophic scar at 6 months	1.85 [1.33, 2.58] (1/65)	Low	
Subcuticular barbed suture	Subcuticular PDS suture	Wound infection	0.96 [0.18, 5.10] (1/188)	Low	
		Wound complication	1.44 [0.30, 6.93] (1/188)	Low	
		Time skin closure (min)	0.60 [−0.30, 1.50] (1/188)	Low	
	Conventional sutures	Combined postoperative morbidity	0.88 [0.46, 1.65] (1/188)	Very Low	
Absorbable suture	Non absorbable metal staples	Wound separation	0.43 [0.32, 0.58] (11/2592)	Moderate	
		Wound infection	0.93 [0.47, 1.85] (14/3530)	Low	
		Hematoma	1.52 [0.66, 3.50] (7/1402)	Low	
		Seroma	1.01 [0.44, 2.35] (5/1188)	Very Low	
		Readmission for wound concerns	1.08 [0.49, 2.40] (3/1342)	Very Low	
Staples	Absorbable subcuticular suture	Hospital stay (days)	0.10 [−0.01, 0.21] (1/416)	Moderate	
		Skin separation	3.82 [2.05, 7.12] (5/824)	Moderate	
		Wound complications	1.52 [0.92, 2.52] (6/916)	Low	
		Seroma	0.32 [0.01, 7.68] (2/150)	Low	
		Pain at discharge (10 cm scale)	0.57 [−1.20, 2.33] (2/148)	Low	
		Pain at 6 weeks postpartum (10 cm scale)	0.59 [−1.17, 2.36] (2/145)	Low	
		Cosmesis per physician at 6 months (OSAS)	1.69 [−0.44, 3.83] (2/228)	Low	
		Patient satisfaction at discharge (10 cm scale)	−0.80 [−1.85, 0.25] (1/98)	Low	
		Total operative time (min)	−5.74 [−12.49, 1.02] (2/226)	Low	
		Reclosure	4.98 [1.82, 13.61] (2/516)	Very low	
		Wound infection	0.85 [0.43, 1.71] (6/916)	Very low	
		Hematoma	1.32 [0.10, 18.39] (3/283)	Very low	
		Readmission	0.56 [0.05, 6.08] (1/416)	Very low	
		Cosmesis per physician at 2 months (OSAS)	0.0 [−2.76, 2.76] (1/125)	Very low	
		Cosmesis per patient at 2 months (PSAS)	0.20 [−2.75, 3.15] (1/125)	Very low	
		Cosmesis per patient at 6 months (PSAS)	0.75 [−2.08, 3.59] (2/226)	Very low	
		Patient satisfaction 6–8 weeks (10 cm scale)	0.12 [−1.24, 1.49] (2/217)	Very low	
		Patient satisfaction at 6 months (10 cm scale)	−0.5 [−1.17, 0.17] (1/95)	Very low	
		Hypertrophic scar at 6 months	0.99 [0.58, 1.70] (1/95)	Very low	
Subcuticular suture: monofilament suture (poliglecaprone or polypropylene)	Subcuticular suture: multifilament suture (polyglactin)	SSI	0.71 [0.52, 0.98] (4/1845)	Low	
		Hematoma	0.70 [0.33, 1.45] (4/1845)	Very Low	
		Seroma	0.79 [0.42, 1.51] (3/1604)	Low	
		Wound dehiscence	0.94 [0.65, 1.37] (3/1641)	Low	
Staples (Pfannenstiel only)	Absorbable subcuticular suture (Pfannenstiel only)	Wound infection	0.41 [0.12, 1.36] (5/500)	Very Low	
		Wound complications	0.44 [0.14, 1.37] (5/500)	Very Low	
Clips	Sutures	Skin closure time (min)	−5.35 [−6.75, −3.95] (8/1728)	Moderate	
		Wound separation	2.33 [1.31, 4.12] (9/2644)	Moderate	
		Wound infection	1.12 [0.56, 2.25] (9/2605)	Low	
		Wound haematoma	2.46 [0.56, 10.75] (4/1364)	Low	
		Wound seroma	1.17 [0.48, 2.83] (4/1364)	Low	
		Patient scar assessment scale (PSAS)	0.44 [−2.10, 2.99] (7/1457)	Low	
		Observer scar assessment scale (OSAS)	0.32 [−0.75, 1.40] (7/1457)	Low	
		Maternal re-admission	1.28 [0.32, 5.02] (3/1462)	Very low	
		Hospital stay (days)	1.21 [0.14, 2.29] (5/1636)	Very low	
**All layers**^59^					
Blunt needles	Sharp needles	Wound infection	2.73 [0.54, 13.76] (1/203)	Low	

CI, Confidence interval; OSAS, observer scar assessment scale; PGA, Polyglycolic acid; PDS, polydioxanone suture; PSAS, patient scar assessment scale; RCT, Randomized controlled trial.

**Table 8:** Abdominal wall closure in caesarean section: wound drain and dressing related procedures.

Procedure	Control	Outcome	Effect estimate [CI] (No. RTC/No. participants)	Certainty of evidence	Category
**Drainage**^65^					
Wound drain	No drain	Postoperative analgesia	0.96 [0.87, 1.07] (1/2796)	High	
		Breastfeeding at hospital discharge	0.98 [0.92, 1.04] (1/2796)	High	
		Febrile morbidity	0.87 [0.66, 1.15] (6/3829)	Low	
		Endometritis	1.20 [0.9, 1.59] (2/3386)	Moderate	
		Wound complication	0.85 [0.55, 1.32] (6/1640)	Low	
		Blood loss (mL)	23.41 [−1.93, 48.74] (2/1030)	Low	
		Blood transfusion	1.02 [0.70, 1.48] (1/2796)	Moderate	
		Readmission	1.08 [0.70, 1.66] (2/3064)	Moderate	
		Operative procedures on wound	2.40 [0.85, 6.79] (1/2796)	Low	
		Postoperative pain	−0.15 [−0.36, 0.06] (1/148)	Very low	
Wound drain	Subcutaneous suture	Endometritis	1.31 [0.74, 2.34] (1/385)	Low	
		Wound infection	0.77 [0.42, 1.44] (3/533)	Very low	
		Wound complications	0.56 [0.17, 1.87] (3/533)	Very low	
		Blood loss (mL)	3.00 [−36.97, 42.97] (1/385)	Very low	
		Postoperative pain	−0.10 [−0.36, 0.16] (1/98)	Very low	
		Duration of surgery (min)	0.30 [−3.19, 3.79] (1/385)	Very low	
		Postnatal hospital stay (days)	0 [−0.3, 0.3] (1/385)	Very low	
Subcutaneous drain	Sub-sheath drain	Wound infection	5.42 [1.28, 22.98] (1/121)	Low	
		Febrile morbidity	1.28 [0.70, 2.34] (1/121)	Very Low	
**Wound healing**^66^, ^67^, ^68^					
Advanced dressing	Simple dressing	Wound dehiscence	0.51 [0.19, 1.34] (4/1496)	Low	
		SSI	0.81 [0.52, 1.24] (6/2295)	Very low	
		Endometritis	1.43 [0.09, 23.92] (3/1134)	Very low	
		Readmission	0.70 [0.24, 2.07] (5/1638)	Very low	
Advanced dressing: DACC-impregnated	Simple dressing	SSI	0.33 [0.14, 0.77] (2/685)	Low	
		Wound dehiscence	0.43 [0.06, 2.88] (4/1496)	Very low	
Advanced dressing: Application of silver-impregnated dressings	Simple dressing	SSI and superficial SSI	1.20 [0.77, 1.88] (2/1132)	Very low	
Basic wound contact dressings	Silver dressings	SSI	0.83 [0.51–1.37) (5/1353)	Very Low	
Negative pressure wound therapy	Standard dressing	Surgical site infection	0.78 [0.65, 0.95] (9/5529)	High	
		SSI (superficial)	0.70 [0.53, 0.92] (22/5539)	Moderate	
		Dehiscence	1.01 [0.82, 1.24] (6/5113)	High	
		SSI (deep)	0.95 [0.76, 1.18] (22/8521)	Moderate	
		Skin blisters	3.55 [1.43, 8.77] (11/5015)	Low	
		Haematoma	0.79 [0.48, 1.30] (17/5909)	Low	
		Reoperation	1.13 [0.91, 1.41] (18/6272)	Low	
		Maternal mortality	0.78 [0.47, 1.30] (11/6384)	Low	
		Pain	1.52 [0.20, 11.31] (2/632)	Very low	
		Seroma	0.82 [0.65, 1.05] (15/5436)	Very low	
		Readmission	0.98 [0.70, 1.38] (15/5853)	Very low	

SSI, Surgical site infection; DACC, Dialkylcarbamoyl chloride.

### Abdominal wall opening and related procedures

#### Preparation

There is evidence that cleaning the skin with chlorhexidine gluconate, compared with povidone iodine, probably reduces surgical site infections (SSI) (Table 2). Skin cleansing with chlorhexidine plus alcohol, compared with povidone iodine plus alcohol, may reduce SSI. For all other comparisons and outcomes on skin cleaning, the evidence is insufficient or uncertain. Also, there is insufficient or uncertain evidence on the effects of use versus non-use of adhesive drapes on SSI, metritis and length of stay.

#### Incision

There is no systematic review comparing the benefits or harms of different techniques and materials for opening each individual layer at CS (skin, subcutaneous tissue, fascia, rectus muscle, or peritoneum) (Table 3). Compared with the Pfannenstiel incision, the Joel-Cohen-based incision reduces fever and blood loss, and probably reduces time to oral intake, postoperative stay, operating time and skin incision to delivery time. Similarly, compared with lower midline (vertical) incision, the Joel-Cohen based (modified Misgav-Ladach) technique probably reduces blood loss, operating time, time to mobilization, and postoperative hospital stay. There is evidence that extraperitoneal, compared with intraperitoneal, caesarean reduces fever, and probably reduces serious complications. There is uncertain evidence on the effect of the type of scalpel used on wound infection and dehiscence. For all other comparisons for abdominal wall incision shown in Table 3, there is insufficient or uncertain evidence.

### Intraabdominal procedures

#### Preparation

Using the O'ring retractor probably reduces the need for uterine exteriorization and increases adequate visualization (Table 4). There are no SRs assessing the use of aspiration devices or surgical swabs during CS. It is uncertain whether forming a bladder flap at CS has any effect on relevant outcomes.

#### Uterine incision

There are no SRs comparing vertical versus transverse hysterotomy (Table 4). Opening the uterus using blunt instead of sharp dissection/expansion reduces operative time, probably reduces blood loss, and may reduce the need for blood transfusion. The use of cephalad-caudal, compared with transversal expansion, probably reduces unintended incision extension and blood loss. For all other uterine incision-related comparisons and outcomes, including materials, shown in Table 4, there is insufficient or uncertain evidence.

#### Foetal and placenta extraction and other intrauterine procedures

There are no SRs comparing different techniques for regular foetal extraction (Table 4). For difficult extractions of a cephalic foetus, the use of the reverse breech manoeuvre, compared to head push, may reduce uterine incision extension. The use of a fetal pillow in the case of impacted head may reduce operative time and incision-to-delivery interval. Manual placenta extraction, compared with spontaneous delivery/cord traction, increases endometritis, haemorrhage, and post-operative hospital stay, and has no effect on blood transfusion. In women undergoing pre-labour CS, manual cervical dilatation reduces blood loss and probably reduces retained products of conception. There is clear evidence that changing gloves at any time or after delivery of the placenta reduces SSI, while changing gloves at any time does not change the risk of endometritis. We did not identify any SRs on the effects of uterine cleansing or the timing of umbilical cord clamping. For all other comparisons and outcomes in Table 4, the evidence is insufficient or uncertain.

#### Uterine closure

Single-layer, compared with double-layer, uterine closure probably reduces CS duration, has no significant effects on postoperative febrile morbidity or wound infection, but increases dysmenorrhea (Table 5). Uterine exteriorization, compared with intraabdominal uterine repair, may reduce febrile morbidity but increases intraoperative nausea and vomiting (composite outcome), postoperative pain, the need for rescue analgesia and time to return to bowel function, and may have no effect on maternal satisfaction with the operation. There is clear evidence of benefit in favour of chromic catgut for uterine closure compared with multifilament sutures (mainly polygactin-910) in reducing the need for blood transfusion and relaparotomy, and possibly in reducing postoperative febrile morbidity. Barbed sutures, compared with non-barbed (conventional) sutures, probably reduce uterine repair time, operating time, and may reduce the need for additional haemostatic sutures.

#### Other intraabdominal procedures

Abdominal irrigation with an antibacterial compared with non-antibacterial solution may reduce SSI and hospital stay (Table 5). Irrigation with Icodextrin, compared with a lactated Ringer's solution, has no impact on adverse events. Standard, compared with pulsatile irrigation, may reduce SSI. However, abdominal irrigation with warm saline, compared with no treatment, increases intraoperative nausea and emesis and postoperative use of anti-emetics. For all other comparisons and outcomes in Table 5, the evidence is insufficient or uncertain.

### Abdominal wall closure

#### Peritoneum closure

Non-closure of parietal peritoneum only, compared with closure of both layers, probably reduces postoperative pain, and time to mobilization and to oral intake (Table 6). Non-closure of visceral peritoneum only, compared with closure of both layers, reduces urinary frequency, urgency of urination and stress incontinence, and may reduce operating time, and postoperative hospital stay. Non-closure versus closure of both layers probably reduces operating time, chronic pelvic pain, and postoperative hospital stay, but it does not affect the risk of wound infection or need for additional analgesia after 24–48 h. For all other comparisons and outcomes in Table 6, the evidence is insufficient or uncertain.

#### Closure of other layers

There are no SRs assessing benefits and harms of fascia and rectus muscle closure or approximation (Table 7). Closure, compared with non-closure, of subcutaneous tissue reduces seroma formation and wound complications.

#### Skin closure

For subcuticular suture, PGA absorbable suture, compared with interrupted nylon suture, may increase the risk of forming a hypertrophic scar and monofilament suture, compared with multifilament suture, may reduce surgical site infection (Table 7). Skin closure using absorbable sutures, compared with non-absorbable metal staples, probably reduces the risk of wound separation. The use of staples, compared with absorbable subcuticular sutures, probably has no effect on the length of the maternal stay but probably increases the risk of skin separation. The use of clips, compared to sutures, probably decreases skin closure time but probably increases the risk of wound separation. For all other comparisons and outcomes in Tables 6 and 7, the evidence is insufficient or uncertain.

#### Wound drain and dressing

Using versus not using a wound drain does not affect need for postoperative analgesia or breastfeeding at hospital discharge (Table 8). The use of a subcutaneous, compared to a sub-sheath, drain may increase the risk of wound infection. The use of advanced DACC-impregnated dressing, compared with simple dressing, may reduce SSI. Negative pressure wound therapy, compared with standard dressing, reduces SSI and probably reduces superficial SSI, but has no effect on the risk of dehiscence or deep SSI, and may increase skin blisters. For all other comparisons and outcomes in Table 8, the evidence is insufficient or uncertain.

## Discussion

This overview synthesizes the effects of procedures related to the surgical aspects of a caesarean birth. Sixteen Cochrane reviews and 22 non-Cochrane reviews were included, encompassing 628 RCTs and 190,349 participants. Using a pre-specified list of procedures and outcomes, we classified each procedure-outcome pair into pre-defined categories according to its effectiveness and certainty of the evidence (Box 1). These categories can inform clinicians and decision-makers to adopt CS procedures that can optimize specific outcomes, reconsider the use of procedures with no difference of effects, and stop using procedures with clear evidence of harm. The overview also identified procedures which are currently used in many facilities worldwide, but that require more research in order to assess their effectiveness. The results of this overview can inform the use of interventions in practice and guide decision-makers and policy-makers on resource allocation decisions.

An important concern emerging from this overview is the lack of evidence from SRs for several clinically relevant outcomes. For instance, for serious outcomes reported in the SRs (maternal or neonatal mortality, admission to the intensive care unit, any serious complication, sepsis, blood transfusion, re-operation), results are mostly inconclusive due to insufficient evidence. This may be because these were not the primary outcomes of the trials included in the SRs and thus the sample size was not calculated for these outcomes. Nevertheless, research is needed to fill this gap. An additional concern identified by this overview is the poor methodological quality of the available SRs on this important topic: only about 20% of the include reviews were of high or moderate quality according to the AMSTAR 2 tool.

This is the first overview of reviews on procedures related to the surgical aspects of a CS. We strived to adhere to rigorous methodological standards, including registration of the protocol with pre-specified procedures and outcomes before starting the overview, we designed a sensitive search strategy that was run in several electronic databases without date or language restrictions, we included Cochrane and non-Cochrane reviews, and conducted selection, data extraction procedures and quality assessment in duplicate. We also used pre-specified rules in case of duplicate reviews and comparisons to avoid double inclusion of studies. Providing the effectiveness of each procedure for each outcome independently allows for more tailored and locally meaningful decision-making. For example, in a setting where infections and sepsis represent the highest burden in morbidity and mortality (instead of postpartum haemorrhage), clinicians and hospital administrators may choose to implement a procedure that reduces infections even if it does not have any effect on haemorrhage.

A limitation of the overview is that we could not present results according to the type of CS since most SRs included trials that recruited a mix of women undergoing elective and emergency CS, and presented the pooled estimates of these trials without subgroup analyses. Similarly, due to the lack of information in the SRs, we could not assess differential effects of the interventions according to the number of previous CS, gestational age, indications, or maternal health status. An additional factor that can affect the average treatment effects of any surgical procedure is the experience and training of the surgeon, which was not reported in the included SRs. Lastly, we did not search for grey literature and, therefore, we may have missed additional relevant SRs.

As any surgery, a CS involves many steps and procedures that can vary substantially according to patient characteristics, surgeons' preferences and the availability of material and equipment. The lack of an internationally accepted standard technique for CS hinders the rigorous interpretation of the results of this overview. Even if the specific step or procedure under study was controlled in each RCT, the other steps could vary, and the effects of these variations are unknown. In other words, the effect estimates reported in the review may not represent the true effect of each procedure under study. To address this limitation, Dahlke et al.^12^ have suggested the use of a standardized CS and Stark et al.^69^ the use of a standardized form to collect information on the different steps of the CS, so that the procedures used beyond the comparison under study can be recorded and utilized to better interpret comparisons by putting them in context. Some studies, as the CORONIS trial^5^ have developed collection forms that could be adapted to these needs of standardization.

Our results should inform discussion and encourage stakeholders to promote the development of international recommendations for evidence-based CS techniques, which are urgently needed. These recommendations should promote the use of procedures with clear evidence of benefit and discourage procedures with clear evidence of harm. Recognizing the substantial amount of inconclusive or insufficient evidence found by this overview, these recommendations should be based not only on intervention effects but also on values, resources, equity, acceptability, and feasibility criteria.^70^ Anaesthetic and medical procedures go hand-in-hand with surgical procedures in a caesarean section. Although the focus of this manuscript was on the surgical aspects of a CS, forthcoming manuscripts will be published on the other aspects. In addition, the population of this overview was women without uterine or placental anomalies, such as placenta accreta or previa, which are important and prevalent in certain populations and would require additional specific procedures. Evidence syntheses for these other cases are warranted.

The results of this overview indicate large evidence gaps, which should inform future research priorities. Many trials will be needed to address all of the existing unanswered questions about CS procedures identified in this overview. The sample sizes of these trials should be calculated to address the many clinically relevant outcomes for which there are currently no evidence, such as severe maternal morbidity and mortality, and long-term outcomes including complications in future pregnancies. Future studies should follow strict guidelines that take into consideration the practical and methodological challenges of surgical trials, including the experience of the surgeons.^71^, ^72^, ^73^ Cost-effectiveness analyses are also warranted for policy-makers and organizations. Finally, there is a need for a core outcome set and a standardization of data collection at the time of caesarean section.

Current evidence from SRs suggests that various surgical procedures used for CS can optimize outcomes while many other procedures commonly used worldwide are harmful or lack conclusive evidence to support their use. There is an urgent need for the development of international guidelines supporting a standardized, evidence-based, technique for CS.

## Contributors

CG, MC, VD, JP, EA and APB conceived and drafted the outline of the manuscript and wrote the first draft. MRT contributed substantially to the writing of the final version of the manuscript. EA and ST contributed to the revision of the final manuscript. CG, MC, VD, JP, EA and APB developed the data extraction forms and performed screening and data collection. CG, MC, VD, JP, APB and MRT developed the annexes, tables and figures. All authors read and approved the final manuscript.

## Data sharing statement

The authors confirm that the data supporting the findings of this study are available within the article and its Supplementary materials.

## Declaration of interests

All authors declare no competing interests.

## Appendix A Supplementary data

Suplementary Material 2

Supplementary Material 4

Suplementary Material 5
