# Supplementary material for: Evidence-based surgical procedures to optimize caesarean outcomes: an overview of systematic reviews
Source: eClinicalMedicine. 2024 May 19;72:102632. doi: 10.1016/j.eclinm.2024.102632 (PMC11134562; doi:10.1016/j.eclinm.2024.102632)
Supplement: Suplementary Material 2 [file mmc1.docx]

**ANNEX 2**

**OVERVIEW OF INTERVENTIONS FOR SAFE CAESAREAN SECTION**

**SEARCH SATRATEGY**

| *cesarean OR caesarean OR c-section OR "C section" OR "abdominal deliver*" OR "surgical deliver*" OR "Cesarean Section"[Mesh] OR C-section*[TW] OR Postcesarean*[TW] OR Postcaesarean*[TW]* |
| --- |
| ***AND*** |
| *(Systematic[ti] and review*[ti]) OR Systematic overview*[ti] OR Cochrane review*[ti] OR Systemic review*[ti] OR Scoping review[ti] OR scoping literature review [ti] OR mapping review [ti] OR Umbrella review [ti] OR Review of reviews [ti] OR overview of reviews [ti] OR meta-review[ti] OR meta-synthesis [ti] OR metasynthesis [ti] OR meta-ethnography [ti] OR integrative review [ti] OR integrated review [ti] OR integrative overview[ti] OR quantitative review[ti] OR quantitative synthesis [ti] OR research synthesis[ti] OR systematic literature search[ti] OR systematic literature research[ti] OR meta-analyses [ti] OR metaanalyses [ti] OR metaanalysis [ti] OR meta-analysis [ti] OR meta-analytic review [ti] OR meta-analytical review [ti] OR meta-analysis[pt] OR (search* [tiab] OR medline [tiab] OR pubmed [tiab] OR embase [tiab] OR Cochrane [tiab] OR scopus [tiab] or web of science [tiab] OR sources of information [tiab] OR data sources [tiab] OR following databases [tiab]) AND (study selection [tiab] OR selection criteria [tiab] OR eligibility criteria [tiab] OR inclusion criteria [tiab] OR exclusion criteria [tiab]) OR Systematic review [pt]* |
