# Supplementary material for: Evidence-based surgical procedures to optimize caesarean outcomes: an overview of systematic reviews
Source: eClinicalMedicine. 2024 May 19;72:102632. doi: 10.1016/j.eclinm.2024.102632 (PMC11134562; doi:10.1016/j.eclinm.2024.102632)
Supplement: Supplementary Material 3 [file mmc2.docx]

**ANNEX 3**

**REFERENCES FOR EXCLUDED SRs AND INDIVIDUAL REASON FOR EXCLUSION**

| **TITLE** | **AUTHORS** | **REASON FOR EXCLUSION** |
| --- | --- | --- |
| Comparison of the Joel-Cohen-based technique and the transverse Pfannenstiel for caesarean section for safety and effectiveness: A systematic review and meta-analysis. | Olyaeemanesh A; Bavandpour E; Mobinizadeh M; Ashrafinia M; Bavandpour M; Nouhi M | UNCERTAIN DATA INTEGRITY |
| Transversus abdominis plane block for postoperative analgesia after Caesarean delivery performed under spinal anaesthesia? A systematic review and meta-analysis. | Abdallah FW; Halpern SH; Margarido CB | STUDIES ALREADY INCLUDED IN CSR ORNCSR LATEST VERSION |
| Regional versus general anaesthesia for caesarean section. | Afolabi BB; Lesi FE; Merah NA | UPADTED/OLD VERSION |
| Techniques and materials for skin closure in caesarean section. | Alderdice F; McKenna D; Dornan J | UPADTED/OLD VERSION |
| Different classes of antibiotics given to women routinely for preventing infection at caesarean section. | Alfirevic Z; Gyte GM; Dou L | UPADTED/OLD VERSION |
| P6 stimulation for the prevention of nausea and vomiting associated with cesarean delivery under neuraxial anesthesia: a systematic review of randomized controlled trials. | Allen TK; Habib AS | NOT A SR (Guidelines, Checklist, Review of review, Descriptive analysis, Review of literature) |
| Efficacy of perioperative cefuroxime as a prophylactic antibiotic in women requiring caesarean section: A systematic review. | Alrammaal HH; Batchelor HK; Morris RK; Chong HP | NOT A SR (Guidelines, Checklist, Review of review, Descriptive analysis, Review of literature) |
| Local anaesthetics and regional anaesthesia for preventing chronic pain after surgery. | Andreae MH; Andreae DA | UPADTED/OLD VERSION |
| Timing of administration of prophylactic antibiotics for caesarean section: a systematic review and meta-analysis. | Baaqeel H; Baaqeel R | UPADTED/OLD VERSION |
| Prophylactic Dose of Oxytocin for Uterine Atony during Caesarean Delivery: A Systematic Review. | Baliuliene V; Vitartaite M; Rimaitis K | NOT A SR (Guidelines, Checklist, Review of review, Descriptive analysis, Review of literature) |
| Effect of Perioperative Active Body Surface Warming Systems on Analgesic and Clinical Outcomes: A Systematic Review and Meta-analysis of Randomized Controlled Trials. | Balki I; Khan JS; Staibano P; Duceppe E; Bessissow A; Sloan EN; Morley EE; Thompson AN; Devereaux B; Rojas C; Siddiqui N; Sessler DI; Devereaux PJ | SPECIFIC POPULATION ( Generla population, obese woman, general anaesthesia) |
| Caesarean section wound infiltration with local anaesthesia for postoperative pain relief - any benefit? | Bamigboye AA; Hofmeyr GJ | STUDIES ALREADY INCLUDED IN CSR ORNCSR LATEST VERSION |
| Closure versus non-closure of the peritoneum at caesarean section. | Bamigboye AA; Hofmeyr GJ | UPADTED/OLD VERSION |
| Non-closure of peritoneal surfaces at caesarean section--a systematic review. | Bamigboye AA; Hofmeyr GJ | UPADTED/OLD VERSION |
| Uterine externalization versus in situ repair of hysterotomy during cesarean delivery: a systematic review, equivalence meta-analysis, and trial sequential analysis. | Bhat A; Jaffer D; Keasler P; Kamath K; Kelly J; Singh PM | UPADTED/OLD VERSION |
| [Antimicrobial prophylaxis for caesarean delivery: before or after cord clamping? A meta-analysis]. | Boselli E; Bouvet L; RimmelÃ© T; Chassard D; Allaouchiche B | UPADTED/OLD VERSION |
| Intraoperative prophylactic and therapeutic non-invasive ventilation: a systematic review. | Cabrini L; Nobile L; Plumari VP; Landoni G; Borghi G; Mucchetti M; Zangrillo A | NOT OUTCOMES OF INTEREST |
| Vaginal Cleansing Before Cesarean Delivery: A Systematic Review and Meta-analysis. | Caissutti C; Saccone G; Zullo F; Quist-Nelson J; Felder L; Ciardulli A; Berghella V | STUDIES ALREADY INCLUDED IN CSR ORNCSR LATEST VERSION |
| Evidence-Based Bundles and Cesarean Delivery Surgical Site Infections: A Systematic Review and Meta-analysis. | Carter EB; Temming LA; Fowler S; Eppes C; Gross G; Srinivas SK; Macones GA; Colditz GA; Tuuli MG | NOT A SR (Guidelines, Checklist, Review of review, Descriptive analysis, Review of literature) |
| Guidelines for intraoperative care in cesarean delivery: Enhanced Recovery After Surgery Society Recommendations (Part 2). | Caughey AB; Wood SL; Macones GA; Wrench IJ; Huang J; Norman M; Pettersson K; Fawcett WJ; Shalabi MM; Metcalfe A; Gramlich L; Nelson G; Wilson RD | NOT A SR (Guidelines, Checklist, Review of review, Descriptive analysis, Review of literature) |
| Supplemental oxygen for caesarean section during regional anaesthesia. | Chatmongkolchart S; Prathep S | UPADTED/OLD VERSION |
| Suture closure of subcutaneous fat and wound disruption after cesarean delivery: a meta-analysis. | Chelmow D; Rodriguez EJ; Sabatini MM | UPADTED/OLD VERSION |
| To close or not to close? A systematic review and a meta-analysis of peritoneal non-closure and adhesion formation after caesarean section. | Cheong YC; Premkumar G; Metwally M; Peacock JL; Li TC | UPADTED/OLD VERSION |
| Techniques for preventing hypotension during spinal anaesthesia for caesarean section. | Chooi C; Cox JJ; Lumb RS; Middleton P; Chemali M; Emmett RS; Simmons SW; Cyna AM | UPADTED/OLD VERSION |
| Chewing gum improves postoperative recovery of gastrointestinal function after cesarean delivery: a systematic review and meta-analysis of randomized trials. | Ciardulli A; Saccone G; Di Mascio D; Caissutti C; Berghella V | STUDIES ALREADY INCLUDED IN CSR ORNCSR LATEST VERSION |
| Maternal position during caesarean section for preventing maternal and neonatal complications. | Cluver C; Novikova N; Hofmeyr GJ; Hall DR | UPADTED/OLD VERSION |
| Outcomes of second stage cesarean section following the use of a fetal head elevation device: A systematic review and meta-analysis. | Conde-Agudelo A; Nieto A; Rosas-Bermudez A; Romero R | UPADTED/OLD VERSION |
| A systematic review of the effects of adding neostigmine to local anesthetics for neuraxial administration in obstetric anesthesia and analgesia. | Cossu AP; De Giudici LM; Piras D; Mura P; Scanu M; Cossu M; Saba M; Finco G; Brazzi L | NOT OUTCOMES OF INTEREST |
| Chewing gum in preventing postoperative ileus in women undergoing caesarean section: a systematic review and meta-analysis of randomised controlled trials. | Craciunas L; Sajid MS; Ahmed AS | UPADTED/OLD VERSION |
| Intrathecal clonidine as an adjuvant for neuraxial anaesthesia during caesarean delivery: a systematic review and meta-analysis of randomised trials. | Crespo S; Dangelser G; Haller G | NOT OUTCOMES OF INTEREST |
| Techniques for preventing hypotension during spinal anaesthesia for caesarean section. | Cyna AM; Andrew M; Emmett RS; Middleton P; Simmons SW | UPADTED/OLD VERSION |
| Intraoperative and postoperative analgesic efficacy and adverse effects of intrathecal opioids in patients undergoing cesarean section with spinal anesthesia: a qualitative and quantitative systematic review of randomized controlled trials. | Dahl JB; Jeppesen IS; JÃ¸rgensen H; Wetterslev J; MÃ¸iniche S | UPADTED/OLD VERSION |
| Evidence-based surgery for cesarean delivery: an updated systematic review. | Dahlke JD; Mendez-Figueroa H; Rouse DJ; Berghella V; Baxter JK; Chauhan SP | NOT A SR (Guidelines, Checklist, Review of review, Descriptive analysis, Review of literature) |
| Preventing Hypothermia during Cesarean Birth: An Integrative Review. | Dendis M; Hooven K | NOT A SR (Guidelines, Checklist, Review of review, Descriptive analysis, Review of literature) |
| Outcomes of second stage cesarean section following the use of a fetal head elevation device: A systematic review and meta-analysis. | Di Girolamo R; Galliani C; Buca D; Liberati M; D'Antonio F | NOT A SR (Guidelines, Checklist, Review of review, Descriptive analysis, Review of literature) |
| The efficacy of abdominal binders in reducing postoperative pain and distress after cesarean delivery: A meta-analysis of randomized controlled trials. | Di Mascio D; Caruso G; Prata G; Saccone G; Terrin G; Giancotti A; Brunelli R; Muzii L; Benedetti Panici P; Di Donato V | STUDIES ALREADY INCLUDED IN CSR ORNCSR LATEST VERSION |
| Risk of Cesarean scar defect following single- vs double-layer uterine closure: systematic review and meta-analysis of randomized controlled trials. | Di Spiezio Sardo A; Saccone G; McCurdy R; Bujold E; Bifulco G; Berghella V | STUDIES ALREADY INCLUDED IN CSR ORNCSR LATEST VERSION |
| Surgical techniques for uterine incision and uterine closure at the time of caesarean section. | Dodd JM; Anderson ER; Gates S | UPADTED/OLD VERSION |
| Tocolysis for assisting delivery at caesarean section. | Dodd JM; Reid K | UPADTED/OLD VERSION |
| Techniques for preventing hypotension during spinal anaesthesia for caesarean section. | Emmett RS; Cyna AM; Andrew M; Simmons SW | UPADTED/OLD VERSION |
| Techniques for preventing hypotension during spinal anaesthesia for caesarean section. | Emmett RS; Cyna AM; Andrew M; Simmons SW | UPADTED/OLD VERSION |
| Single versus two layer suturing for closing the uterine incision at caesarean section. | Enkin MW; Wilkinson C | UPADTED/OLD VERSION |
| [Efficacy and safety of tranexamic acid administration for the prevention and/or the treatment of post-partum haemorrhage: a systematic review with meta-analysis]. | Faraoni D; Carlier C; Samama CM; Levy JH; Ducloy-Bouthors AS | UPADTED/OLD VERSION |
| Are prophylactic adjunctive macrolides efficacious against caesarean section surgical site infection: A systematic review and meta-analysis. | Farmer N; Hodgetts-Morton V; Morris RK | UPADTED/OLD VERSION |
| Epidemiology, prevention and management of early postpartum hemorrhage - a systematic review. | Feduniw S; Warzecha D; Szymusik I; Wielgos M | NOT A SR (Guidelines, Checklist, Review of review, Descriptive analysis, Review of literature) |
| Prevention of hypotension after spinal anaesthesia for caesarean section: a systematic review and network meta-analysis of randomised controlled trials. | Fitzgerald JP; Fedoruk KA; Jadin SM; Carvalho B; Halpern SH | NOT A SR (Guidelines, Checklist, Review of review, Descriptive analysis, Review of literature) |
| Safety and efficacy of tranexamic acid for prevention of obstetric haemorrhage: an updated systematic review and meta-analysis. | Franchini M; Mengoli C; Cruciani M; Bergamini V; Presti F; Marano G; Pupella S; Vaglio S; Masiello F; Veropalumbo E; Piccinini V; Pati I; Liumbruno GM | STUDIES ALREADY INCLUDED IN CSR ORNCSR LATEST VERSION |
| [Continuous infusion of local anesthetic at the site of the abdominal surgical wound for postoperative analgesia: a systematic review]. | Fustran Guerrero N; Dalmau LlitjÃ³s A; SabatÃ© Pes A | NOT A SR (Guidelines, Checklist, Review of review, Descriptive analysis, Review of literature) |
| Wound drainage for caesarean section. | Gates S; Anderson ER | UPADTED/OLD VERSION |
| Prophylaxis for venous thromboembolic disease in pregnancy and the early postnatal period. | Gates S; Brocklehurst P; Davis LJ | UPADTED/OLD VERSION |
| Effect of negative-pressure wound therapy on wound complications in obese women after caesarean birth: a systematic review and meta-analysis. | Gillespie BM; Thalib L; Ellwood D; Kang E; Mahomed K; Kumar S; Chaboyer W | SPECIFIC POPULATION ( Generla population, obese woman, general anaesthesia) |
| Effects of colloid preload on the incidence of hypotension in spinal anesthesia for cesarean section: a systematic review and meta-analysis. | Gong RS; Liu XW; Li WX; Zhao J | STUDIES ALREADY INCLUDED IN CSR ORNCSR LATEST VERSION |
| Interventions for preventing nausea and vomiting in women undergoing regional anaesthesia for caesarean section. | Griffiths JD; Gyte GM; Paranjothy S; Brown HC; Broughton HK; Thomas J | UPADTED/OLD VERSION |
| The safety of early postpartum discharge: a review and critique. | Grullon KE; Grimes DA | UPADTED/OLD VERSION |
| Prophylactic negative pressure wound therapy on surgical site infection in obese women after cesarean section: A systematic review and meta-analysis. | Guo C; Cheng T; Li J | SPECIFIC POPULATION ( Generla population, obese woman, general anaesthesia) |
| Different classes of antibiotics given to women routinely for preventing infection at caesarean section. | Gyte GM; Dou L; Vazquez JC | UPADTED/OLD VERSION |
| Vaginal preparation with antiseptic solution before cesarean section for preventing postoperative infections. | Haas DM; Morgan Al Darei S; Contreras K | UPADTED/OLD VERSION |
| Vaginal preparation with antiseptic solution before cesarean section for preventing postoperative infections. | Haas DM; Morgan S; Contreras K | UPADTED/OLD VERSION |
| Vaginal preparation with antiseptic solution before cesarean section for preventing postoperative infections. | Haas DM; Morgan S; Contreras K | UPADTED/OLD VERSION |
| Vaginal preparation with antiseptic solution before cesarean section for preventing postoperative infections. | Haas DM; Morgan S; Contreras K; Enders S | UPADTED/OLD VERSION |
| Skin preparation for preventing infection following caesarean section. | Hadiati DR; Hakimi M; Nurdiati DS | UPADTED/OLD VERSION |
| Skin preparation for preventing infection following caesarean section. | Hadiati DR; Hakimi M; Nurdiati DS; da Silva Lopes K; Ota E | UPADTED/OLD VERSION |
| Skin preparation for preventing infection following caesarean section. | Hadiati DR; Hakimi M; Nurdiati DS; Ota E | UPADTED/OLD VERSION |
| Staples versus subcuticular suture for cesarean skin closure in obese women: A systematic review and meta-analysis. | Han D; Feng L; Xu L; Li C; Zhang Q | SPECIFIC POPULATION ( Generla population, obese woman, general anaesthesia) |
| Intravenous ketamine during spinal and general anaesthesia for caesarean section: systematic review and meta-analysis. | Heesen M; BÃ¶hmer J; Brinck EC; Kontinen VK; KlÃ¶hr S; Rossaint R; Straube S | STUDIES ALREADY INCLUDED IN CSR ORNCSR LATEST VERSION |
| Is general anaesthesia for caesarean section associated with postpartum haemorrhage? Systematic review and meta-analysis. | Heesen M; Hofmann T; KlÃ¶hr S; Rossaint R; van de Velde M; Deprest J; Straube S | STUDIES ALREADY INCLUDED IN CSR ORNCSR LATEST VERSION |
| Prophylactic phenylephrine for caesarean section under spinal anaesthesia: systematic review and meta-analysis. | Heesen M; KÃ¶lhr S; Rossaint R; Straube S | NOT OUTCOMES OF INTEREST |
| Maternal and foetal effects of remifentanil for general anaesthesia in parturients undergoing caesarean section: a systematic review and meta-analysis. | Heesen M; KlÃ¶hr S; Hofmann T; Rossaint R; Devroe S; Straube S; Van de Velde M | SPECIFIC POPULATION ( Generla population, obese woman, general anaesthesia) |
| Concerning the timing of antibiotic administration in women undergoing caesarean section: a systematic review and meta-analysis. | Heesen M; KlÃ¶hr S; Rossaint R; Allegaert K; Allegeaert K; Deprest J; Van de Velde M; Straube S | UPADTED/OLD VERSION |
| Prevention of Spinal Anesthesia-Induced Hypotension During Cesarean Delivery by 5-Hydroxytryptamine-3 Receptor Antagonists: A Systematic Review and Meta-analysis and Meta-regression. | Heesen M; Klimek M; Hoeks SE; Rossaint R | STUDIES ALREADY INCLUDED IN CSR ORNCSR LATEST VERSION |
| Prophylactic subcutaneous drainage for prevention of wound complications after cesarean delivery--a metaanalysis. | Hellums EK; Lin MG; Ramsey PS | UPADTED/OLD VERSION |
| Hyperbaric versus plain bupivacaine for spinal anesthesia for cesarean delivery. | Heng Sia AT; Tan KH; Sng BL; Lim Y; Chan ESY; Siddiqui FJ | UPADTED/OLD VERSION |
| Gum chewing and gastrointestinal function following caesarean delivery: a systematic review and meta-analysis. | Hochner H; Tenfelde SM; Abu Ahmad W; Liebergall-Wischnitzer M | UPADTED/OLD VERSION |
| Antibiotic prophylaxis regimens and drugs for cesarean section. | Hopkins L; Smaill F | UPADTED/OLD VERSION |
| Information for pregnant women about caesarean birth. | Horey D; Weaver J; Russell H | NOT OUTCOMES OF INTEREST |
| Hypnotic agents for induction of general anesthesia in cesarean section patients: A systematic review and meta-analysis of randomized controlled trials. | Houthoff Khemlani K; Weibel S; Kranke P; Schreiber JU | SPECIFIC POPULATION ( Generla population, obese woman, general anaesthesia) |
| Effect of misoprostol versus oxytocin during caesarean section: a systematic review and meta-analysis. | Hua J; Chen G; Xing F; Scott M; Li Q | UPADTED/OLD VERSION |
| Usefulness of chewing gum for recovering intestinal function after cesarean delivery: A systematic review and meta-analysis of randomized controlled trials. | Huang HP; He M | UPADTED/OLD VERSION |
| Implementation of vaginal preparation prior to caesarean section. | Jakes AD; Bell A; Chiwera L; Lloyd J | NOT A SR (Guidelines, Checklist, Review of review, Descriptive analysis, Review of literature) |
| Carbetocin for the prevention of postpartum hemorrhage: a systematic review and meta-analysis of randomized controlled trials. | Jin B; Du Y; Zhang F; Zhang K; Wang L; Cui L | UPADTED/OLD VERSION |
| Single injection Quadratus Lumborum block for postoperative analgesia in adult surgical population: A systematic review and meta-analysis. | Jin Z; Liu J; Li R; Gan TJ; He Y; Lin J | STUDIES ALREADY INCLUDED IN CSR ORNCSR LATEST VERSION |
| [Early post-partum discharge : systematic review of the literature]. | Jonguitud-Aguilar A; Tomasso G; Cafferatta ML | NOT A SR (Guidelines, Checklist, Review of review, Descriptive analysis, Review of literature) |
| A scoping review of maternal antibiotic prophylaxis in low- and middle-income countries: Comparison to WHO recommendations for prevention and treatment of maternal peripartum infection. | Jury I; Thompson K; Hirst JE | NOT A SR (Guidelines, Checklist, Review of review, Descriptive analysis, Review of literature) |
| Efficacy of carbetocin in the prevention of postpartum hemorrhage: a systematic review and Bayesian meta-analysis of randomized trials. | Kalafat E; Gokce A; O'Brien P; Benlioglu C; Koc A; Karaaslan O; Khalil A | UPADTED/OLD VERSION |
| Does tranexamic acid prevent postpartum haemorrhage? A systematic review of randomised controlled trials. | Ker K; Shakur H; Roberts I | UPADTED/OLD VERSION |
| Combined spinal-epidural vs. spinal anaesthesia for caesarean section: meta-analysis and trial-sequential analysis. | Klimek M; Rossaint R; van de Velde M; Heesen M | UPADTED/OLD VERSION |
| A meta-analysis of the effect of inspired oxygen concentration on the incidence of surgical site infection following cesarean section. | Klingel ML; Patel SV | NOT OUTCOMES OF INTEREST |
| Evidence-based value of subcutaneous surgical wound drainage: the largest systematic review and meta-analysis. | Kosins AM; Scholz T; Cetinkaya M; Evans GRD | STUDIES ALREADY INCLUDED IN CSR ORNCSR LATEST VERSION |
| A systematic review of the cost-effectiveness of uterotonic agents for the prevention of postpartum hemorrhage. | Lawrie TA; RogoziÅ„ska E; Sobiesuo P; Vogel JP; Ternent L; Oladapo OT | NOT OUTCOMES OF INTEREST |
| A dose-response meta-analysis of prophylactic intravenous ephedrine for the prevention of hypotension during spinal anesthesia for elective cesarean delivery. | Lee A; Ngan Kee WD; Gin T | STUDIES ALREADY INCLUDED IN CSR ORNCSR LATEST VERSION |
| A quantitative, systematic review of randomized controlled trials of ephedrine versus phenylephrine for the management of hypotension during spinal anesthesia for cesarean delivery. | Lee A; Ngan Kee WD; Gin T | STUDIES ALREADY INCLUDED IN CSR ORNCSR LATEST VERSION |
| Prophylactic ephedrine prevents hypotension during spinal anesthesia for Cesarean delivery but does not improve neonatal outcome: a quantitative systematic review. | Lee A; Ngan Kee WD; Gin T | STUDIES ALREADY INCLUDED IN CSR ORNCSR LATEST VERSION |
| Effect of intravenous dexmedetomidine and remifentanil on neonatal outcomes after caesarean section under general anaesthesia: A systematic review and meta-analysis. | Lee M; Kim H; Lee C; Kang H | SPECIFIC POPULATION ( Generla population, obese woman, general anaesthesia) |
| Is prophylactic tranexamic acid administration effective and safe for postpartum hemorrhage prevention?: A systematic review and meta-analysis. | Li C; Gong Y; Dong L; Xie B; Dai Z | STUDIES ALREADY INCLUDED IN CSR ORNCSR LATEST VERSION |
| Is routine indwelling catheterisation of the bladder for caesarean section necessary? A systematic review. | Li L; Wen J; Wang L; Li YP; Li Y | STUDIES ALREADY INCLUDED IN CSR ORNCSR LATEST VERSION |
| Comparing prophylactic use of cefazolin for SSI in cesarean section: a systematic review and meta-analysis. | Li M; Shi B; Ma J; Peng X; Shi J | STUDIES ALREADY INCLUDED IN CSR ORNCSR LATEST VERSION |
| Subarachnoid and epidural dexmedetomidine for the prevention of post-anesthetic shivering: a meta-analysis and systematic review. | Li YZ; Jiang Y; Lin H; Yang XP | SPECIFIC POPULATION ( Generla population, obese woman, general anaesthesia) |
| Mechanical dilatation of the cervix at non-labour caesarean section for reducing postoperative morbidity. | Liabsuetrakul T; Peeyananjarassri K | UPADTED/OLD VERSION |
| Effect of Intravenous Ketamine on Hypocranial Pressure Symptoms in Patients with Spinal Anesthetic Cesarean Sections: A Systematic Review and Meta-Analysis. | Liang X; Yang X; Liang S; Zhang Y; Ding Z; Guo Q; Huang C | NOT OUTCOMES OF INTEREST |
| Shivering prevention and treatment during cesarean delivery under neuraxial anesthesia: a systematic review. | Liu J; Wang Y; Ma W | NOT A SR (Guidelines, Checklist, Review of review, Descriptive analysis, Review of literature) |
| Intraoperative interventions for preventing surgical site infection: an overview of Cochrane Reviews. | Liu Z; Dumville JC; Norman G; Westby MJ; Blazeby J; McFarlane E; Welton NJ; O'Connor L; Cawthorne J; George RP; Crosbie EJ; Rithalia AD; Cheng HY | NOT A SR (Guidelines, Checklist, Review of review, Descriptive analysis, Review of literature) |
| Guidelines for postoperative care in cesarean delivery: Enhanced Recovery After Surgery (ERAS) Society recommendations (part 3). | Macones GA; Caughey AB; Wood SL; Wrench IJ; Huang J; Norman M; Pettersson K; Fawcett WJ; Shalabi MM; Metcalfe A; Gramlich L; Nelson G; Wilson RD | NOT A SR (Guidelines, Checklist, Review of review, Descriptive analysis, Review of literature) |
| Preincision adjunctive prophylaxis for cesarean deliveries a systematic review and meta-analysis. | Markwei MT; Babatunde I; Rathi N; Fan C; Prah MA; Joo J; Hackett L; Soper DE; Goje O | STUDIES ALREADY INCLUDED IN CSR ORNCSR LATEST VERSION |
| Best practice perioperative strategies and surgical techniques for preventing caesarean section surgical site infections: a systematic review of reviews and meta-analyses. | Martin EK; Beckmann MM; Barnsbee LN; Halton KA; Merollini K; Graves N | NOT A SR (Guidelines, Checklist, Review of review, Descriptive analysis, Review of literature) |
| Abdominal surgical incisions for caesarean section. | Mathai M; Hofmeyr GJ | UPADTED/OLD VERSION |
| The association of skin incision placement during cesarean delivery with wound complications in obese women: a systematic review and meta-analysis. | Mccurdy RJ; Felder LA; Saccone G; Edwards RK; Thornburg LL; Marrs C; Conner SN; Strauss R; Berghella V | SPECIFIC POPULATION ( Generla population, obese woman, general anaesthesia) |
| Intravenous Magnesium Sulphate for Analgesia after Caesarean Section: A Systematic Review. | McKeown A; Seppi V; Hodgson R | NOT A SR (Guidelines, Checklist, Review of review, Descriptive analysis, Review of literature) |
| Practices to Reduce Surgical Site Infections Among Women Undergoing Cesarean Section: A Review. | McKibben RA; Pitts SI; Suarez-Cuervo C; Perl TM; Bass EB | NOT A SR (Guidelines, Checklist, Review of review, Descriptive analysis, Review of literature) |
| The Clinical Efficacy and Safety of Enhanced Recovery After Surgery for Cesarean Section: A Systematic Review and Meta-Analysis of Randomized Controlled Trials and Observational Studies. | Meng X; Chen K; Yang C; Li H; Wang X | NOT A SR (Guidelines, Checklist, Review of review, Descriptive analysis, Review of literature) |
| Transversus abdominis plane block for analgesia after Cesarean delivery: a systematic review and meta-analysis. | Mishriky BM; George RB; Habib AS | STUDIES ALREADY INCLUDED IN CSR ORNCSR LATEST VERSION |
| Metoclopramide for nausea and vomiting prophylaxis during and after Caesarean delivery: a systematic review and meta-analysis. | Mishriky BM; Habib AS | UPADTED/OLD VERSION |
| Effectiveness of strategies for the management and/or prevention of hypothermia within the adult perioperative environment. | Moola S; Lockwood C | NOT OUTCOMES OF INTEREST |
| The effects of an increase of central blood volume before spinal anesthesia for cesarean delivery: a qualitative systematic review. | Morgan PJ; Halpern SH; Tarshis J | NOT A SR (Guidelines, Checklist, Review of review, Descriptive analysis, Review of literature) |
| Codeine-acetaminophen versus nonsteroidal anti-inflammatory drugs in the treatment of post-abdominal surgery pain: a systematic review of randomized trials. | Nauta M; Landsmeer ML; Koren G | SPECIFIC POPULATION ( Generla population, obese woman, general anaesthesia) |
| Negative pressure wound therapy for surgical wounds healing by primary closure. | Norman G; Goh EL; Dumville JC; Shi C; Liu Z; Chiverton L; Stankiewicz M; Reid A | UPADTED/OLD VERSION |
| Negative pressure wound therapy for surgical wounds healing by primary closure. | Norman G; Goh EL; Dumville JC; Shi C; Liu Z; Chiverton L; Stankiewicz M; Reid A | UPADTED/OLD VERSION |
| Tranexamic acid for preventing postpartum haemorrhage. | Novikova N; Hofmeyr GJ | UPADTED/OLD VERSION |
| Effects of perioperative oxygen concentration on oxidative stress in adult surgical patients: a systematic review. | Oldman AH; Martin DS; Feelisch M; Grocott MPW; Cumpstey AF | SPECIFIC POPULATION ( Generla population, obese woman, general anaesthesia) |
| Carbetocin compared with oxytocin in non-elective Cesarean delivery: a systematic review, meta-analysis, and trial sequential analysis of randomized-controlled trials. | Onwochei DN; Owolabi A; Singh PM; Monks DT | UPADTED/OLD VERSION |
| Carbetocin reduces the need for additional uterotonics in elective caesarean delivery: a systematic review, meta-analysis and trial sequential analysis of randomised controlled trials. | Onwochei DN; Van Ross J; Singh PM; Salter A; Monks DT | UPADTED/OLD VERSION |
| Retrospective analysis on the efficacy of corticosteroid prophylaxis prior to elective caesarean section to reduce neonatal respiratory complications at term of pregnancy: review of literature. | Paganelli S; Soncini E; Gargano G; Capodanno F; Vezzani C; La Sala GB | NOT A SR (Guidelines, Checklist, Review of review, Descriptive analysis, Review of literature) |
| Interventions at caesarean section for reducing the risk of aspiration pneumonitis. | Paranjothy S; Griffiths JD; Broughton HK; Gyte GM; Brown HC; Thomas J | UPADTED/OLD VERSION |
| Interventions at caesarean section for reducing the risk of aspiration pneumonitis. | Paranjothy S; Griffiths JD; Broughton HK; Gyte GM; Brown HC; Thomas J | UPADTED/OLD VERSION |
| The Effect of Glycopyrrolate on the Incidence of Hypotension and Vasopressor Requirement During Spinal Anesthesia for Cesarean Delivery: A Meta-analysis. | Patel SD; Habib AS; Phillips S; Carvalho B; Sultan P | STUDIES ALREADY INCLUDED IN CSR ORNCSR LATEST VERSION |
| Prevention of Surgical Site Infections: A Systematic Review of Cost Analyses in the Use of Prophylactic Antibiotics. | Purba AKR; Setiawan D; Bathoorn E; Postma MJ; Dik JH; Friedrich AW | NOT A SR (Guidelines, Checklist, Review of review, Descriptive analysis, Review of literature) |
| Postoperative Discharge Opioid Consumption, Leftover, and Disposal after Obstetric and Gynecologic Procedures: A Systematic Review. | Raina J; Costello C; Suarthana E; Tulandi T | NOT OUTCOMES OF INTEREST |
| Autologous amniotic membrane: An accelerator of wound healing for prevention of surgical site infections following Cesarean delivery. | Rezazadeh D; Anvari Aliabad R; Norooznezhad AH | NOT A SR (Guidelines, Checklist, Review of review, Descriptive analysis, Review of literature) |
| Fluid loading therapy to prevent spinal hypotension in women undergoing elective caesarean section: Network meta-analysis, trial sequential analysis and meta-regression. | Rijs K; Mercier FJ; Lucas DN; Rossaint R; Klimek M; Heesen M | STUDIES ALREADY INCLUDED IN CSR ORNCSR LATEST VERSION |
| Analgesic efficacy of the ultrasound-guided blockade of the transversus abdominis plane - a systematic review. | RipollÃ©s J; Mezquita SM; Abad A; Calvo J | STUDIES ALREADY INCLUDED IN CSR ORNCSR LATEST VERSION |
| Colloids versus crystalloids in the prevention of hypotension induced by spinal anesthesia in elective cesarean section. A systematic review and meta-analysis. | RipollÃ©s Melchor J; Espinosa Ã; MartÃ­nez Hurtado E; Casans FrancÃ©s R; Navarro PÃ©rez R; Abad Gurumeta A; Calvo Vecino JM | STUDIES ALREADY INCLUDED IN CSR ORNCSR LATEST VERSION |
| Povidone-iodine 1% is the most effective vaginal antiseptic for preventing post-cesarean endometritis: aÂ systematic review and network meta-analysis. | Roeckner JT; Sanchez-Ramos L; Mitta M; Kovacs A; Kaunitz AM | UPADTED/OLD VERSION |
| PROSPECT guideline for elective caesarean section: updated systematic review and procedure-specific postoperative pain management recommendations. | Roofthooft E; Joshi GP; Rawal N; Van de Velde M | NOT A SR (Guidelines, Checklist, Review of review, Descriptive analysis, Review of literature) |
| A Systematic Review Evaluating Neuraxial Morphine and Diamorphine-Associated Respiratory Depression After Cesarean Delivery. | Sharawi N; Carvalho B; Habib AS; Blake L; Mhyre JM; Sultan P | NOT A SR (Guidelines, Checklist, Review of review, Descriptive analysis, Review of literature) |
| Evaluation of patient-reported outcome measures of functional recovery following caesarean section: a systematic review using the consensus-based standards for the selection of health measurement instruments (COSMIN) checklist. | Sharawi N; Klima L; Shah R; Blake L; Carvalho B; Sultan P | NOT A SR (Guidelines, Checklist, Review of review, Descriptive analysis, Review of literature) |
| Chewing gum for postoperative recovery of gastrointestinal function. | Short V; Herbert G; Perry R; Atkinson C; Ness AR; Penfold C; Thomas S; Andersen HK; Lewis SJ | UPADTED/OLD VERSION |
| Use of hyperbaric versus isobaric bupivacaine for spinal anaesthesia for caesarean section. | Sia AT; Tan KH; Sng BL; Lim Y; Chan ES; Siddiqui FJ | UPADTED/OLD VERSION |
| Tranexamic acid for preventing postpartum blood loss after cesarean delivery: a systematic review and meta-analysis of randomized controlled trials. | Simonazzi G; Bisulli M; Saccone G; Moro E; Marshall A; Berghella V | STUDIES ALREADY INCLUDED IN CSR ORNCSR LATEST VERSION |
| The analgesic effectiveness of ilioinguinal-iliohypogastric block for caesarean delivery: A meta-analysis and trial sequential analysis. | Singh NP; Makkar JK; Bhatia N; Singh PM | ARTICLE MISSING |
| The analgesic efficacy of quadratus lumborum block in caesarean delivery: a meta-analysis and trial sequential analysis. | Singh NP; Makkar JK; Borle A; Monks D; Goudra BG; Zorrilla-Vaca A; Singh PM | STUDIES ALREADY INCLUDED IN CSR ORNCSR LATEST VERSION |
| Antibiotic prophylaxis for cesarean section. | Smaill F; Hofmeyr GJ | UPADTED/OLD VERSION |
| Antibiotic prophylaxis for cesarean section. | Smaill F; Hofmeyr GJ | UPADTED/OLD VERSION |
| Antibiotic prophylaxis versus no prophylaxis for preventing infection after cesarean section. | Smaill FM; Gyte GM | UPADTED/OLD VERSION |
| Hyperbaric vs. isobaric bupivacaine for spinal anaesthesia for elective caesarean section: a Cochrane systematic review. | Sng BL; Han NLR; Leong WL; Sultana R; Siddiqui FJ; Assam PN; Chan ES; Tan KH; Sia AT | STUDIES ALREADY INCLUDED IN CSR ORNCSR LATEST VERSION |
| Single dose perioperative intrathecal ketamine as an adjuvant to intrathecal bupivacaine: A systematic review and meta-analysis of adult human randomized controlled trials. | Sohnen S; Dowling O; Shore-Lesserson L | SPECIFIC POPULATION ( Generla population, obese woman, general anaesthesia) |
| Corticosteroids for preventing neonatal respiratory morbidity after elective caesarean section at term. | Sotiriadis A; Makrydimas G; Papatheodorou S; Ioannidis JP | UPADTED/OLD VERSION |
| Corticosteroids for preventing neonatal respiratory morbidity after elective caesarean section at term. | Sotiriadis A; Makrydimas G; Papatheodorou S; Ioannidis JP; McGoldrick E | UPADTED/OLD VERSION |
| Antenatal steroid administration in medically uncomplicated pregnancy beyond 37 weeks of gestation for the prevention of neonatal morbidities prior to elective caesarean section: a systematic review and meta-analysis of randomised controlled trials. | Srinivasjois R; Silva D | UPADTED/OLD VERSION |
| Uterine caesarean closure techniques affect ultrasound findings and maternal outcomes: a systematic review and meta-analysis. | Stegwee SI; Jordans I; van der Voet LF; van de Ven PM; Ket J; Lambalk CB; de Groot C; Hehenkamp W; Huirne J | NOT OUTCOMES OF INTEREST |
| Systematic review of oxytocin dosing at caesarean section. | Stephens LC; Bruessel T | NOT A SR (Guidelines, Checklist, Review of review, Descriptive analysis, Review of literature) |
| Carbetocin for preventing postpartum haemorrhage. | Su LL; Chong YS; Samuel M | UPADTED/OLD VERSION |
| Oxytocin agonists for preventing postpartum haemorrhage. | Su LL; Chong YS; Samuel M | UPADTED/OLD VERSION |
| Enhanced recovery after caesarean delivery versus standard care studies: a systematic review of interventions and outcomes. | Sultan P; Sharawi N; Blake L; Carvalho B | NOT A SR (Guidelines, Checklist, Review of review, Descriptive analysis, Review of literature) |
| Impact of enhanced recovery after cesarean delivery on maternal outcomes: A systematic review and meta-analysis. | Sultan P; Sharawi N; Blake L; Habib AS; Brookfield KF; Carvalho B | NOT A SR (Guidelines, Checklist, Review of review, Descriptive analysis, Review of literature) |
| Prophylactic administration of cefazolin prior to skin incision versus antibiotics at cord clamping in preventing postcesarean infectious morbidity: a systematic review and meta-analysis of randomized controlled trials. | Sun J; Ding M; Liu J; Li Y; Sun X; Liu T; Chen Y | UPADTED/OLD VERSION |
| Effects of Abdominal Binders on Postoperative Pain and Functional Recovery: A Systematic Review and Meta-Analysis. | Sun X; Wei Q; Fu C; Zhang Q; Liang Z; Peng L; Chen L; He C | SPECIFIC POPULATION ( Generla population, obese woman, general anaesthesia) |
| Paucity of evidence for the effectiveness of prophylactic low-dose oxytocin protocols (<5â€ŠIU) compared with 5â€ŠIU in women undergoing elective caesarean section: A systematic review of randomised controlled trials. | Terblanche NCS; Picone DS; Otahal P; Sharman JE | UPADTED/OLD VERSION |
| Emerging concepts in antibiotic prophylaxis for cesarean delivery: a systematic review. | Tita ATN; Rouse DJ; Blackwell S; Saade GR; Spong CY; Andrews WW | NOT A SR (Guidelines, Checklist, Review of review, Descriptive analysis, Review of literature) |
| Chlorhexidine-Alcohol Compared with Povidone-Iodine Preoperative Skin Antisepsis for Cesarean Delivery: A Systematic Review and Meta-Analysis. | Tolcher MC; Whitham MD; El-Nashar SA; Clark SL | UPADTED/OLD VERSION |
| Prophylaxis for venous thromboembolic disease in pregnancy and the early postnatal period. | Tooher R; Gates S; Dowswell T; Davis LJ | UPADTED/OLD VERSION |
| A systematic review and meta-analysis of the effect of prophylactic tranexamic acid treatment in major benign uterine surgery. | Topsoee MF; Settnes A; Ottesen B; Bergholt T | UPADTED/OLD VERSION |
| Effects of Ondansetron on Attenuating Spinal Anesthesiaâ€“Induced Hypotension and Bradycardia in Obstetric and Nonobstetric Subjects: A Systematic Review and Meta-Analysis. | Tubog TD; Kane TD; Pugh MA | SPECIFIC POPULATION ( Generla population, obese woman, general anaesthesia) |
| Minimum Effective Dose (ED(50) and ED(95)) of Intrathecal Hyperbaric Bupivacaine for Cesarean Delivery: A Systematic Review. | Tubog TD; Ramsey VL; Filler L; Bramble RS | NOT OUTCOMES OF INTEREST |
| Staples compared with subcuticular suture for skin closure after cesarean delivery: a systematic review and meta-analysis. | Tuuli MG; Rampersad RM; Carbone JF; Stamilio D; Macones GA; Odibo AO | UPADTED/OLD VERSION |
| The Health Impact of Surgical Techniques and Assistive Methods Used in Cesarean Deliveries: A Systemic Review. | Wang LH; Seow KM; Chen LR; Chen KH | NOT A SR (Guidelines, Checklist, Review of review, Descriptive analysis, Review of literature) |
| Local anaesthetics and regional anaesthesia versus conventional analgesia for preventing persistent postoperative pain in adults and children. | Weinstein EJ; Levene JL; Cohen MS; Andreae DA; Chao JY; Johnson M; Hall CB; Andreae MH | UPADTED/OLD VERSION |
| Prophylactic Negative Pressure Wound Therapy in Closed Abdominal Incisions: A Meta-analysis of Randomised Controlled Trials. | Wells CI; Ratnayake CBB; Perrin J; Pandanaboyana S | SPECIFIC POPULATION ( Generla population, obese woman, general anaesthesia) |
| Chewing gum for intestinal function recovery after caesarean section: a systematic review and meta-analysis. | Wen Z; Shen M; Wu C; Ding J; Mei B | UPADTED/OLD VERSION |
| Comparison of Supraglottic Airway Devices With Endotracheal Intubation in Low-Risk Patients for Cesarean Delivery: Systematic Review and Meta-analysis. | White LD; Thang C; Hodsdon A; Melhuish TM; Barron FA; Godsall MG; Vlok R | NOT OUTCOMES OF INTEREST |
| Absorbable staples for uterine incision at caesarean section. | Wilkinson C; Enkin MW | UPADTED/OLD VERSION |
| Lateral tilt for caesarean section. | Wilkinson C; Enkin MW | UPADTED/OLD VERSION |
| Manual removal of placenta at caesarean section. | Wilkinson C; Enkin MW | UPADTED/OLD VERSION |
| Uterine exteriorization versus intraperitoneal repair at caesarean section. | Wilkinson C; Enkin MW | UPADTED/OLD VERSION |
| Peritoneal non-closure at caesarean section. | Wilkinson CS; Enkin MW | UPADTED/OLD VERSION |
| Guidelines for Antenatal and Preoperative care inÂ Cesarean Delivery: Enhanced Recovery After SurgeryÂ Society Recommendations (PartÂ 1). | Wilson RD; Caughey AB; Wood SL; Macones GA; Wrench IJ; Huang J; Norman M; Pettersson K; Fawcett WJ; Shalabi MM; Metcalfe A; Gramlich L; Nelson G | NOT A SR (Guidelines, Checklist, Review of review, Descriptive analysis, Review of literature) |
| Phenylephrine vs ephedrine in cesarean delivery under spinal anesthesia: A systematic literature review and meta-analysis. | Xu C; Liu S; Huang Y; Guo X; Xiao H; Qi D | UPADTED/OLD VERSION |
| Blunt vs. sharp uterine expansion at lower segment cesarean section delivery: a systematic review with metaanalysis. | Xu LL; Chau AM; Zuschmann A | UPADTED/OLD VERSION |
| The efficacy and safety of pharmacologic thromboprophylaxis following caesarean section: A systematic review and meta-analysis. | Yang R; Zhao X; Yang Y; Huang X; Li H; Su L | UPADTED/OLD VERSION |
| Prophylactic negative-pressure wound therapy after cesarean is associated with reduced risk of surgical site infection: aÂ systematic review and meta-analysis. | Yu L; Kronen RJ; Simon LE; Stoll CRT; Colditz GA; Tuuli MG | SPECIFIC POPULATION ( Generla population, obese woman, general anaesthesia) |
| Uterine exteriorization compared with in situ repair for Cesarean delivery: a systematic review and meta-analysis. | Zaphiratos V; George RB; Boyd JC; Habib AS | UPADTED/OLD VERSION |
| Timing of Antibiotic Prophylaxis in Elective Caesarean Delivery: A Multi-Center Randomized Controlled Trial and Meta-Analysis. | Zhang C; Zhang L; Liu X; Zeng Z; Li L; Liu G; Jiang H | NOT A SR (Guidelines, Checklist, Review of review, Descriptive analysis, Review of literature) |
| Dexmedetomidine as a neuraxial adjuvant for prevention of perioperative shivering: Meta-analysis of randomized controlled trials. | Zhang J; Zhang X; Wang H; Zhou H; Tian T; Wu A | NOT OUTCOMES OF INTEREST |
| Foetal responses to dexmedetomidine in parturients undergoing caesarean section: a systematic review and meta-analysis. | Zhang J; Zhou H; Sheng K; Tian T; Wu A | NOT OUTCOMES OF INTEREST |
| Effects of gum chewing on postoperative bowel motility after caesarean section: a meta-analysis of randomised controlled trials. | Zhu YP; Wang WJ; Zhang SL; Dai B; Ye DW | UPADTED/OLD VERSION |
| Comparison of the Joel-Cohen-based technique and the transverse Pfannenstiel for caesarean section for safety and effectiveness: A systematic review and meta-analysis. | Olyaeemanesh A; Bavandpour E; Mobinizadeh M; Ashrafinia M; Bavandpour M; Nouhi M | UNCERTAIN DATA INTEGRITY |
| Transversus abdominis plane block for postoperative analgesia after Caesarean delivery performed under spinal anaesthesia? A systematic review and meta-analysis. | Abdallah FW; Halpern SH; Margarido CB | STUDIES ALREADY INCLUDED IN CSR ORNCSR LATEST VERSION |
| Regional versus general anaesthesia for caesarean section. | Afolabi BB; Lesi FE; Merah NA | UPADTED/OLD VERSION |
| Techniques and materials for skin closure in caesarean section. | Alderdice F; McKenna D; Dornan J | UPADTED/OLD VERSION |
| Different classes of antibiotics given to women routinely for preventing infection at caesarean section. | Alfirevic Z; Gyte GM; Dou L | UPADTED/OLD VERSION |
| P6 stimulation for the prevention of nausea and vomiting associated with cesarean delivery under neuraxial anesthesia: a systematic review of randomized controlled trials. | Allen TK; Habib AS | NOT A SR (Guidelines, Checklist, Review of review, Descriptive analysis, Review of literature) |
| Efficacy of perioperative cefuroxime as a prophylactic antibiotic in women requiring caesarean section: A systematic review. | Alrammaal HH; Batchelor HK; Morris RK; Chong HP | NOT A SR (Guidelines, Checklist, Review of review, Descriptive analysis, Review of literature) |
| Local anaesthetics and regional anaesthesia for preventing chronic pain after surgery. | Andreae MH; Andreae DA | UPADTED/OLD VERSION |
| Timing of administration of prophylactic antibiotics for caesarean section: a systematic review and meta-analysis. | Baaqeel H; Baaqeel R | UPADTED/OLD VERSION |
| Prophylactic Dose of Oxytocin for Uterine Atony during Caesarean Delivery: A Systematic Review. | Baliuliene V; Vitartaite M; Rimaitis K | NOT A SR (Guidelines, Checklist, Review of review, Descriptive analysis, Review of literature) |
| Effect of Perioperative Active Body Surface Warming Systems on Analgesic and Clinical Outcomes: A Systematic Review and Meta-analysis of Randomized Controlled Trials. | Balki I; Khan JS; Staibano P; Duceppe E; Bessissow A; Sloan EN; Morley EE; Thompson AN; Devereaux B; Rojas C; Siddiqui N; Sessler DI; Devereaux PJ | SPECIFIC POPULATION ( Generla population, obese woman, general anaesthesia) |
| Caesarean section wound infiltration with local anaesthesia for postoperative pain relief - any benefit? | Bamigboye AA; Hofmeyr GJ | STUDIES ALREADY INCLUDED IN CSR ORNCSR LATEST VERSION |
| Closure versus non-closure of the peritoneum at caesarean section. | Bamigboye AA; Hofmeyr GJ | UPADTED/OLD VERSION |
| Non-closure of peritoneal surfaces at caesarean section--a systematic review. | Bamigboye AA; Hofmeyr GJ | UPADTED/OLD VERSION |
| Uterine externalization versus in situ repair of hysterotomy during cesarean delivery: a systematic review, equivalence meta-analysis, and trial sequential analysis. | Bhat A; Jaffer D; Keasler P; Kamath K; Kelly J; Singh PM | UPADTED/OLD VERSION |
| [Antimicrobial prophylaxis for caesarean delivery: before or after cord clamping? A meta-analysis]. | Boselli E; Bouvet L; RimmelÃ© T; Chassard D; Allaouchiche B | UPADTED/OLD VERSION |
| Intraoperative prophylactic and therapeutic non-invasive ventilation: a systematic review. | Cabrini L; Nobile L; Plumari VP; Landoni G; Borghi G; Mucchetti M; Zangrillo A | NOT OUTCOMES OF INTEREST |
| Vaginal Cleansing Before Cesarean Delivery: A Systematic Review and Meta-analysis. | Caissutti C; Saccone G; Zullo F; Quist-Nelson J; Felder L; Ciardulli A; Berghella V | STUDIES ALREADY INCLUDED IN CSR ORNCSR LATEST VERSION |
| Evidence-Based Bundles and Cesarean Delivery Surgical Site Infections: A Systematic Review and Meta-analysis. | Carter EB; Temming LA; Fowler S; Eppes C; Gross G; Srinivas SK; Macones GA; Colditz GA; Tuuli MG | NOT A SR (Guidelines, Checklist, Review of review, Descriptive analysis, Review of literature) |
| Guidelines for intraoperative care in cesarean delivery: Enhanced Recovery After Surgery Society Recommendations (Part 2). | Caughey AB; Wood SL; Macones GA; Wrench IJ; Huang J; Norman M; Pettersson K; Fawcett WJ; Shalabi MM; Metcalfe A; Gramlich L; Nelson G; Wilson RD | NOT A SR (Guidelines, Checklist, Review of review, Descriptive analysis, Review of literature) |
| Supplemental oxygen for caesarean section during regional anaesthesia. | Chatmongkolchart S; Prathep S | UPADTED/OLD VERSION |
| Suture closure of subcutaneous fat and wound disruption after cesarean delivery: a meta-analysis. | Chelmow D; Rodriguez EJ; Sabatini MM | UPADTED/OLD VERSION |
| To close or not to close? A systematic review and a meta-analysis of peritoneal non-closure and adhesion formation after caesarean section. | Cheong YC; Premkumar G; Metwally M; Peacock JL; Li TC | UPADTED/OLD VERSION |
| Techniques for preventing hypotension during spinal anaesthesia for caesarean section. | Chooi C; Cox JJ; Lumb RS; Middleton P; Chemali M; Emmett RS; Simmons SW; Cyna AM | UPADTED/OLD VERSION |
| Chewing gum improves postoperative recovery of gastrointestinal function after cesarean delivery: a systematic review and meta-analysis of randomized trials. | Ciardulli A; Saccone G; Di Mascio D; Caissutti C; Berghella V | STUDIES ALREADY INCLUDED IN CSR ORNCSR LATEST VERSION |
| Maternal position during caesarean section for preventing maternal and neonatal complications. | Cluver C; Novikova N; Hofmeyr GJ; Hall DR | UPADTED/OLD VERSION |
| Outcomes of second stage cesarean section following the use of a fetal head elevation device: A systematic review and meta-analysis. | Conde-Agudelo A; Nieto A; Rosas-Bermudez A; Romero R | UPADTED/OLD VERSION |
| A systematic review of the effects of adding neostigmine to local anesthetics for neuraxial administration in obstetric anesthesia and analgesia. | Cossu AP; De Giudici LM; Piras D; Mura P; Scanu M; Cossu M; Saba M; Finco G; Brazzi L | NOT OUTCOMES OF INTEREST |
| Chewing gum in preventing postoperative ileus in women undergoing caesarean section: a systematic review and meta-analysis of randomised controlled trials. | Craciunas L; Sajid MS; Ahmed AS | UPADTED/OLD VERSION |
| Intrathecal clonidine as an adjuvant for neuraxial anaesthesia during caesarean delivery: a systematic review and meta-analysis of randomised trials. | Crespo S; Dangelser G; Haller G | NOT OUTCOMES OF INTEREST |
| Techniques for preventing hypotension during spinal anaesthesia for caesarean section. | Cyna AM; Andrew M; Emmett RS; Middleton P; Simmons SW | UPADTED/OLD VERSION |
| Intraoperative and postoperative analgesic efficacy and adverse effects of intrathecal opioids in patients undergoing cesarean section with spinal anesthesia: a qualitative and quantitative systematic review of randomized controlled trials. | Dahl JB; Jeppesen IS; JÃ¸rgensen H; Wetterslev J; MÃ¸iniche S | UPADTED/OLD VERSION |
| Evidence-based surgery for cesarean delivery: an updated systematic review. | Dahlke JD; Mendez-Figueroa H; Rouse DJ; Berghella V; Baxter JK; Chauhan SP | NOT A SR (Guidelines, Checklist, Review of review, Descriptive analysis, Review of literature) |
| Preventing Hypothermia during Cesarean Birth: An Integrative Review. | Dendis M; Hooven K | NOT A SR (Guidelines, Checklist, Review of review, Descriptive analysis, Review of literature) |
| Outcomes of second stage cesarean section following the use of a fetal head elevation device: A systematic review and meta-analysis. | Di Girolamo R; Galliani C; Buca D; Liberati M; D'Antonio F | NOT A SR (Guidelines, Checklist, Review of review, Descriptive analysis, Review of literature) |
| The efficacy of abdominal binders in reducing postoperative pain and distress after cesarean delivery: A meta-analysis of randomized controlled trials. | Di Mascio D; Caruso G; Prata G; Saccone G; Terrin G; Giancotti A; Brunelli R; Muzii L; Benedetti Panici P; Di Donato V | STUDIES ALREADY INCLUDED IN CSR ORNCSR LATEST VERSION |
| Risk of Cesarean scar defect following single- vs double-layer uterine closure: systematic review and meta-analysis of randomized controlled trials. | Di Spiezio Sardo A; Saccone G; McCurdy R; Bujold E; Bifulco G; Berghella V | STUDIES ALREADY INCLUDED IN CSR ORNCSR LATEST VERSION |
| Surgical techniques for uterine incision and uterine closure at the time of caesarean section. | Dodd JM; Anderson ER; Gates S | UPADTED/OLD VERSION |
| Tocolysis for assisting delivery at caesarean section. | Dodd JM; Reid K | UPADTED/OLD VERSION |
| Techniques for preventing hypotension during spinal anaesthesia for caesarean section. | Emmett RS; Cyna AM; Andrew M; Simmons SW | UPADTED/OLD VERSION |
| Techniques for preventing hypotension during spinal anaesthesia for caesarean section. | Emmett RS; Cyna AM; Andrew M; Simmons SW | UPADTED/OLD VERSION |
| Single versus two layer suturing for closing the uterine incision at caesarean section. | Enkin MW; Wilkinson C | UPADTED/OLD VERSION |
| [Efficacy and safety of tranexamic acid administration for the prevention and/or the treatment of post-partum haemorrhage: a systematic review with meta-analysis]. | Faraoni D; Carlier C; Samama CM; Levy JH; Ducloy-Bouthors AS | UPADTED/OLD VERSION |
| Are prophylactic adjunctive macrolides efficacious against caesarean section surgical site infection: A systematic review and meta-analysis. | Farmer N; Hodgetts-Morton V; Morris RK | UPADTED/OLD VERSION |
| Epidemiology, prevention and management of early postpartum hemorrhage - a systematic review. | Feduniw S; Warzecha D; Szymusik I; Wielgos M | NOT A SR (Guidelines, Checklist, Review of review, Descriptive analysis, Review of literature) |
| Prevention of hypotension after spinal anaesthesia for caesarean section: a systematic review and network meta-analysis of randomised controlled trials. | Fitzgerald JP; Fedoruk KA; Jadin SM; Carvalho B; Halpern SH | NOT A SR (Guidelines, Checklist, Review of review, Descriptive analysis, Review of literature) |
| Safety and efficacy of tranexamic acid for prevention of obstetric haemorrhage: an updated systematic review and meta-analysis. | Franchini M; Mengoli C; Cruciani M; Bergamini V; Presti F; Marano G; Pupella S; Vaglio S; Masiello F; Veropalumbo E; Piccinini V; Pati I; Liumbruno GM | STUDIES ALREADY INCLUDED IN CSR ORNCSR LATEST VERSION |
| [Continuous infusion of local anesthetic at the site of the abdominal surgical wound for postoperative analgesia: a systematic review]. | Fustran Guerrero N; Dalmau LlitjÃ³s A; SabatÃ© Pes A | NOT A SR (Guidelines, Checklist, Review of review, Descriptive analysis, Review of literature) |
| Wound drainage for caesarean section. | Gates S; Anderson ER | UPADTED/OLD VERSION |
| Prophylaxis for venous thromboembolic disease in pregnancy and the early postnatal period. | Gates S; Brocklehurst P; Davis LJ | UPADTED/OLD VERSION |
| Effect of negative-pressure wound therapy on wound complications in obese women after caesarean birth: a systematic review and meta-analysis. | Gillespie BM; Thalib L; Ellwood D; Kang E; Mahomed K; Kumar S; Chaboyer W | SPECIFIC POPULATION ( Generla population, obese woman, general anaesthesia) |
| Effects of colloid preload on the incidence of hypotension in spinal anesthesia for cesarean section: a systematic review and meta-analysis. | Gong RS; Liu XW; Li WX; Zhao J | STUDIES ALREADY INCLUDED IN CSR ORNCSR LATEST VERSION |
| Interventions for preventing nausea and vomiting in women undergoing regional anaesthesia for caesarean section. | Griffiths JD; Gyte GM; Paranjothy S; Brown HC; Broughton HK; Thomas J | UPADTED/OLD VERSION |
| The safety of early postpartum discharge: a review and critique. | Grullon KE; Grimes DA | UPADTED/OLD VERSION |
| Prophylactic negative pressure wound therapy on surgical site infection in obese women after cesarean section: A systematic review and meta-analysis. | Guo C; Cheng T; Li J | SPECIFIC POPULATION ( Generla population, obese woman, general anaesthesia) |
| Different classes of antibiotics given to women routinely for preventing infection at caesarean section. | Gyte GM; Dou L; Vazquez JC | UPADTED/OLD VERSION |
| Vaginal preparation with antiseptic solution before cesarean section for preventing postoperative infections. | Haas DM; Morgan Al Darei S; Contreras K | UPADTED/OLD VERSION |
| Vaginal preparation with antiseptic solution before cesarean section for preventing postoperative infections. | Haas DM; Morgan S; Contreras K | UPADTED/OLD VERSION |
| Vaginal preparation with antiseptic solution before cesarean section for preventing postoperative infections. | Haas DM; Morgan S; Contreras K | UPADTED/OLD VERSION |
| Vaginal preparation with antiseptic solution before cesarean section for preventing postoperative infections. | Haas DM; Morgan S; Contreras K; Enders S | UPADTED/OLD VERSION |
| Skin preparation for preventing infection following caesarean section. | Hadiati DR; Hakimi M; Nurdiati DS | UPADTED/OLD VERSION |
| Skin preparation for preventing infection following caesarean section. | Hadiati DR; Hakimi M; Nurdiati DS; da Silva Lopes K; Ota E | UPADTED/OLD VERSION |
| Skin preparation for preventing infection following caesarean section. | Hadiati DR; Hakimi M; Nurdiati DS; Ota E | UPADTED/OLD VERSION |
| Staples versus subcuticular suture for cesarean skin closure in obese women: A systematic review and meta-analysis. | Han D; Feng L; Xu L; Li C; Zhang Q | SPECIFIC POPULATION ( Generla population, obese woman, general anaesthesia) |
| Intravenous ketamine during spinal and general anaesthesia for caesarean section: systematic review and meta-analysis. | Heesen M; BÃ¶hmer J; Brinck EC; Kontinen VK; KlÃ¶hr S; Rossaint R; Straube S | STUDIES ALREADY INCLUDED IN CSR ORNCSR LATEST VERSION |
| Is general anaesthesia for caesarean section associated with postpartum haemorrhage? Systematic review and meta-analysis. | Heesen M; Hofmann T; KlÃ¶hr S; Rossaint R; van de Velde M; Deprest J; Straube S | STUDIES ALREADY INCLUDED IN CSR ORNCSR LATEST VERSION |
| Prophylactic phenylephrine for caesarean section under spinal anaesthesia: systematic review and meta-analysis. | Heesen M; KÃ¶lhr S; Rossaint R; Straube S | NOT OUTCOMES OF INTEREST |
| Maternal and foetal effects of remifentanil for general anaesthesia in parturients undergoing caesarean section: a systematic review and meta-analysis. | Heesen M; KlÃ¶hr S; Hofmann T; Rossaint R; Devroe S; Straube S; Van de Velde M | SPECIFIC POPULATION ( Generla population, obese woman, general anaesthesia) |
| Concerning the timing of antibiotic administration in women undergoing caesarean section: a systematic review and meta-analysis. | Heesen M; KlÃ¶hr S; Rossaint R; Allegaert K; Allegeaert K; Deprest J; Van de Velde M; Straube S | UPADTED/OLD VERSION |
| Prevention of Spinal Anesthesia-Induced Hypotension During Cesarean Delivery by 5-Hydroxytryptamine-3 Receptor Antagonists: A Systematic Review and Meta-analysis and Meta-regression. | Heesen M; Klimek M; Hoeks SE; Rossaint R | STUDIES ALREADY INCLUDED IN CSR ORNCSR LATEST VERSION |
| Prophylactic subcutaneous drainage for prevention of wound complications after cesarean delivery--a metaanalysis. | Hellums EK; Lin MG; Ramsey PS | UPADTED/OLD VERSION |
| Hyperbaric versus plain bupivacaine for spinal anesthesia for cesarean delivery. | Heng Sia AT; Tan KH; Sng BL; Lim Y; Chan ESY; Siddiqui FJ | UPADTED/OLD VERSION |
| Gum chewing and gastrointestinal function following caesarean delivery: a systematic review and meta-analysis. | Hochner H; Tenfelde SM; Abu Ahmad W; Liebergall-Wischnitzer M | UPADTED/OLD VERSION |
| Antibiotic prophylaxis regimens and drugs for cesarean section. | Hopkins L; Smaill F | UPADTED/OLD VERSION |
| Information for pregnant women about caesarean birth. | Horey D; Weaver J; Russell H | NOT OUTCOMES OF INTEREST |
| Hypnotic agents for induction of general anesthesia in cesarean section patients: A systematic review and meta-analysis of randomized controlled trials. | Houthoff Khemlani K; Weibel S; Kranke P; Schreiber JU | SPECIFIC POPULATION ( Generla population, obese woman, general anaesthesia) |
| Effect of misoprostol versus oxytocin during caesarean section: a systematic review and meta-analysis. | Hua J; Chen G; Xing F; Scott M; Li Q | UPADTED/OLD VERSION |
| Usefulness of chewing gum for recovering intestinal function after cesarean delivery: A systematic review and meta-analysis of randomized controlled trials. | Huang HP; He M | UPADTED/OLD VERSION |
| Implementation of vaginal preparation prior to caesarean section. | Jakes AD; Bell A; Chiwera L; Lloyd J | NOT A SR (Guidelines, Checklist, Review of review, Descriptive analysis, Review of literature) |
| Carbetocin for the prevention of postpartum hemorrhage: a systematic review and meta-analysis of randomized controlled trials. | Jin B; Du Y; Zhang F; Zhang K; Wang L; Cui L | UPADTED/OLD VERSION |
| Single injection Quadratus Lumborum block for postoperative analgesia in adult surgical population: A systematic review and meta-analysis. | Jin Z; Liu J; Li R; Gan TJ; He Y; Lin J | STUDIES ALREADY INCLUDED IN CSR ORNCSR LATEST VERSION |
| [Early post-partum discharge : systematic review of the literature]. | Jonguitud-Aguilar A; Tomasso G; Cafferatta ML | NOT A SR (Guidelines, Checklist, Review of review, Descriptive analysis, Review of literature) |
| A scoping review of maternal antibiotic prophylaxis in low- and middle-income countries: Comparison to WHO recommendations for prevention and treatment of maternal peripartum infection. | Jury I; Thompson K; Hirst JE | NOT A SR (Guidelines, Checklist, Review of review, Descriptive analysis, Review of literature) |
| Efficacy of carbetocin in the prevention of postpartum hemorrhage: a systematic review and Bayesian meta-analysis of randomized trials. | Kalafat E; Gokce A; O'Brien P; Benlioglu C; Koc A; Karaaslan O; Khalil A | UPADTED/OLD VERSION |
| Does tranexamic acid prevent postpartum haemorrhage? A systematic review of randomised controlled trials. | Ker K; Shakur H; Roberts I | UPADTED/OLD VERSION |
| Combined spinal-epidural vs. spinal anaesthesia for caesarean section: meta-analysis and trial-sequential analysis. | Klimek M; Rossaint R; van de Velde M; Heesen M | UPADTED/OLD VERSION |
| A meta-analysis of the effect of inspired oxygen concentration on the incidence of surgical site infection following cesarean section. | Klingel ML; Patel SV | NOT OUTCOMES OF INTEREST |
| Evidence-based value of subcutaneous surgical wound drainage: the largest systematic review and meta-analysis. | Kosins AM; Scholz T; Cetinkaya M; Evans GRD | STUDIES ALREADY INCLUDED IN CSR ORNCSR LATEST VERSION |
| A systematic review of the cost-effectiveness of uterotonic agents for the prevention of postpartum hemorrhage. | Lawrie TA; RogoziÅ„ska E; Sobiesuo P; Vogel JP; Ternent L; Oladapo OT | NOT OUTCOMES OF INTEREST |
| A dose-response meta-analysis of prophylactic intravenous ephedrine for the prevention of hypotension during spinal anesthesia for elective cesarean delivery. | Lee A; Ngan Kee WD; Gin T | STUDIES ALREADY INCLUDED IN CSR ORNCSR LATEST VERSION |
| A quantitative, systematic review of randomized controlled trials of ephedrine versus phenylephrine for the management of hypotension during spinal anesthesia for cesarean delivery. | Lee A; Ngan Kee WD; Gin T | STUDIES ALREADY INCLUDED IN CSR ORNCSR LATEST VERSION |
| Prophylactic ephedrine prevents hypotension during spinal anesthesia for Cesarean delivery but does not improve neonatal outcome: a quantitative systematic review. | Lee A; Ngan Kee WD; Gin T | STUDIES ALREADY INCLUDED IN CSR ORNCSR LATEST VERSION |
| Effect of intravenous dexmedetomidine and remifentanil on neonatal outcomes after caesarean section under general anaesthesia: A systematic review and meta-analysis. | Lee M; Kim H; Lee C; Kang H | SPECIFIC POPULATION ( Generla population, obese woman, general anaesthesia) |
| Is prophylactic tranexamic acid administration effective and safe for postpartum hemorrhage prevention?: A systematic review and meta-analysis. | Li C; Gong Y; Dong L; Xie B; Dai Z | STUDIES ALREADY INCLUDED IN CSR ORNCSR LATEST VERSION |
| Is routine indwelling catheterisation of the bladder for caesarean section necessary? A systematic review. | Li L; Wen J; Wang L; Li YP; Li Y | STUDIES ALREADY INCLUDED IN CSR ORNCSR LATEST VERSION |
| Comparing prophylactic use of cefazolin for SSI in cesarean section: a systematic review and meta-analysis. | Li M; Shi B; Ma J; Peng X; Shi J | STUDIES ALREADY INCLUDED IN CSR ORNCSR LATEST VERSION |
| Subarachnoid and epidural dexmedetomidine for the prevention of post-anesthetic shivering: a meta-analysis and systematic review. | Li YZ; Jiang Y; Lin H; Yang XP | SPECIFIC POPULATION ( Generla population, obese woman, general anaesthesia) |
| Mechanical dilatation of the cervix at non-labour caesarean section for reducing postoperative morbidity. | Liabsuetrakul T; Peeyananjarassri K | UPADTED/OLD VERSION |
| Effect of Intravenous Ketamine on Hypocranial Pressure Symptoms in Patients with Spinal Anesthetic Cesarean Sections: A Systematic Review and Meta-Analysis. | Liang X; Yang X; Liang S; Zhang Y; Ding Z; Guo Q; Huang C | NOT OUTCOMES OF INTEREST |
| Shivering prevention and treatment during cesarean delivery under neuraxial anesthesia: a systematic review. | Liu J; Wang Y; Ma W | NOT A SR (Guidelines, Checklist, Review of review, Descriptive analysis, Review of literature) |
| Intraoperative interventions for preventing surgical site infection: an overview of Cochrane Reviews. | Liu Z; Dumville JC; Norman G; Westby MJ; Blazeby J; McFarlane E; Welton NJ; O'Connor L; Cawthorne J; George RP; Crosbie EJ; Rithalia AD; Cheng HY | NOT A SR (Guidelines, Checklist, Review of review, Descriptive analysis, Review of literature) |
| Guidelines for postoperative care in cesarean delivery: Enhanced Recovery After Surgery (ERAS) Society recommendations (part 3). | Macones GA; Caughey AB; Wood SL; Wrench IJ; Huang J; Norman M; Pettersson K; Fawcett WJ; Shalabi MM; Metcalfe A; Gramlich L; Nelson G; Wilson RD | NOT A SR (Guidelines, Checklist, Review of review, Descriptive analysis, Review of literature) |
| Preincision adjunctive prophylaxis for cesarean deliveries a systematic review and meta-analysis. | Markwei MT; Babatunde I; Rathi N; Fan C; Prah MA; Joo J; Hackett L; Soper DE; Goje O | STUDIES ALREADY INCLUDED IN CSR ORNCSR LATEST VERSION |
| Best practice perioperative strategies and surgical techniques for preventing caesarean section surgical site infections: a systematic review of reviews and meta-analyses. | Martin EK; Beckmann MM; Barnsbee LN; Halton KA; Merollini K; Graves N | NOT A SR (Guidelines, Checklist, Review of review, Descriptive analysis, Review of literature) |
| Abdominal surgical incisions for caesarean section. | Mathai M; Hofmeyr GJ | UPADTED/OLD VERSION |
| The association of skin incision placement during cesarean delivery with wound complications in obese women: a systematic review and meta-analysis. | Mccurdy RJ; Felder LA; Saccone G; Edwards RK; Thornburg LL; Marrs C; Conner SN; Strauss R; Berghella V | SPECIFIC POPULATION ( Generla population, obese woman, general anaesthesia) |
| Intravenous Magnesium Sulphate for Analgesia after Caesarean Section: A Systematic Review. | McKeown A; Seppi V; Hodgson R | NOT A SR (Guidelines, Checklist, Review of review, Descriptive analysis, Review of literature) |
| Practices to Reduce Surgical Site Infections Among Women Undergoing Cesarean Section: A Review. | McKibben RA; Pitts SI; Suarez-Cuervo C; Perl TM; Bass EB | NOT A SR (Guidelines, Checklist, Review of review, Descriptive analysis, Review of literature) |
| The Clinical Efficacy and Safety of Enhanced Recovery After Surgery for Cesarean Section: A Systematic Review and Meta-Analysis of Randomized Controlled Trials and Observational Studies. | Meng X; Chen K; Yang C; Li H; Wang X | NOT A SR (Guidelines, Checklist, Review of review, Descriptive analysis, Review of literature) |
| Transversus abdominis plane block for analgesia after Cesarean delivery: a systematic review and meta-analysis. | Mishriky BM; George RB; Habib AS | STUDIES ALREADY INCLUDED IN CSR ORNCSR LATEST VERSION |
| Metoclopramide for nausea and vomiting prophylaxis during and after Caesarean delivery: a systematic review and meta-analysis. | Mishriky BM; Habib AS | UPADTED/OLD VERSION |
| Effectiveness of strategies for the management and/or prevention of hypothermia within the adult perioperative environment. | Moola S; Lockwood C | NOT OUTCOMES OF INTEREST |
| The effects of an increase of central blood volume before spinal anesthesia for cesarean delivery: a qualitative systematic review. | Morgan PJ; Halpern SH; Tarshis J | NOT A SR (Guidelines, Checklist, Review of review, Descriptive analysis, Review of literature) |
| Codeine-acetaminophen versus nonsteroidal anti-inflammatory drugs in the treatment of post-abdominal surgery pain: a systematic review of randomized trials. | Nauta M; Landsmeer ML; Koren G | SPECIFIC POPULATION ( Generla population, obese woman, general anaesthesia) |
| Negative pressure wound therapy for surgical wounds healing by primary closure. | Norman G; Goh EL; Dumville JC; Shi C; Liu Z; Chiverton L; Stankiewicz M; Reid A | UPADTED/OLD VERSION |
| Negative pressure wound therapy for surgical wounds healing by primary closure. | Norman G; Goh EL; Dumville JC; Shi C; Liu Z; Chiverton L; Stankiewicz M; Reid A | UPADTED/OLD VERSION |
| Tranexamic acid for preventing postpartum haemorrhage. | Novikova N; Hofmeyr GJ | UPADTED/OLD VERSION |
| Effects of perioperative oxygen concentration on oxidative stress in adult surgical patients: a systematic review. | Oldman AH; Martin DS; Feelisch M; Grocott MPW; Cumpstey AF | SPECIFIC POPULATION ( Generla population, obese woman, general anaesthesia) |
| Carbetocin compared with oxytocin in non-elective Cesarean delivery: a systematic review, meta-analysis, and trial sequential analysis of randomized-controlled trials. | Onwochei DN; Owolabi A; Singh PM; Monks DT | UPADTED/OLD VERSION |
| Carbetocin reduces the need for additional uterotonics in elective caesarean delivery: a systematic review, meta-analysis and trial sequential analysis of randomised controlled trials. | Onwochei DN; Van Ross J; Singh PM; Salter A; Monks DT | UPADTED/OLD VERSION |
| Retrospective analysis on the efficacy of corticosteroid prophylaxis prior to elective caesarean section to reduce neonatal respiratory complications at term of pregnancy: review of literature. | Paganelli S; Soncini E; Gargano G; Capodanno F; Vezzani C; La Sala GB | NOT A SR (Guidelines, Checklist, Review of review, Descriptive analysis, Review of literature) |
| Interventions at caesarean section for reducing the risk of aspiration pneumonitis. | Paranjothy S; Griffiths JD; Broughton HK; Gyte GM; Brown HC; Thomas J | UPADTED/OLD VERSION |
| Interventions at caesarean section for reducing the risk of aspiration pneumonitis. | Paranjothy S; Griffiths JD; Broughton HK; Gyte GM; Brown HC; Thomas J | UPADTED/OLD VERSION |
| The Effect of Glycopyrrolate on the Incidence of Hypotension and Vasopressor Requirement During Spinal Anesthesia for Cesarean Delivery: A Meta-analysis. | Patel SD; Habib AS; Phillips S; Carvalho B; Sultan P | STUDIES ALREADY INCLUDED IN CSR ORNCSR LATEST VERSION |
| Prevention of Surgical Site Infections: A Systematic Review of Cost Analyses in the Use of Prophylactic Antibiotics. | Purba AKR; Setiawan D; Bathoorn E; Postma MJ; Dik JH; Friedrich AW | NOT A SR (Guidelines, Checklist, Review of review, Descriptive analysis, Review of literature) |
| Postoperative Discharge Opioid Consumption, Leftover, and Disposal after Obstetric and Gynecologic Procedures: A Systematic Review. | Raina J; Costello C; Suarthana E; Tulandi T | NOT OUTCOMES OF INTEREST |
| Autologous amniotic membrane: An accelerator of wound healing for prevention of surgical site infections following Cesarean delivery. | Rezazadeh D; Anvari Aliabad R; Norooznezhad AH | NOT A SR (Guidelines, Checklist, Review of review, Descriptive analysis, Review of literature) |
| Fluid loading therapy to prevent spinal hypotension in women undergoing elective caesarean section: Network meta-analysis, trial sequential analysis and meta-regression. | Rijs K; Mercier FJ; Lucas DN; Rossaint R; Klimek M; Heesen M | STUDIES ALREADY INCLUDED IN CSR ORNCSR LATEST VERSION |
| Analgesic efficacy of the ultrasound-guided blockade of the transversus abdominis plane - a systematic review. | RipollÃ©s J; Mezquita SM; Abad A; Calvo J | STUDIES ALREADY INCLUDED IN CSR ORNCSR LATEST VERSION |
| Colloids versus crystalloids in the prevention of hypotension induced by spinal anesthesia in elective cesarean section. A systematic review and meta-analysis. | RipollÃ©s Melchor J; Espinosa Ã; MartÃ­nez Hurtado E; Casans FrancÃ©s R; Navarro PÃ©rez R; Abad Gurumeta A; Calvo Vecino JM | STUDIES ALREADY INCLUDED IN CSR ORNCSR LATEST VERSION |
| Povidone-iodine 1% is the most effective vaginal antiseptic for preventing post-cesarean endometritis: aÂ systematic review and network meta-analysis. | Roeckner JT; Sanchez-Ramos L; Mitta M; Kovacs A; Kaunitz AM | UPADTED/OLD VERSION |
| PROSPECT guideline for elective caesarean section: updated systematic review and procedure-specific postoperative pain management recommendations. | Roofthooft E; Joshi GP; Rawal N; Van de Velde M | NOT A SR (Guidelines, Checklist, Review of review, Descriptive analysis, Review of literature) |
| A Systematic Review Evaluating Neuraxial Morphine and Diamorphine-Associated Respiratory Depression After Cesarean Delivery. | Sharawi N; Carvalho B; Habib AS; Blake L; Mhyre JM; Sultan P | NOT A SR (Guidelines, Checklist, Review of review, Descriptive analysis, Review of literature) |
| Evaluation of patient-reported outcome measures of functional recovery following caesarean section: a systematic review using the consensus-based standards for the selection of health measurement instruments (COSMIN) checklist. | Sharawi N; Klima L; Shah R; Blake L; Carvalho B; Sultan P | NOT A SR (Guidelines, Checklist, Review of review, Descriptive analysis, Review of literature) |
| Chewing gum for postoperative recovery of gastrointestinal function. | Short V; Herbert G; Perry R; Atkinson C; Ness AR; Penfold C; Thomas S; Andersen HK; Lewis SJ | UPADTED/OLD VERSION |
| Use of hyperbaric versus isobaric bupivacaine for spinal anaesthesia for caesarean section. | Sia AT; Tan KH; Sng BL; Lim Y; Chan ES; Siddiqui FJ | UPADTED/OLD VERSION |
| Tranexamic acid for preventing postpartum blood loss after cesarean delivery: a systematic review and meta-analysis of randomized controlled trials. | Simonazzi G; Bisulli M; Saccone G; Moro E; Marshall A; Berghella V | STUDIES ALREADY INCLUDED IN CSR ORNCSR LATEST VERSION |
| The analgesic effectiveness of ilioinguinal-iliohypogastric block for caesarean delivery: A meta-analysis and trial sequential analysis. | Singh NP; Makkar JK; Bhatia N; Singh PM | ARTICLE MISSING |
| The analgesic efficacy of quadratus lumborum block in caesarean delivery: a meta-analysis and trial sequential analysis. | Singh NP; Makkar JK; Borle A; Monks D; Goudra BG; Zorrilla-Vaca A; Singh PM | STUDIES ALREADY INCLUDED IN CSR ORNCSR LATEST VERSION |
| Antibiotic prophylaxis for cesarean section. | Smaill F; Hofmeyr GJ | UPADTED/OLD VERSION |
| Antibiotic prophylaxis for cesarean section. | Smaill F; Hofmeyr GJ | UPADTED/OLD VERSION |
| Antibiotic prophylaxis versus no prophylaxis for preventing infection after cesarean section. | Smaill FM; Gyte GM | UPADTED/OLD VERSION |
| Hyperbaric vs. isobaric bupivacaine for spinal anaesthesia for elective caesarean section: a Cochrane systematic review. | Sng BL; Han NLR; Leong WL; Sultana R; Siddiqui FJ; Assam PN; Chan ES; Tan KH; Sia AT | STUDIES ALREADY INCLUDED IN CSR ORNCSR LATEST VERSION |
| Single dose perioperative intrathecal ketamine as an adjuvant to intrathecal bupivacaine: A systematic review and meta-analysis of adult human randomized controlled trials. | Sohnen S; Dowling O; Shore-Lesserson L | SPECIFIC POPULATION ( Generla population, obese woman, general anaesthesia) |
| Corticosteroids for preventing neonatal respiratory morbidity after elective caesarean section at term. | Sotiriadis A; Makrydimas G; Papatheodorou S; Ioannidis JP | UPADTED/OLD VERSION |
| Corticosteroids for preventing neonatal respiratory morbidity after elective caesarean section at term. | Sotiriadis A; Makrydimas G; Papatheodorou S; Ioannidis JP; McGoldrick E | UPADTED/OLD VERSION |
| Antenatal steroid administration in medically uncomplicated pregnancy beyond 37 weeks of gestation for the prevention of neonatal morbidities prior to elective caesarean section: a systematic review and meta-analysis of randomised controlled trials. | Srinivasjois R; Silva D | UPADTED/OLD VERSION |
| Uterine caesarean closure techniques affect ultrasound findings and maternal outcomes: a systematic review and meta-analysis. | Stegwee SI; Jordans I; van der Voet LF; van de Ven PM; Ket J; Lambalk CB; de Groot C; Hehenkamp W; Huirne J | NOT OUTCOMES OF INTEREST |
| Systematic review of oxytocin dosing at caesarean section. | Stephens LC; Bruessel T | NOT A SR (Guidelines, Checklist, Review of review, Descriptive analysis, Review of literature) |
| Carbetocin for preventing postpartum haemorrhage. | Su LL; Chong YS; Samuel M | UPADTED/OLD VERSION |
| Oxytocin agonists for preventing postpartum haemorrhage. | Su LL; Chong YS; Samuel M | UPADTED/OLD VERSION |
| Enhanced recovery after caesarean delivery versus standard care studies: a systematic review of interventions and outcomes. | Sultan P; Sharawi N; Blake L; Carvalho B | NOT A SR (Guidelines, Checklist, Review of review, Descriptive analysis, Review of literature) |
| Impact of enhanced recovery after cesarean delivery on maternal outcomes: A systematic review and meta-analysis. | Sultan P; Sharawi N; Blake L; Habib AS; Brookfield KF; Carvalho B | NOT A SR (Guidelines, Checklist, Review of review, Descriptive analysis, Review of literature) |
| Prophylactic administration of cefazolin prior to skin incision versus antibiotics at cord clamping in preventing postcesarean infectious morbidity: a systematic review and meta-analysis of randomized controlled trials. | Sun J; Ding M; Liu J; Li Y; Sun X; Liu T; Chen Y | UPADTED/OLD VERSION |
| Effects of Abdominal Binders on Postoperative Pain and Functional Recovery: A Systematic Review and Meta-Analysis. | Sun X; Wei Q; Fu C; Zhang Q; Liang Z; Peng L; Chen L; He C | SPECIFIC POPULATION ( Generla population, obese woman, general anaesthesia) |
| Paucity of evidence for the effectiveness of prophylactic low-dose oxytocin protocols (<5â€ŠIU) compared with 5â€ŠIU in women undergoing elective caesarean section: A systematic review of randomised controlled trials. | Terblanche NCS; Picone DS; Otahal P; Sharman JE | UPADTED/OLD VERSION |
| Emerging concepts in antibiotic prophylaxis for cesarean delivery: a systematic review. | Tita ATN; Rouse DJ; Blackwell S; Saade GR; Spong CY; Andrews WW | NOT A SR (Guidelines, Checklist, Review of review, Descriptive analysis, Review of literature) |
| Chlorhexidine-Alcohol Compared with Povidone-Iodine Preoperative Skin Antisepsis for Cesarean Delivery: A Systematic Review and Meta-Analysis. | Tolcher MC; Whitham MD; El-Nashar SA; Clark SL | UPADTED/OLD VERSION |
| Prophylaxis for venous thromboembolic disease in pregnancy and the early postnatal period. | Tooher R; Gates S; Dowswell T; Davis LJ | UPADTED/OLD VERSION |
| A systematic review and meta-analysis of the effect of prophylactic tranexamic acid treatment in major benign uterine surgery. | Topsoee MF; Settnes A; Ottesen B; Bergholt T | UPADTED/OLD VERSION |
| Effects of Ondansetron on Attenuating Spinal Anesthesiaâ€“Induced Hypotension and Bradycardia in Obstetric and Nonobstetric Subjects: A Systematic Review and Meta-Analysis. | Tubog TD; Kane TD; Pugh MA | SPECIFIC POPULATION ( Generla population, obese woman, general anaesthesia) |
| Minimum Effective Dose (ED(50) and ED(95)) of Intrathecal Hyperbaric Bupivacaine for Cesarean Delivery: A Systematic Review. | Tubog TD; Ramsey VL; Filler L; Bramble RS | NOT OUTCOMES OF INTEREST |
| Staples compared with subcuticular suture for skin closure after cesarean delivery: a systematic review and meta-analysis. | Tuuli MG; Rampersad RM; Carbone JF; Stamilio D; Macones GA; Odibo AO | UPADTED/OLD VERSION |
| The Health Impact of Surgical Techniques and Assistive Methods Used in Cesarean Deliveries: A Systemic Review. | Wang LH; Seow KM; Chen LR; Chen KH | NOT A SR (Guidelines, Checklist, Review of review, Descriptive analysis, Review of literature) |
| Local anaesthetics and regional anaesthesia versus conventional analgesia for preventing persistent postoperative pain in adults and children. | Weinstein EJ; Levene JL; Cohen MS; Andreae DA; Chao JY; Johnson M; Hall CB; Andreae MH | UPADTED/OLD VERSION |
| Prophylactic Negative Pressure Wound Therapy in Closed Abdominal Incisions: A Meta-analysis of Randomised Controlled Trials. | Wells CI; Ratnayake CBB; Perrin J; Pandanaboyana S | SPECIFIC POPULATION ( Generla population, obese woman, general anaesthesia) |
| Chewing gum for intestinal function recovery after caesarean section: a systematic review and meta-analysis. | Wen Z; Shen M; Wu C; Ding J; Mei B | UPADTED/OLD VERSION |
| Comparison of Supraglottic Airway Devices With Endotracheal Intubation in Low-Risk Patients for Cesarean Delivery: Systematic Review and Meta-analysis. | White LD; Thang C; Hodsdon A; Melhuish TM; Barron FA; Godsall MG; Vlok R | NOT OUTCOMES OF INTEREST |
| Absorbable staples for uterine incision at caesarean section. | Wilkinson C; Enkin MW | UPADTED/OLD VERSION |
| Lateral tilt for caesarean section. | Wilkinson C; Enkin MW | UPADTED/OLD VERSION |
| Manual removal of placenta at caesarean section. | Wilkinson C; Enkin MW | UPADTED/OLD VERSION |
| Uterine exteriorization versus intraperitoneal repair at caesarean section. | Wilkinson C; Enkin MW | UPADTED/OLD VERSION |
| Peritoneal non-closure at caesarean section. | Wilkinson CS; Enkin MW | UPADTED/OLD VERSION |
| Guidelines for Antenatal and Preoperative care inÂ Cesarean Delivery: Enhanced Recovery After SurgeryÂ Society Recommendations (PartÂ 1). | Wilson RD; Caughey AB; Wood SL; Macones GA; Wrench IJ; Huang J; Norman M; Pettersson K; Fawcett WJ; Shalabi MM; Metcalfe A; Gramlich L; Nelson G | NOT A SR (Guidelines, Checklist, Review of review, Descriptive analysis, Review of literature) |
| Phenylephrine vs ephedrine in cesarean delivery under spinal anesthesia: A systematic literature review and meta-analysis. | Xu C; Liu S; Huang Y; Guo X; Xiao H; Qi D | UPADTED/OLD VERSION |
| Blunt vs. sharp uterine expansion at lower segment cesarean section delivery: a systematic review with metaanalysis. | Xu LL; Chau AM; Zuschmann A | UPADTED/OLD VERSION |
| The efficacy and safety of pharmacologic thromboprophylaxis following caesarean section: A systematic review and meta-analysis. | Yang R; Zhao X; Yang Y; Huang X; Li H; Su L | UPADTED/OLD VERSION |
| Prophylactic negative-pressure wound therapy after cesarean is associated with reduced risk of surgical site infection: aÂ systematic review and meta-analysis. | Yu L; Kronen RJ; Simon LE; Stoll CRT; Colditz GA; Tuuli MG | SPECIFIC POPULATION ( Generla population, obese woman, general anaesthesia) |
| Uterine exteriorization compared with in situ repair for Cesarean delivery: a systematic review and meta-analysis. | Zaphiratos V; George RB; Boyd JC; Habib AS | UPADTED/OLD VERSION |
| Timing of Antibiotic Prophylaxis in Elective Caesarean Delivery: A Multi-Center Randomized Controlled Trial and Meta-Analysis. | Zhang C; Zhang L; Liu X; Zeng Z; Li L; Liu G; Jiang H | NOT A SR (Guidelines, Checklist, Review of review, Descriptive analysis, Review of literature) |
| Dexmedetomidine as a neuraxial adjuvant for prevention of perioperative shivering: Meta-analysis of randomized controlled trials. | Zhang J; Zhang X; Wang H; Zhou H; Tian T; Wu A | NOT OUTCOMES OF INTEREST |
| Foetal responses to dexmedetomidine in parturients undergoing caesarean section: a systematic review and meta-analysis. | Zhang J; Zhou H; Sheng K; Tian T; Wu A | NOT OUTCOMES OF INTEREST |
| Effects of gum chewing on postoperative bowel motility after caesarean section: a meta-analysis of randomised controlled trials. | Zhu YP; Wang WJ; Zhang SL; Dai B; Ye DW | UPADTED/OLD VERSION |
