# Supplementary material for: Evidence-based surgical procedures to optimize caesarean outcomes: an overview of systematic reviews
Source: eClinicalMedicine. 2024 May 19;72:102632. doi: 10.1016/j.eclinm.2024.102632 (PMC11134562; doi:10.1016/j.eclinm.2024.102632)
Supplement: Supplementary Material 4 [file mmc3.docx]

| TABLE 1. REVIEW CHARACTERISTICS OF INCLUDED STUDIES | | | | | | | | | |  |
| --- | --- | --- | --- | --- | --- | --- | --- | --- | --- | --- |
| Review ID | **Assessed as up to date** | **N° of**  **trials** | **N° of participants** | **Includes LMIC?**  **_(Y/N/Unclear)_** | **Type of C-Section _(prelabour /intrapartum /both)_** | **Type of SR: _Cochrane (CR) / Non-Cochrane (NCR)_** | | **AMSTAR Score** | **Title** | |
| Agarwal 2020 | 2019 | 4 | 460 | N | Both | NCR | | Critically Low | Barbed vs conventional suture at cesarean delivery: A systematic review and meta-analysis | |
| Anderson 2004 | 2004 | 7 | 2056 | Y | Both | CR | | Critically Low | Techniques and materials for closure of the abdominal wall in caesarean section | |
| Anorlu 2008 | 2008 | 15 | 4694 | N | Both | CR | | Critically Low | Methods of delivering the placenta at caesarean section | |
| Bamigboye 2014 | 2014 | 21 | 17276 | Y | Both | CR | | Low | Closure versus non-closure of the peritoneum at caesarean section: short- and long-term outcomes | |
| Bhat 2022 | 2022 | 19 | 20739 | Y | Unknown | NCR | | Critically Low | Uterine externalization versus in situ repair of hysterotomy during cesarean delivery: a systematic review, equivalence meta-analysis, and trial sequential analysis | |
| Charoenkwan 2017  _*Only include 1 study 130 women with history of 1 previous caesarean section_ | 2016 | 16 | 2769 | Y | Elective | CR | | High | Scalpel versus electrosurgery for major abdominal incisions | |
| Cornthwaite 2023 | 2023 | 19 | 3057 | Y | Intra-partum | NCR | | Moderate | Management of Impacted Fetal Head at Caesarean Birth | |
| Dumville 2016  _*Only 1 study with 524 wowmen in CS_ | 2016 | 29 | 5879 | N | Unclear | CR | | Moderate | Dressings for the prevention of surgical site infection | |
| Dodd 2014 | 2014 | 27 | 17808 | Y | Both | CR | | Low | Surgical techniques for uterine incision and uterine closure at the time of caesarean section | |
| Eke 2015 | 2015 | 3 | 862 | N | Both | NCR | | Critically Low | Intra-abdominal saline irrigation at cesarean section: a systematic review and meta-analysis review and meta-analysis | |
| Gates 2013 | 2013 | 10 | 5248 | Unclear | Both | CR | | Low | Wound drainage for caesarean section | |
| Hadiati 2020 | 2020 | 13 | 6938 | Y | Both | CR | | High | Skin preparation for preventing infection following caesarean section | |
| Hofmeyr 2008 | 2012  _*Updated search: 15th February 2012 (trials awaiting classification added)_ | 23 | 2950 | Unclear | Unclear | CR | | Low | Techniques for caesarean section | |
| Jacobs 2004 | 2004 | 9 | 1904 | N | Both | CR | | Critically Low | Extra-abdominal versus intra-abdominal repair of the uterine incision at caesarean section | |
| Khanuja 2022 | 2022 | 7 | 9945 | Y | Both | NCR | | Critically Low | Suture type for hysterotomy closure: a systematic review  and meta-analysis of randomized controlled trials | |
| Liabsuetrakul | 2018 | 8 | 2227 | Y | Elective | CR | | High | Mechanical dilatation of the cervix during elective caesarean section before the onset of labour for reducing postoperative morbidity | |
| Mackeen 2012  _*Only 8 studies provided data_ | 2012 | 11 | 1726 | Unclear | Both | CR | | Low | Techniques and materials for skin closure in caesarean section | |
| Mackeen 2022 | 2021 | 14 | 353 | Unclear | Unclear | NCR | | Low | Suture Compared With Staples for Skin Closure After Cesarean Delivery | |
| Mathai 2013 | 2013 | 4 | 666 | Y | Unclear | CR | | Low | Abdominal surgical incisions for caesarean section | |
| Narice 2020 | 2020 | 7 | 1948 | Y | Both | NCR | | Moderate | Impact of changing gloves during cesarean section on postoperative infective complications: A systematic review and meta-analysis | |
| Norman 2017 | 2017 | 20 | 7192 | Y | Both | CR | | High | Intracavity lavage and wound irrigation for prevention of surgical site infection | |
| Norman 2022  _* Only 10 studies enrolled women undergoing caesarean section_ | 2022 | 62 | 1334 | N | Unclear | CR | | High | Negative pressure wound therapy for surgical wounds healing by primary closure | |
| O´Neil 2014 | 2014 | 4 | 581 | N | Elective | NCR | | Critically Low | Omission of the bladder flap at caesarean section reduces delivery time without increased morbidity: a meta-analysis of randomized controlled trials | |
| Pergialiotis 2017 | 2017 | 10 | 3696 | Unclear | Both | NCR | | Critically Low | The impact of subcutaneous tissue suturing at caesarean section on wound complications: a meta-analysis | |
| Pergialiotis 2021 | 2021 | 6 | 2818 | N | Unclear | NCR | | Low | Cephalad-caudad versus transverse blunt expansion of the low transverse hysterotomy during cesarean delivery decreases maternal morbidity: a meta-analysis | |
| Pergialiotis 2022 | 2022 | 19 | 5797 | Unclear | Both | NCR | | Low | Spontaneous versus manual placental delivery during cesarean delivery: a systematic review and meta-analysis | |
| Qayum 2021 | 2021 | 18 | 16312 | Y | Both | NCR | | Critically Low | Single- Versus Double-Layer Uterine Closure After Cesarean Section Delivery: A Systematic Review and Meta-Analysis | |
| Raischer 2022  _*Data extracted only for one outcome coming for sure from RCTs._ | 2021 | 4 | 272 | Unclear | Unclear | NCR | | Critically Low | Knotless Barbed versus Conventional Suture for Closure of the Uterine Incision at Cesarean Delivery: A Systematic Review and Meta-analysis | |
| Rattanakanokchai 2021 | 2020 | 6 | 1707 | Y | Both | NCR | | Critically Low | Changing gloves during cesarean section for prevention of postoperative infections: a systematic review and meta‑analysis | |
| Roberge 2014 | 2013 | 20 | 13086 | Y | Both | NCR | | Critically Low | Impact of single- vs double-layer closure on adverse outcomes and uterine scar defect: a systematic review and meta-analysis | |
| Saad 2014 | 2013 | 2 | 2908 | N | Both | NCR | | Critically Low | Blunt versus sharp uterine incision expansion during low transverse cesarean delivery: a meta-analysis | |
| Sobodu 2024 | 2024 | 4 | 1845 | Y | Both | NCR | | Low | Subcuticular Suture Type at Cesarean Delivery and Infection Risk: A Systematic Review and Meta-Analysis |  |
| Tan 2021 | 2020 | 20 | 20909 | Unclear | Both | NCR | | Critically Low | Uterine exteriorization versus in-situ repair in Cesarean delivery: a systematic review and meta-analysis | |
| Waring 2018 | 2018 | 6 | 1669 | N | Unclear | NCR | | Critically Low | The use of O-ring retractors at Caesarean section A systematic review and meta-analysis | |
| Waterfall 2016 | 2016 | 7 | 582 | Unclear | Both | CR | | Low | Techniques for assisting difficult delivery at caesarean section | |
| Weinstein 2018 | 2018 | 63 | 3143 | Unclear | Elective | CR | | High | Local anaesthetics and regional anaesthesia versus conventional analgesia for preventing persistent postoperative pain in adults and children | |
| Wijetunge 2021 | 2021 | 6 | 2295 | N | Both | NCR | | Low | Advanced dressings for the prevention of surgical site infection in women post-caesarean section: A systematic review and meta-analysis | |
| Zaman 2021 | 2021 | 16 | 4926 | Y | Both | NCR | Critically Low | | Sutures versus clips for skin closure following caesarean section: a systematic review, meta‑analysis and trial sequential analysis of randomised controlled trials | |
