# Supplementary material for: Evidence-based surgical procedures to optimize caesarean outcomes: an overview of systematic reviews
Source: eClinicalMedicine. 2024 May 19;72:102632. doi: 10.1016/j.eclinm.2024.102632 (PMC11134562; doi:10.1016/j.eclinm.2024.102632)
Supplement: Suplementary Material 5 [file mmc4.docx]

| **Intervention** | **Intervention arm** | **Control arm** | **Outcome** | **Effect estimates ***  **(n°studies/n°participants)** | **Certainty of the evidence** | **Reference** | **CATEGORY (CEB, CEH, CEND, PB, PH, IE)*** |
| --- | --- | --- | --- | --- | --- | --- | --- |
| **Skin cleansing** | Parachlorometaxylenol with iodine | Iodine alone | Surgical site infection | 0.33 [0.04,2.99] (1/50) | Very low | Hadiati 2020 | IE |
| **Skin cleansing** | Parachlorometaxylenol with iodine | Iodine alone | Endomyometritis | 0.88 [0.56,1.38] (3/2484) | Very low | Hadiati 2020 | IE |
| **Skin cleansing** | Chlorhexidine gluconate | Povidone iodine | Surgical site infection | 0.72 [0.58,0.91] 8/4323) | Moderate | Hadiati 2020 | CEB |
| **Skin cleansing** | Chlorhexidine gluconate | Povidone iodine | Endometritis | 0.95 [0.49,1.86] (2/2484) | Low | Hadiati 2020 | IE |
| **Skin cleansing** | Chlorhexidine gluconate | Povidone iodine | Adverse events | 0.64 [0.28,1.46] (3/1926) | Very low | Hadiati 2020 | IE |
| **Skin cleansing** | Chlorhexidine plus alcohol | Povidone iodine plus alcohol | Surgical site infection | 0.62 [0.45,0.87] (4/2663) | Low | Hadiati 2020 | PB |
| **Skin cleansing** | Chlorhexidine plus alcohol | Povidone iodine | Surgical site infection | 0.84 [0.61,1.15] (4/1660) | Very low | Hadiati 2020 | IE |
| **Adhesive drape** | Drape | No drape | Surgical site infection | 1.29 [0.97,1.71] (3/1373) | Low | Hadiati 2020 | IE |
| **Adhesive drape** | Drape | No drape | Metritis | 1.62 [0.29, 9.16] (1/79) | Very low | Hadiati 2020 | IE |
| **Adhesive drape** | Drape | No drape | Length of stay (days) | MD  0.10 [-0.27,0.46] (1/603) | Moderate | Hadiati 2020 | IE |
| **Adhesive drape** | Drape: iodine | No drape | Surgical site infection | 1.42 [0.98,2.04] (1/691) | Very low | Hadiati 2020 | IE |
| **Adhesive drape** | Drape: chlorhexidine | No drape | Surgical site infection | 1.11 [0.70,1.76] (1/603) | Moderate | Hadiati 2020 | IE |
| **Skin, subcutaneous tissue, muscle, fascia, incision** | | | | | | | no SR |
| **Abdominal incision** | Joel‐Cohen incision | Pfannenstiel incision | Postoperative febrile morbidity | 0.35 [0.14,0.87] (2/411) | Low | Mathai 2013 | PB |
| **Abdominal incision** | Joel‐Cohen incision | Pfannenstiel incision | Estimated blood loss (mL) | MD  ‐58.0 [‐108.51,‐7.49] (1/101) | Low | Mathai 2013 | PB |
| **Abdominal incision** | Joel‐Cohen incision | Pfannenstiel incision | Postoperative analgesia on demand | 0.55 [0.40,0.76] (1/101) | Low | Mathai 2013 | PB |
| **Abdominal incision** | Joel‐Cohen incision | Pfannenstiel incision | Total dose of analgesics in 24 hours | MD  ‐0.89 [‐1.19,‐0.59] (1/101) | Low | Mathai 2013 | PB |
| **Abdominal incision** | Joel‐Cohen incision | Pfannenstiel incision | Postoperative hospital stay for mother (days) | MD  ‐1.5 [‐2.16,‐0.84] (1/101) | Low | Mathai 2013 | PB |
| **Abdominal incision** | Joel‐Cohen incision | Pfannenstiel incision | Total operative time (minutes) | MD  ‐11.40 [‐16.55,‐6.25] (1/101) | Low | Mathai 2013 | PB |
| **Abdominal incision** | Joel‐Cohen incision | Pfannenstiel incision | Wound infection as defined by trial authors | 1.56 [0.45, 5.42] (1/310) | Very low | Mathai 2013 | IE |
| **Abdominal incision** | Joel‐Cohen incision | Pfannenstiel incision | Blood transfusion | N/E (1/310) | Very low | Mathai 2013 | IE |
| **Abdominal incision** | Joel‐Cohen incision | Pfannenstiel incision | Time (hours) from surgery to start of breastfeeding | MD  ‐5.5 [‐13.62,2.62] (1/101) | Very low | Mathai 2013 | IE |
| **Abdominal incision** | Joel‐Cohen incision | Pfannenstiel incision | Need for re‐laparotomy | N/E (1/310) | Very low | Mathai 2013 | IE |
| **Abdominal incision** | Joel‐Cohen incision | Pfannenstiel incision | Admissions to special care baby unit | 1.19 [0.44, 3.20] (1/310) | Very low | Mathai 2013 | IE |
| **Abdominal incision** | Joel‐Cohen incision | Pfannenstiel incision | Stay in special care nursery (days) | MD  ‐0.46 [‐0.95,0.03] (1/101) | Very low | Mathai 2013 | IE |
| **Abdominal incision** | Joel‐Cohen-based incision | Pfannenstiel incision | Fever treated with antibiotics or as defined by trial authors | 0.47[0.28,0.81] (8/1412) | High | Hofmeyr 2008 | CEB |
| **Abdominal incision** | Joel‐Cohen-based incision | Pfannenstiel incision | Blood loss | MD  -64.45[-91.34, -37.56] (4/481) | High | Hofmeyr 2008 | CEB |
| **Abdominal incision** | Joel‐Cohen-based incision | Pfannenstiel incision | Time to oral intake (hours) | MD  -3.92[-7.13, -0.71] (4/481) | Moderate | Hofmeyr 2008 | CEB |
| **Abdominal incision** | Joel‐Cohen-based incision | Pfannenstiel incision | Length of postoperative stay for mother (days) | MD  -0.99 [-1.44, -0.54] (3/323) | Moderate | Hofmeyr 2008 | CEB |
| **Abdominal incision** | Joel‐Cohen-based incision | Pfannenstiel incision | Operating time (minutes) | MD  ‐18.65[-24.84, -12.45] (4/481) | Moderate | Hofmeyr 2008 | CEB |
| **Abdominal incision** | Joel‐Cohen-based incision | Pfannenstiel incision | Time from skin incision to delivery (minutes) | MD  -3.84[-5.41, -2.27] (4/575) | Moderate | Hofmeyr 2008 | CEB |
| **Abdominal incision** | Joel‐Cohen-based incision | Pfannenstiel incision | Postoperative pain as measured by trial authors | MD  -14.18 [-18.31, -10.04] (1/172) | Very low | Hofmeyr 2008 | IE |
| **Abdominal incision** | Joel‐Cohen-based incision | Pfannenstiel incision | Endometritis | 0.34 [0.01,8.17] (3/767) | Low | Hofmeyr 2008 | IE |
| **Abdominal incision** | Joel‐Cohen-based incision | Pfannenstiel incision | Blood transfusion | 4.08 [0.46,36.4] (3/681) | Low | Hofmeyr 2008 | IE |
| **Abdominal incision** | Joel‐Cohen-based incision | Pfannenstiel incision | Wound infection | 1.43[0.52,3.91] (6/1071) | Moderate | Hofmeyr 2008 | IE |
| **Abdominal incision** | Joel‐Cohen-based incision | Pfannenstiel incision | Time to mobilisation (hours) | MD  -2.86[-11.29,5.56] (2/208) | Low | Hofmeyr 2008 | IE |
| **Abdominal incision** | Joel‐Cohen-based incision | Pfannenstiel incision | Serious complications (organ damage, blood transfusion, significant sepsis, thromboembolism, organ failure, high care unit admission or death) | 1.31[0.29,5.91] (4/913) | Low | Hofmeyr 2008 | IE |
| **Abdominal incision** | Joel‐Cohen-based incision | Pfannenstiel incision | Apgar score < 7 at 5 minutes | 0.18 [0.01,3.71] (1/158) | Low | Hofmeyr 2008 | IE |
| **Abdominal incision** | Joel‐Cohen-based incision | Pfannenstiel incision | Neonatal intensive care admission | 1.19 [0.44,3.2] (1/310) | Low | Hofmeyr 2008 | IE |
| **Abdominal incision** | Joel-Cohen based (Modified Misgav-Ladach) | Traditional (lower midline incision) | Blood loss (mL) | MD  ‐93[-132.72, -53.28] (1/339) | Moderate | Hofmeyr 2008 | CEB |
| **Abdominal incision** | Joel-Cohen based (Modified Misgav-Ladach) | Traditional (lower midline incision) | Operating time (min) | MD  -7.3[-8.32, -6.28] (1/339) | Moderate | Hofmeyr 2008 | CEB |
| **Abdominal incision** | Joel-Cohen based (Modified Misgav-Ladach) | Traditional (lower midline incision) | Time to mobilisation (h) | MD  -16.06[-18.22, -13.9] (1/339) | Moderate | Hofmeyr 2008 | CEB |
| **Abdominal incision** | Joel-Cohen based (Modified Misgav-Ladach) | Traditional (lower midline incision) | Length of postoperative hospital stay for mother (days) | MD  -0.82[-1.08, -0.56] (1/339) | Moderate | Hofmeyr 2008 | CEB |
| **Abdominal incision** | Joel-Cohen based (Modified Misgav-Ladach) | Traditional (lower midline incision) | Fever treated with antibiotics or as defined by trialists | 1.38 [0.75,2.54] (1/339) | Low | Hofmeyr 2008 | IE |
| **Abdominal incision** | Joel-Cohen based (Modified Misgav-Ladach) | Traditional (lower midline incision) | Endometritis | 2.5[0.49,12.74] (1/400) | Very low | Hofmeyr 2008 | IE |
| **Abdominal incision** | Joel-Cohen based (Modified Misgav-Ladach) | Traditional (lower midline incision) | Wound infection | 1.14[0.68,1.91] (1/339) | Moderate | Hofmeyr 2008 | IE |
| **Abdominal incision** | Joel-Cohen based (Modified Misgav-Ladach) | Traditional (lower midline incision) | Postoperative anaemia | 0.6[0.22,1.62] (1/339) | Low | Hofmeyr 2008 | IE |
| **Abdominal incision** | Misgav-Ladach | Modified Misgav-Ladach (all trials) | Time from skin incision to delivery (min) | MD  2.1[1.1,3.1] (1/116) | Very low | Hofmeyr 2008 | IE |
| **Abdominal incision** | Misgav-Ladach | Modified Misgav-Ladach (all trials) | Blood loss | 14[-0.7,28.7] (1/116) | Very low | Hofmeyr 2008 | IE |
| **Abdominal incision** | Misgav-Ladach | Modified Misgav-Ladach (all trials) | Operating time (min) | MD  -0.8[-2.98,1.38] (1/116) | Very low | Hofmeyr 2008 | IE |
| **Abdominal incision** | Misgav-Ladach | Modified Misgav-Ladach (all trials) | Time to oral intake | 1.4[-1.28,4.08] (1/116) | Very low | Hofmeyr 2008 | IE |
| **Abdominal incision** | Misgav-Ladach | Modified Misgav-Ladach (all trials) | Length of postoperative hospital stay for mother (day) | 0.1[-0.99,1.19] (1/116) | Very low | Hofmeyr 2008 | IE |
| **Abdominal incision** | Misgav-Ladach | Modified Misgav-Ladach (all trials) | Postoperative pain as measured by trial authors | 2.3[-0.97,5.57] (1/116) | Very low | Hofmeyr 2008 | IE |
| **Abdominal incision** | Muscle‐cutting/ Maylard incision | Pfannenstiel incision | Postoperative febrile morbidity | 1.26 [0.08,19.50] (1/97) | Very low | Mathai 2013 | IE |
| **Abdominal incision** | Muscle‐cutting/ Maylard incision | Pfannenstiel incision | Wound infection as defined by trial authors | 1.26 [0.27,5.91] (1/97) | Very low | Mathai 2013 | IE |
| **Abdominal incision** | Muscle‐cutting/ Maylard incision | Pfannenstiel incision | Blood transfusion | 0.42 [0.02,9.98] (1/97) | Very low | Mathai 2013 | IE |
| **Abdominal incision** | Muscle‐cutting/ Maylard incision | Pfannenstiel incision | Postoperative hospital stay for mother (days) | MD  0.4 [-0.34,1.14] (1/97) | Very low | Mathai 2013 | IE |
| **Abdominal incision** | Muscle‐cutting/ Maylard incision | Pfannenstiel incision | Long‐term complication ‐ physical test at 3 months (Janda's test) | 0.1[-0.73,0.93] (1/54) | Very low | Mathai 2013 | IE |
| **Abdominal incision** | Extraperitoneal | Intraperitoneal caesarean section | Serious complication | 0.12[0.02,0.88] (1/412) | Moderate | Hofmeyr 2008 | CEB |
| **Abdominal incision** | Extraperitoneal | Intraperitoneal caesarean section | Fever treated with antibiotics or as defined by trialists | 0.42[0.27,0.65] (1/412) | High | Hofmeyr 2008 | CEB |
| **Abdominal incision** | Extraperitoneal | Intraperitoneal caesarean section | Maternal mortality | 0.17[0.02,1.37] (1/412) | Low | Hofmeyr 2008 | IE |
| **Abdominal incision** | Extraperitoneal | Intraperitoneal caesarean section | Repeat operative procedures on the wound | 1.5[0.7,3.2] (1/412) | Low | Hofmeyr 2008 | IE |
| **Type of scalpel used** | Scalpel | Electrosurgery | Wound infection | 1.07[0.74,1.54] (11/2178) | Very Low | Charoenkwan 2017 | IE |
| **Type of scalpel used** | Scalpel | Electrosurgery | Wound dehiscence | 1.21[0.58,2.5] (6/1064) | Very Low | Charoenkwan 2017 | IE |
| **Bladder flap** | Bladder flap formation | Bladder flap non formation | Skin incision to delivery time (min) | WMD  1.27 (0.63, 1.92) (3/466) | Very Low | O´Neil 2014 | IE |
| **Bladder flap** | Bladder flap formation | Bladder flap non formation | Blood loss (ml) | WMD  42.4 (-32.3, 117) (3/479) | Very Low | O´Neil 2014 | IE |
| **Bladder flap** | Bladder flap formation | Bladder flap non formation | Bladder injury | OR  0.96 (0.19 to 4.84) (3/479) | Very Low | O´Neil 2014 | IE |
| **Bladder flap** | Bladder flap formation | Bladder flap non formation | Total surgical time (min) | WMD  3.5 (-0.19, 7.16) (4/581) | Very Low | O´Neil 2014 | IE |
| **Bladder flap** | Bladder flap formation | Bladder flap non formation | Duration of hospitalization (days) | WMD  0.07 (-0.50, 0.64) (2/364) | Very Low | O´Neil 2014 | IE |
| **Use of surgical tapes** | |  | - | - | - | - | no SR |
| **Use of aspiration device** | | - | - | - | - | - | no SR |
| **Use of retractors** | O´ring retractor | Control (standard care) | SSI BMI<35 | 0.34 [0.12, 0.98] (2/264) | Low | Waring 2018 | PB |
| **Use of retractors** | O´ring retractor | Control (standard care) | SSI | 0.76 [0.34,1.79] (5/1515) | Low | Waring 2018 | IE |
| **Use of retractors** | O´ring retractor | Control (standard care) | SSI BMI >35 | 1.04 [0.67, 1.64] (2/483) | Very low | Waring 2018 | IE |
| **Use of retractors** | O´ring retractor | Control (standard care) | Need for uterine exteriorization | 0.48 [0.33, 0.69] (4/1301) | Moderate | Waring 2018 | CEB |
| **Use of retractors** | O´ring retractor | Control (standard care) | Adequate visualisation achieved | 1.01 [1.04, 1.16] (2/352) | Moderate | Waring 2018 | CEB |
| **Uterine incision** | Auto stapler | Conventional incision | Febrile morbidity | 0.92 [0.38, 2.20] (2/300) | Very Low | Dodd 2014 | IE |
| **Uterine incision** | Auto stapler | Conventional incision | Endometritis | 0.2 [0.02, 1.65] (1/100) | Very Low | Dodd 2014 | IE |
| **Uterine incision** | Auto stapler | Conventional incision | Need for blood transfusion | 1.5 [0.26, 8.60] (1/100) | Very Low | Dodd 2014 | IE |
| **Uterine incision** | Auto stapler | Conventional incision | Mean blood loss (ml) | MD  ‐87.0 [‐175.09, 1.09] (1/200) | Very Low | Dodd 2014 | IE |
| **Uterine incision** | Auto stapler | Conventional incision | Duration of surgery (min) | 3.30 [‐0.02, 6.62] (1/197) | Very Low | Dodd 2014 | IE |
| **Uterine incision** | Auto stapler | Conventional incision | Hospital stay (Duration of postnatal stay)(days) | MD  0.0 [‐0.28, 0.28] (1/200) | Very Low | Dodd 2014 | IE |
| **Uterine incision** | Auto stapler | Conventional incision | Wound complications | 1.5 [0.67,3.35] (1/100) | Very Low | Dodd 2014 | IE |
| **Uterine incision** | Blunt dissection/expansion | Sharp dissection/expansion | Mean blood loss (ml) | MD  ‐55.00 [‐79.48, ‐30.52] (2/1145) | Moderate | Dodd 2014 | CEB |
| **Uterine incision** | Blunt dissection/expansion | Sharp dissection/expansion | Need for blood transfusion | 0.24 [0.09, 0.62] (2/1345) | Low | Dodd 2014 | PB |
| **Uterine incision** | Blunt dissection/expansion | Sharp dissection/expansion | Unintended incision extension | 0.47 (0.28, 0.79) (5/2608) | Very Low | Saad 2014 | IE |
| **Uterine incision** | Blunt dissection/expansion | Sharp dissection/expansion | Operative time (min) | MD  -2.06 (-2.11, -2.01) (/1276) | High | Saad 2014 | CEB |
| **Uterine incision** | Blunt dissection/expansion | Sharp dissection/expansion | Febrile morbidity (including endometritis) | 0.86 [0.70,1.05] (4/1941) | Low | Dodd 2014 | IE |
| **Uterine incision** | Blunt dissection/expansion | Sharp dissection/expansion | Maternal death or serious morbidity | 3.0 [0.12, 73.20] (1/400) | Very Low | Dodd 2014 | IE |
| **Uterine incision** | Cephalad-caudad expansion | **Control (transversal expansion)** | Unintended incision extension | MD  0.62 [0.45, 0.86] (6/2818) | Moderate | Pergialiotis 2021 | CEB |
| **Uterine incision** | Transverse blunt extension | **Cephalad‐caudad blunt extension** | Mean blood loss (ml) | 42.0 [1.31, 82.69] (1/811) | Moderate | Dodd 2014 | CEH |
| **Uterine incision** | Transverse blunt extension | **Cephalad‐caudad blunt extension** | Need for blood transfusion | 0.75 [0.28, 2.03] (4/2208) | Very Low | Pergialiotis 2021 | IE |
| **Uterine incision** | Transverse blunt extension | **Cephalad‐caudad blunt extension** | Duration of surgery (min) | MD ‐1.50 [‐3.13, 0.13] (1/811) | Very Low | Dodd 2014 | IE |
| **Uterine incision** | Transverse blunt extension | **Cephalad‐caudad blunt extension** | Additional sutures | 0.62 [0.31, 1.23) (4/1869) | Low | Pergialiotis 2021 | IE |
| **Foetal extraction technique** | | | - | - | - | - | no SR |
| **Foetal difficult extraction: Technique** | Tocolysis | Placebo | Maternal side-effects (not prespecified) | NE (1/97) | Low | Waterfall 2016 | IE |
| **Foetal difficult extraction: Technique** | Reverse breech | Head push | Mean blood loss (ml) | MD  -294.92[-493.25, -96.59] (3/298) | Very Low | Waterfall 2016 | IE |
| **Foetal difficult extraction: Technique** | Reverse breech | Head push | Operative time (min) | MD  -14.99 [-27.67, -2.30] (4/357) | Very Low | Waterfall 2016 | IE |
| **Foetal difficult extraction: Technique** | Reverse breech | Head push | Endometritis | 0.52 [0.26, 1.05] (3/285) | Very Low | Waterfall 2016 | IE |
| **Foetal difficult extraction: Technique** | Reverse breech | Head push | Wound infection | 0.96 [0.58, 1.59] (4/357) | Low | Waterfall 2016 | IE |
| **Foetal difficult extraction: Technique** | Reverse breech | Head push | Blood transfusion | 0.57 [0.20, 1.66] (2/177) | Very Low | Waterfall 2016 | IE |
| **Foetal difficult extraction: Technique** | Reverse breech | Head push | Mean hospital stay (not prespecified) (days) | MD  -1.13 [-2.75, 0.48] (3/285) | Very Low | Waterfall 2016 | IE |
| **Foetal difficult extraction: Technique** | Reverse breech | Head push | Infant birth trauma | 1.55 [0.42, 5.73] (3/239) | Very Low | Waterfall 2016 | IE |
| **Foetal difficult extraction: Technique** | Reverse breech | Head push | Admission to neonatal special care or intensive care unit | 0.53 [0.23, 1.22] (2/226) | Very Low | Waterfall 2016 | IE |
| **Foetal difficult extraction: Technique** | Reverse breech | Head push | Early neonatal death rate | 0.54 [0.23, 1.24] (1/108) | Very low | Waterfall 2016 | IE |
| **Foetal difficult extraction: Technique** | Reverse breech | Head push | Average Apgar at 5 minutes (not prespecified) | 0.36 [-0.64, 1.36] (3/239) | Very Low | Waterfall 2016 | IE |
| **Foetal difficult extraction: Technique** | Head push | Reverse breech | Extension of uterine incision | 3.45 [2.41, 4.93] (7/739) | Low | Cornthwaite 2023 | PH |
| **Foetal difficult extraction: Technique** | Elective instrument | Fundal pressure | Infant birth trauma | NE (1/44) | Very Low | Waterfall 2016 | IE |
| **Foetal difficult extraction: Technique** | Elective instrument | Fundal pressure | Extension of uterine incision | 0.7 [0.13, 3.73] (1/44) | Very low | Waterfall 2016 | IE |
| **Foetal difficult extraction: Technique** | Foetal pillow | No foetal pillow | Blood loss >1000ml | 0.19 [0.08, 0.48] (1/240) | Very Low | Cornthwaite 2023 | IE |
| **Foetal difficult extraction: Technique** | Foetal pillow | No foetal pillow | Operative time (min) | MD  -21.20 [-23.20, -19.20] (1/240) | Low | Cornthwaite 2023 | PB |
| **Foetal difficult extraction: Technique** | Foetal pillow | No foetal pillow | Neonatal intensive care admission | 0.62 [0.33, 1.18] (1/240) | Very low | Cornthwaite 2023 | IE |
| **Foetal difficult extraction: Technique** | Foetal pillow | No foetal pillow | Apgar score < 3 at 5 minutes | 0.43 [0.04,4.33] (4/414) | Very low | Cornthwaite 2023 | IE |
| **Foetal difficult extraction: Technique** | Foetal pillow | No foetal pillow | Incision to delivery interval | MD  -120.7 [−126.2, −115.2] (1/240) | Low | Cornthwaite 2023 | PB |
| **Foetal difficult extraction: Technique** | Inflated foetal pillow | Non-inflated foetal pillow | Uterine incision extension | 0.46 [0.2, 1.05] (1/60) | Low | Cornthwaite 2023 | IE |
| **Foetal difficult extraction: Technique** | Inflated foetal pillow | Non-inflated foetal pillow | Blood transfusion | OR  0.13 [0.01, 1.26] (1/60) | Low | Cornthwaite 2023 | IE |
| **Foetal difficult extraction: Technique** | Inflated foetal pillow | Non-inflated foetal pillow | Postpartum pyrexia / sepsis | 1.2 [0.41, 3.51] (1/60) | Low | Cornthwaite 2023 | IE |
| **Techniques for foetal extraction** | | | | | | | no SR |
| **Placental extraction** | Manual placental removal | Cord traction/Spontaneous delivery | Endometritis | 1.64 [1.42, 1.9] (13/4134) | High | Anorlu 2008 | CEH |
| **Placental extraction** | Manual placental removal | Cord traction/Spontaneous delivery | Length of postoperative hospital stay for the mother (days) | MD  0.39 [0.17,0.61] (3/546) | High | Anorlu 2008 | CEH |
| **Placental extraction** | Manual placental removal | Cord traction/Spontaneous delivery | Puerperal fever | 1.14 [0.63,2.08] (2/580) | Moderate | Anorlu 2008 | IE |
| **Placental extraction** | Manual placental removal | Cord traction/Spontaneous delivery | Blood loss | MD  149.18 [-32.55,330.92] (11/2678) | Low | Pergialiotis 2022 | IE |
| **Placental extraction** | Manual placental removal | Cord traction/Spontaneous delivery | Intraoperative duration | MD  -0.89 [-2.34,0.57] (12/2985) | Very low | Pergialiotis 2022 | IE |
| **Placental extraction** | Manual placental removal | Cord traction/Spontaneous delivery | Haemorrhage (fixed effect) | 1.83 [1.20,2.78] (3/1156) | High | Pergialiotis 2022 | CEH |
| **Placental extraction** | Manual placental removal | Cord traction/Spontaneous delivery | Blood transfusion | 1.20 [0.63,2.28] (7/2508) | Moderate | Pergialiotis 2022 | CEND |
| **Placental extraction** | Manual placental removal | Cord traction/Spontaneous delivery | Infectious morbidity | OR  1.82 [0.94,3.52] (10/3359) | Low | Pergialiotis 2022 | IE |
| **Timing for umbilical cord clamping** | | |  |  |  |  | no SR |
| **Uterine cleaning** | |  | - | - | - | - | no SR |
| **Uterine closure** | Single layer uterine closure | Double layer uterine closure | Postoperative febrile morbidity (including endometritis) | 0.98 [0.85, 1.12] (9/13890) | High | Dodd 2014 | CEND |
| **Uterine closure** | Single layer uterine closure | Double layer uterine closure | Wound infection | 0.99 [0.89, 1.10] (5/13389) | High | Dodd 2014 | CEND |
| **Uterine closure** | Single layer uterine closure | Double layer uterine closure | Maternal infectious morbidity | 0.94 [0.66,1.34] (6/4844) | Moderate | Qayum 2021 | IE |
| **Uterine closure** | Single layer uterine closure | Double layer uterine closure | Endometritis | 1.04 (0.81, 1.34) (8/13815) | Low | Roberge 2014 | IE |
| **Uterine closure** | Single layer uterine closure | Double layer uterine closure | Blood loss | MD  7.14 [-16.21,30.50] (9/3106) | Low | Qayum 2021 | IE |
| **Uterine closure** | Single layer uterine closure | Double layer uterine closure | Blood loss greater than 500 mL | 0.70 [0.42, 1.18] (1/339) | Low | Dodd 2014 | IE |
| **Uterine closure** | Single layer uterine closure | Double layer uterine closure | Need for blood transfusion | 0.86 [0.63, 1.17] (4/13571) | Moderate | Dodd 2014 | IE |
| **Uterine closure** | Single layer uterine closure | Double layer uterine closure | Duration of caesarean (min) | MD  -2.25 [-3.29, -1.21] (10/6598) | Moderate | Qayum 2021 | CEB |
| **Uterine closure** | Single layer uterine closure | Double layer uterine closure | Hospital stay | MD  -0.12 [-0.30,0.06] (6/5774) | Low | Qayum 2021 | IE |
| **Uterine closure** | Single layer uterine closure | Double layer uterine closure | Operative procedure on wound | 0.80 [0.53, 1.21] (3/12604) | Moderate | Dodd 2014 | IE |
| **Uterine closure** | Single layer uterine closure | Double layer uterine closure | Complications post‐op requiring re‐laparotomy | 0.85 [0.63, 1.16] (1/9286) | Moderate | Dodd 2014 | IE |
| **Uterine closure** | Single layer uterine closure | Double layer uterine closure | Readmission rate | 0.95 [0.64,1.40] (2/5007) | Low | Qayum 2021 | IE |
| **Uterine closure** | Single layer uterine closure | Double layer uterine closure | Complication of future pregnancy | 3.21 [0.13, 77.55] (1/145) | Very Low | Dodd 2014 | IE |
| **Uterine closure** | Single layer uterine closure | Double layer uterine closure | Uterine dehiscence | 1.88 [0.63,5.62] (3/2379) | Low | Qayum 2021 | IE |
| **Uterine closure** | Single layer uterine closure | Double layer uterine closure | Postoperative pain present | 0.88 [0.54, 1.42] (2/9444) | Low | Dodd 2014 | IE |
| **Uterine closure** | Single layer uterine closure | Double layer uterine closure | Dysmenorrhea | 1.36 [1.02,1,81] (4/7847) | High | Qayum 2021 | CEH |
| **Uterine closure** | Single layer uterine closure | Double layer uterine closure | Death or serious maternal morbidity | 1.04 [0.71, 1.54] (3/12665) | Moderate | Dodd 2014 | IE |
| **Uterine closure** | Locked sutures: double-layer suture with the first layer locked | Unlocked sutures: double-layer suture with unlocked first layer | Risk of uterine scar dehiscence at next caesarean delivery | 2.14 [ 0.22, 21.10] (1/29) | Very Low | Roberge 2014 | IE |
| **Uterine closure** | Multifilament suture | Monofilament suture | Endometritis | Not estimable (1/95) | Very Low | Khanuja 2022 | IE |
| **Uterine closure** | Multifilament suture | Monofilament suture | Wound infection | Not estimable (1/95) | Very Low | Khanuja 2022 | IE |
| **Uterine closure** | Multifilament suture | Monofilament suture | Maternal infection | Not estimable (1/95) | Very Low | Khanuja 2022 | IE |
| **Uterine closure** | Multifilament suture | Monofilament suture | Postpartum haemorrhage | 7.09 [0.4,136.2] (3/489) | Very Low | Khanuja 2022 | IE |
| **Uterine closure** | Multifilament suture | Monofilament suture | Blood transfusion | Not estimable (2/189) | Very Low | Khanuja 2022 | IE |
| **Uterine closure** | Multifilament suture | Monofilament suture | Uterine rupture | Not estimable (1/95) | Very Low | Khanuja 2022 | IE |
| **Uterine closure** | Multifilament suture | Monofilament suture | Uterine dehiscence | Not estimable (1/95) | Very Low | Khanuja 2022 | IE |
| **Uterine closure** | Chromic suture | Multifilament suture | Endometritis | 0.83 [0.49,1.40] (1/9184) | Moderate | Khanuja 2022 | IE |
| **Uterine closure** | Chromic suture | Multifilament suture | Wound infection | 1.07 [0.84,1.36] (1/9184) | Moderate | Khanuja 2022 | IE |
| **Uterine closure** | Chromic suture | Multifilament suture | Maternal infection | 0.33 [0.02,6.52] (1/9184) | Moderate | Khanuja 2022 | IE |
| **Uterine closure** | Chromic suture | Multifilament suture | Use of additional uterotonics | 0.50 [0.22,1.13] (1/9184) | Moderate | Khanuja 2022 | IE |
| **Uterine closure** | Chromic suture | Multifilament suture | Blood transfusion | 0.53 [0.30,0.93] (1/9184) | High | Khanuja 2022 | CEB |
| **Uterine closure** | Chromic suture | Multifilament suture | Uterine rupture | 3.05 [0.32,29.29] (1/3306) | Moderate | Khanuja 2022 | IE |
| **Uterine closure** | Chromic suture | Multifilament suture | Uterine dehiscence | 0.67 [0.11,4.02] (1/3306) | Moderate | Khanuja 2022 | IE |
| **Uterine closure** | Chromic suture | Multifilament suture | Operation time (min) | Not reported | Moderate | Khanuja 2022 | IE |
| **Uterine closure** | Chromic catgut | Polygactin‐910 | Postoperative febrile morbidity (including endometritis) | 0.70 [0.49, 1.00] (1/9544) | Moderate | Dodd 2014 | PB |
| **Uterine closure** | Chromic catgut | Polygactin‐910 | Complications post‐op requiring re‐laparotomy | 0.58 [0.37, 0.89] (1/9544) | High | Dodd 2014 | CEB |
| **Uterine closure** | Chromic catgut | Polygactin‐910 | Operative procedure on wound | 0.64 [0.36, 1.13] (1/9544) | Moderate | Dodd 2014 | IE |
| **Uterine closure** | Chromic catgut | Polygactin‐910 | Maternal readmission | 1.00 [0.58, 1.72] (1/9544) | Moderate | Dodd 2014 | IE |
| **Uterine closure** | Chromic catgut | Polygactin‐910 | Postoperative pain present | 0.86 [0.70, 1.07] (1/9544) | Moderate | Dodd 2014 | IE |
| **Uterine closure** | Chromic catgut | Polygactin‐910 | Death or serious maternal morbidity | 0.68 [0.44, 1.06] (1/9544) | Moderate | Dodd 2014 | IE |
| **Uterine closure** | Multifilament suture | Barbed suture | Endometritis | Not estimable (2/172) | Very Low | Khanuja 2022 | IE |
| **Uterine closure** | Multifilament suture | Barbed suture | Wound infection | Not estimable (2/172) | Very Low | Khanuja 2022 | IE |
| **Uterine closure** | Multifilament suture | Barbed suture | Maternal infection | 0.3 [0.01,7.9] (2/202) | Low | Khanuja 2022 | IE |
| **Uterine closure** | Multifilament suture | Barbed suture | Estimated blood loss (mL) | MD  46.17 [-13.55,105.89] (3/272) | Very low | Khanuja 2022 | IE |
| **Uterine closure** | Multifilament suture | Barbed suture | Use of additional uterotonics | 0.9 [0.47,1.79] (1/70) | Very Low | Khanuja 2022 | IE |
| **Uterine closure** | Multifilament suture | Barbed suture | Blood transfusion | 3.0 [0.3,28.3] (2/136) | Low | Khanuja 2022 | IE |
| **Uterine closure** | Multifilament suture | Barbed suture | Uterine rupture | Not estimable (1/102) | Very low | Khanuja 2022 | IE |
| **Uterine closure** | Multifilament suture | Barbed suture | Uterine dehiscence | Not estimable (1/102) | Very low | Khanuja 2022 | IE |
| **Uterine closure** | Barbed suture | Conventional suture | Combined postoperative morbidity | 0.96 (0.46, 2.00) (3/272) | Very low | Agarwal 2020 | IE |
| **Uterine closure** | Multifilament suture | Barbed suture | Uterine repair time (min) | MD  1.8 [1.6,2.1] (3/272) | Moderate | Khanuja 2022 | CEH |
| **Uterine closure** | Multifilament suture | Barbed suture | Operation time (min) | MD  1.9 [0.03, 3.8] (3/272) | Moderate | Khanuja 2022 | CEH |
| **Uterine closure** | Barbed suture | Conventional suture | Need for additional haemostatic sutures | 0.39 (0.28, 0.54) (3/272) | Low | Agarwal 2020 | PB |
| **Uterine closure** | Uterine exteriorization repair | Intraperitoneal repair at caesarean section | Febrile morbidity for more than 3 days | 0.41[0.17,0.97] (1/308) | Low | Jacobs 2004 | PB |
| **Uterine closure** | Uterine exteriorization repair | Intraperitoneal uterine repair | Intraoperative nausea and vomiting | OR  2.09 (1.66, 2.63) (4/1454) | Moderate | Tan 2021 | CEH |
| **Uterine closure** | Uterine exteriorization repair | Intraperitoneal uterine repair | Intraoperative nausea | OR  1.08 [0.82, 1.42] (7/2,343) | Moderate | Tan 2021 | IE |
| **Uterine closure** | Uterine exteriorization repair at caesarean section | Intraperitoneal repair | Intraoperative vomiting | OR  1.94 [0.69, 1.28] (6, 2634) | Low | Baht 2022 | IE |
| **Uterine closure** | Uterine exteriorization repair | Intraperitoneal | Pain at 6 hs | OR  1.64 [1.31, 2.03] (2/1637) | High | Tan 2021 | CEH |
| **Uterine closure** | Uterine exteriorization repair | Intraperitoneal | Satisfaction with operation | 0.92 [0.82,1.04] (1/139) | Low | Jacobs 2004 | PEND |
| **Uterine closure** | Uterine exteriorization repair | Intraperitoneal | Endometritis | 1.22 (0.96, 1.55) (11/18339) | Low | Tan 2021 | IE |
| **Uterine closure** | Uterine exteriorization repair | Intraperitoneal | Sepsis | 0.94 (0.19,4.57) 1/308) | Low | Jacobs 2004 | IE |
| **Uterine closure** | Uterine exteriorization repair | Intraperitoneal | Wound infection | 1.01 (0.71, 1.45) (11/18662) | Low | Tan 2021 | IE |
| **Uterine closure** | Uterine exteriorization repair | Intraperitoneal | Operative blood loss (ml) | MD  17.11 (-23.15,57.37) (6/504) | Moderate | Jacobs 2004 | IE |
| **Uterine closure** | Uterine exteriorization repair | Intraperitoneal | Blood transfusion | OR 1.11 [0.63, 1.94]  (10/18429) | Moderate | Tan 2021 | IE |
| **Uterine closure** | Uterine exteriorization repair | Intraperitoneal | Estimated blood loss (mL) | MD  -40.8 [-90.42, 8.82] (9/2208) | Low | Tan 2021 | IE |
| **Uterine closure** | Uterine exteriorization repair | Intraperitoneal | Pain (intra-operative) | 1.76 [0.97, 3.20] (5/704) | Moderate | Tan 2021 | IE |
| **Uterine closure** | Intraperitoneal | Uterine exteriorization repair | Rescue analgesia | OR  0.44 [0.28, 0.68] (6/17591) | Moderate | Baht 2022 | CEB |
| **Uterine closure** | Uterine exteriorization repair | Intraperitoneal | Nausea | 1.18 [0.78,1.8] (3/667) | Low | Jacobs 2004 | IE |
| **Uterine closure** | Uterine exteriorization repair | Intraperitoneal | Postoperative nausea | OR  1.36 [0.86, 2.14] (3/1975) | Moderate | Tan 2021 | IE |
| **Uterine closure** | Uterine exteriorization repair | Intraperitoneal | Duration of operation (min) | MD  1.70 [- 0.72, 4.12] (16/19399) | Very low | Tan 2021 | IE |
| **Uterine closure** | Uterine exteriorization repair | Intraperitoneal | Length of stay (days) | MD  0.16 [- 0.08, 0.41] (10/17340) | Low | Tan 2021 | IE |
| **Uterine closure** | Uterine exteriorization repair at caesarean section | Intraperitoneal | Hypotension | OR  1.42 [0.90, 2.22] (6/2573) | Low | Baht 2022 | IE |
| **Uterine closure** | Intraperitoneal | Uterine exteriorization repair | Return to bowel function | MD  −0.76 [−1.36, −0.15] (6/7696) | Moderate | Baht 2022 | CEB |
| **Abdominal irrigation** | Irrigation (any type) | No irrigation | SSI | 0.87 [0.68, 1.11] (14/6106) | Low | Norman 2017 | IE |
| **Abdominal irrigation** | Irrigation (any type) | No irrigation | Abscess | 0.91 [0.54, 1.54] (3/331) | Moderate | Norman 2017 | IE |
| **Abdominal irrigation** | Irrigation (any type) | No irrigation | Mortality | 0.86 [0.36, 2.04] (2/280) | Very Low | Norman 2017 | IE |
| **Abdominal irrigation** | Irrigation (any type) | No irrigation | Hospital stay (days) | MD  ‐0.13 [‐0.38, 0.12] (7/1597) | Very Low | Norman 2017 | IE |
| **Abdominal irrigation** | Irrigation (any type) | No irrigation | Return to theatre (reoperation) | 0.72 [0.28, 1.84] (2/3247) | Low | Norman 2017 | IE |
| **Abdominal irrigation** | Irrigation (any type) | No irrigation | Readmission to hospital | 0.70 [0.10, 4.90] (2/3247) | Low | Norman 2017 | IE |
| **Abdominal irrigation** | Irrigation (any type) | No irrigation | Adverse events | 1.05 [0.76, 1.44) (3/403) | Low | Norman 2017 | IE |
| **Abdominal irrigation** | Antibacterial irrigation | Non‐antibacterial irrigation | SSI | 0.57 [0.44, 0.75] (30/5141) | Low | Norman 2017 | PB |
| **Abdominal irrigation** | Antibacterial irrigation | Non‐antibacterial irrigation | Hospital stay (days) | MD  ‐0.85 [‐1.60, ‐0.09] (7/635) | Low | Norman 2017 | PB |
| **Abdominal irrigation** | Antibacterial irrigation | Non‐antibacterial irrigation | Mortality | 0.81 [0.48, 1.36] (11/1121) | Very Low | Norman 2017 | IE |
| **Abdominal irrigation** | Antibacterial irrigation | Non‐antibacterial irrigation | Wound dehiscence | 1.26 [0.65, 2.45] (3/660) | Very low | Norman 2017 | IE |
| **Abdominal irrigation** | Antibacterial irrigation | Non‐antibacterial irrigation | Return to theatre (reoperation) | 1.26 [0.12, 13.60] (2/403) | Very Low | Norman 2017 | IE |
| **Abdominal irrigation** | Antibacterial irrigation | Non‐antibacterial irrigation | Adverse events | 0.55 [0.22, 1.34] (3/178) | Low | Norman 2017 | IE |
| **Abdominal irrigation** | Icodextrin | Lactated Ringer's solution | Adverse events | 0.99 [0.96, 1.02] (2/875) | Moderate | Norman 2017 | CEND |
| **Abdominal irrigation** | Icodextrin | Lactated Ringer's solution | Maternal mortality | 0.0 [0.0, 0.0] (2/875) | Very low | Norman 2017 | IE |
| **Abdominal irrigation** | Standard irrigation | Pulsatile irrigation | SSI | 0.34 [0.19, 0.62] ( 2/484) | Low | Norman 2017 | PB |
| **Abdominal irrigation** | Warm normal saline instilled into the abdomen | no treatment | Intraoperative nausea | 1.68 (1.36, 2.06) (2/666) | High | Eke 2015 | CEH |
| **Abdominal irrigation** | Warm normal saline instilled into the abdomen | no treatment | Intraoperative emesis | 1.70 (1.28, 2.25) (2/666) | High | Eke 2015 | CEH |
| **Abdominal irrigation** | Warm normal saline instilled into the abdomen | no treatment | Post-operative use of anti-emetics | 1.84 (1.21, 2.78) (2/666) | High | Eke 2015 | CEH |
| **Abdominal irrigation** | Warm normal saline instilled into the abdomen | no treatment | Post-operative nausea | 1.92 (1.37, 2.69) (2/666) | Low | Eke 2015 | PH |
| **Abdominal irrigation** | Warm normal saline instilled into the abdomen | no treatment | Endometritis | 0.95 (0.64, 1.40) (3/862) | Moderate | Eke 2015 | IE |
| **Abdominal irrigation** | Warm normal saline instilled into the abdomen | no treatment | Wound infection | 0.51 (0.09, 2.73) (2/626) | Low | Eke 2015 | IE |
| **Abdominal irrigation** | Warm normal saline instilled into the abdomen | no treatment | Postoperative Urinary tract infection | 0.92 (0.66, 1.30) (2/626) | Moderate | Eke 2015 | IE |
| **Abdominal irrigation** | Warm normal saline instilled into the abdomen | no treatment | Estimated blood loss (ml) | MD  -8.10 (-20.95, 4.47) (3/862) | Moderate | Eke 2015 | IE |
| **Abdominal irrigation** | Warm normal saline instilled into the abdomen | no treatment | Operative time (min) | MD  0.06 (-5.16, 5.29) (2/666) | Moderate | Eke 2015 | IE |
| **Abdominal irrigation** | Warm normal saline instilled into the abdomen | no treatment | Post-operative emesis | 1.65 (0.74,3.67) (2/666) | Moderate | Eke 2015 | IE |
| **Peritoneum closure** | Non-closure of visceral and parietal peritoneum | Closure of both peritoneal layers | Chronic pelvic pain | 0.49[0.25,0.98] (1/112) | Low | Bamigboye 2014 | PB |
| **Peritoneum closure** | Non-closure of visceral and parietal peritoneum | Closure of both peritoneal layers | Postoperative days in hospital | MD  -0.26[-0.47, -0.05] (13/14906) | Low | Bamigboye 2014 | PB |
| **Peritoneum closure** | Non-closure of visceral and parietal peritoneum | Closure of both peritoneal layers | Operating time (minutes) | MD  -5.81[-7.68, -3.93] (16/15480) | Low | Bamigboye 2014 | PB |
| **Peritoneum closure** | Non-closure of visceral and parietal peritoneum | Closure of both peritoneal layers | Wound infection | 0.96[0.86,1.07] (13/15430 | High | Bamigboye 2014 | CEND |
| **Peritoneum closure** | Non-closure of visceral and parietal peritoneum | Closure of both peritoneal layers | Additional analgesia after 24-48 hours | 0.94[0.79,1.12] (1/9675) | High | Bamigboye 2014 | CEND |
| **Peritoneum closure** | Non-closure of visceral and parietal peritoneum | Closure of both peritoneal layers | Infectious morbidity | 0.92 [0.72,1.16] (11/14985) | Moderate | Bamigboye 2014 | IE |
| **Peritoneum closure** | Non-closure of visceral and parietal peritoneum | Closure of both peritoneal layers | Endometritis | 1.07 [0.78,1.46] (5/10538) | Moderate | Bamigboye 2014 | IE |
| **Peritoneum closure** | Non-closure of visceral and parietal peritoneum | Closure of both peritoneal layers | Blood transfusion > 1 unit (not prespecified outcome) | 0.98 [0.69,1.39] (1/9675) | Moderate | Bamigboye 2014 | IE |
| **Peritoneum closure** | Non-closure of visceral and parietal peritoneum | Closure of both peritoneal layers | Intervention for postpartum haemorrhage (not prespecified outcome) | 0.99[0.72,1.38] (1/9675) | Moderate | Bamigboye 2014 | IE |
| **Peritoneum closure** | Non-closure of visceral and parietal peritoneum | Closure of both peritoneal layers | Numbers of narcotic analgesics required | MD  -0.18[-0.39,0.02](1/1657) | Very Low | Bamigboye 2014 | IE |
| **Peritoneum closure** | Non-closure of visceral and parietal peritoneum | Closure of both peritoneal layers | Pain at 6 weeks postpartum | 1.04 [0.8,1.36] (1/9465) | Moderate | Bamigboye 2014 | IE |
| **Peritoneum closure** | Non-closure of visceral and parietal peritoneum | Closure of both peritoneal layers | Readmission to hospital within 6 weeks (not prespecified outcome) | 1 [0.67,1.49] (1/9465) | Moderate | Bamigboye 2014 | IE |
| **Peritoneum closure** | Non-closure of visceral and parietal peritoneum | Closure of both peritoneal layers | Uterine dehiscence | 0.14 [0.01,2.7] (1/100) | Very Low | Bamigboye 2014 | IE |
| **Peritoneum closure** | Non-closure of visceral and parietal peritoneum | Closure of both peritoneal layers | Maternal death (not prespecified outcome) | 1.49 [0.25,8.92] (1/9675) | Moderate | Bamigboye 2014 | IE |
| **Peritoneum closure** | Non-closure of visceral and parietal peritoneum | Closure of both peritoneal layers | Postoperative adhesions | 0.99 [0.76, 1.29] (4/282) | Low | Bamigboye 2014 | IE |
| **Peritoneum closure** | Non-closure of visceral and parietal peritoneum | Closure of both peritoneal layers | Secondary infertility | 0.89 [0.23,3.44] ( 1/144) | Very low | Bamigboye 2014 | IE |
| **Peritoneum closure** | Non-closure of visceral peritoneum only | Closure of both parietal layers | Wound infection | 0.36 [0.14, 0.89] (2/789) | Very low | Bamigboye 2014 | IE |
| **Peritoneum closure** | Non-closure of visceral peritoneum only | Closure of both parietal layers | Postoperative days in hospital | MD  -0.70 [-0.98, -0.42] (1/549) | Low | Bamigboye 2014 | PB |
| **Peritoneum closure** | Non-closure of visceral peritoneum only | Closure of both parietal layers | Operating time (minutes) | MD  -6.3[-9.22,-3.38] ( 1/544) | Low | Bamigboye 2014 | PB |
| **Peritoneum closure** | Non-closure of visceral peritoneum only | Closure of both parietal layers | Adhesions formation | 2.49 [1.49, 4.16] (2/157) | Very low | Bamigboye 2014 | IE |
| **Peritoneum closure** | Non-closure of visceral peritoneum only | Closure of both parietal layers | Postoperative fever | 0.60 [0.29, 1.27] (3/889) | Very low | Bamigboye 2014 | IE |
| **Peritoneum closure** | Non-closure of visceral peritoneum only | Closure of both parietal layers | Endometritis | 3[0.12,72.91] (1/240) | Very low | Bamigboye 2014 | IE |
| **Peritoneum closure** | Non-closure of parietal peritoneum only | Closure of both parietal layers | Postoperative pain | 0.45[0.31,0.66] (1/325) | Moderate | Bamigboye 2014 | CEB |
| **Peritoneum closure** | Non-closure of parietal peritoneum only | Closure of both parietal layers | Operating time (minutes) | MD  -5.1[-8.71,-1.49] (1/248) | Very low | Bamigboye 2014 | IE |
| **Peritoneum closure** | Non-closure of parietal peritoneum only | Closure of both parietal layers | Mobilisation time in hours (not prespecified outcome) | MD  -1.89[-3.18,-0.6] (1/110) | Moderate | Bamigboye 2014 | CEB |
| **Peritoneum closure** | Non-closure of parietal peritoneum only | Closure of both parietal layers | Time to oral intake in hours (not prespecified outcome) | MD  -2.31[-3.76,-0.86] (1/110) | Moderate | Bamigboye 2014 | CEB |
| **Peritoneum closure** | Non-closure of parietal peritoneum only | Closure of both parietal layers | Postoperative fever | 0.18[0.01,3.56] (1/40) | Low | Bamigboye 2014 | IE |
| **Peritoneum closure** | Non-closure of parietal peritoneum only | Closure of both parietal layers | Endometritis | 0.88[0.53,1.46] (1/248) | Very Low | Bamigboye 2014 | IE |
| **Peritoneum closure** | Non-closure of parietal peritoneum only | Closure of both parietal layers | Wound infection | 0.95[0.14,6.66] (1/248) | Very low | Bamigboye 2014 | IE |
| **Peritoneum closure** | Non-closure of parietal peritoneum only | Closure of both parietal layers | Blood loss (not prespecified outcome) (ml) | MD  56.97[-28.08,142.02] (1/110) | Low | Bamigboye 2014 | IE |
| **Peritoneum closure** | Non-closure of parietal peritoneum only | Closure of both parietal layers | Drop in haemoglobin g/dL (not prespecified outcome) | 0.28[-0.03,0.59] (1/110) | Moderate | Bamigboye 2014 | IE |
| **Peritoneum closure** | Non-closure of parietal peritoneum only | Closure of both parietal layers | Postoperative days in hospital | MD  -0.15[-1.2,0.91] (2/288) | Very Low | Bamigboye 2014 | IE |
| **Peritoneum closure** | Non-closure of parietal peritoneum only | Closure of both parietal layers | Time to flatus (not prespecified outcome) (hours) | MD  -0.04[-1.99,1.91] (1/110) | Very low | Bamigboye 2014 | IE |
| **Peritoneum closure** | Non-closure | closure of visceral peritoneum when parietal peritoneum is closed | Urinary frequency at 8 weeks | 0.24[0.13,0.45] (1/582) | High | Bamigboye 2014 | CEB |
| **Peritoneum closure** | Non-closure | closure of visceral peritoneum when parietal peritoneum is closed | Urgency of urination | 0.3[0.18,0.51] (1/582) | High | Bamigboye 2014 | CEB |
| **Peritoneum closure** | Non-closure | closure of visceral peritoneum when parietal peritoneum is closed | Stress incontinence | 0.45[0.21,0.96] (1/582) | High | Bamigboye 2014 | CEB |
| **Mechanical cervical dilatation** | Manual cervical dilatation | No dilatation | Postpartum haemorrhage (blood loss at least 1000 mL) | 1.97 [0.48, 8.13] (1/47) | Very low | Liabsuetrakul 2018 | IE |
| **Mechanical cervical dilatation** | Manual cervical dilatation | No dilatation | Need for blood transfusion | 3.54 [0.37, 33.79] (2/847) | Very low | Liabsuetrakul 2018 | IE |
| **Mechanical cervical dilatation** | Manual cervical dilatation | No dilatation | Drop from baseline haemoglobin (g/dL) | 0.92 [0.64, 1.31] (2/722) | Low | Liabsuetrakul 2018 | IE |
| **Mechanical cervical dilatation** | Manual cervical dilatation | No dilatation | Secondary postpartum haemorrhage (within 6 weeks) | 1.18 [0.07, 18.76] (1/447) | Very low | Liabsuetrakul 2018 | IE |
| **Mechanical cervical dilatation** | Manual cervical dilatation | No dilatation | Febrile morbidity | 1.18 [0.76, 1.85] (7/2126) | Low | Liabsuetrakul 2018 | IE |
| **Mechanical cervical dilatation** | Manual cervical dilatation | No dilatation | Endometritis | 0.94 [0.35, 2.52] (4/1536) | Very low | Liabsuetrakul 2018 | IE |
| **Mechanical cervical dilatation** | Manual cervical dilatation | No dilatation | Uterine subinvolution | 0.34 [0.08, 1.36] (2/654) | Very low | Liabsuetrakul 2018 | IE |
| **Mechanical cervical dilatation** | Manual cervical dilatation | No dilatation | Blood loss (mL) | MD  -48.49 [-88.75, -8.23] (1/400) | high | Liabsuetrakul 2018 | CEB |
| **Mechanical cervical dilatation** | Manual cervical dilatation | No dilatation | Wound infection | 1.13 [0.44, 2.9] (5/1719) | low | Liabsuetrakul 2018 | IE |
| **Mechanical cervical dilatation** | Manual cervical dilatation | No dilatation | Urinary tract infection | 0.92 [0.34, 2.53](2/847) | low | Liabsuetrakul 2018 | IE |
| **Mechanical cervical dilatation** | Manual cervical dilatation | No dilatation | Infectious morbidity | 0 .91 [0.51, 1.6) (1/400) | High | Liabsuetrakul 2018 | CEB |
| **Mechanical cervical dilatation** | Manual cervical dilatation | No dilatation | Retained products of conception | 0.04 [0.00, 0.63] (1/447) | moderate | Liabsuetrakul 2018 | CEB |
| **Mechanical cervical dilatation** | Manual cervical dilatation | No dilatation | Operative time (min) | MD  -0.05 [-2.62, 2.53] (4/1585) | low | Liabsuetrakul 2018 | IE |
| **Drainage** | Wound drain | No drain | Postoperative analgesia | 0.96 [0.87, 1.07] (1/2796) | High | Gates 2013 | CEND |
| **Drainage** | Wound drain | No drain | Breastfeeding at hospital discharge | 0.98[0.92,1.04] (1/2796) | High | Gates 2013 | CEND |
| **Drainage** | Wound drain | No drain | Febrile morbidity | 0.87[0.66,1.15] ( 6/3829) | Low | Gates 2013 | IE |
| **Drainage** | Wound drain | No drain | Endometritis | 1.2[0.9,1.59] (2/3386) | Moderate | Gates 2013 | IE |
| **Drainage** | Wound drain | No drain | Wound complication | 0.85[0.55,1.32] (6/1640) | Low | Gates 2013 | IE |
| **Drainage** | Wound drain | No drain | Blood loss (ml) | MD  23.41[-1.93,48.74] (2/1030) | Low | Gates 2013 | IE |
| **Drainage** | Wound drain | No drain | Blood transfusion | 1.02[0.7,1.48] (1/2796) | Moderate | Gates 2013 | IE |
| **Drainage** | Wound drain | No drain | Postoperative pain | -0.15[-0.36,0.06] (1/148) | Very low | Gates 2013 | IE |
| **Drainage** | Wound drain | No drain | Operative procedures on wound | 2.4[0.85,6.79] (1/2796) | Low | Gates 2013 | IE |
| **Drainage** | Wound drain | No drain | Readmission to hospital | 1.08 [0.7,1.66] ( 2/ 3064) | Moderate | Gates 2013 | IE |
| **Drainage** | Subcutaneous drain | Sub-sheath drain | Wound infection | 5.42[1.28,22.98] (1/121) | Low | Gates 2013 | PH |
| **Drainage** | Subcutaneous drain | Sub-sheath drain | Febrile morbidity | 1.28[0.7,2.34] (1/121) | Very Low | Gates 2013 | IE |
| **Drainage** | Wound drain | Subcutaneous suture | Endometritis | 1.31[0.74,2.34] (1/385) | Low | Gates 2013 | IE |
| **Drainage** | Wound drain | Subcutaneous suture | Wound infection | 0.77[0.42,1.44] (3/533) | Very low | Gates 2013 | IE |
| **Drainage** | Wound drain | Subcutaneous suture | Wound complications | 0.56[0.17,1.87] (3/533) | Very low | Gates 2013 | IE |
| **Drainage** | Wound drain | Subcutaneous suture | Blood loss (ml) | MD  3[-36.97,42.97] (1/385) | Very low | Gates 2013 | IE |
| **Drainage** | Wound drain | Subcutaneous suture | Postoperative pain | MD  -0.1[-0.36,0.16] (1/98) | Very low | Gates 2013 | IE |
| **Drainage** | Wound drain | Subcutaneous suture | Duration of surgery (mins) | MD  0.3[-3.19,3.79] (1/385) | Very low | Gates 2013 | IE |
| **Drainage** | Wound drain | Subcutaneous suture | Duration of postnatal hospital (days) | MD  0 [-0.3,0.3] (1/385) | Very low | Gates 2013 | IE |
| **Fascial closure** | |  |  |  |  |  | no SR |
| **Subcutaneous closure** | Subcutaneous tissue closure | Non-closure | Seroma formation | 0.53 [0.33, 0.84] ( 8/1979) | High | Pergialotis 2017 | CEB |
| **Subcutaneous closure** | Subcutaneous tissue closure | Non-closure | Any type of wound complications | 0.66 [ 0.47 ,0.93] (10/3811) | High | Pergialotis 2017 | CEB |
| **Subcutaneous closure** | Subcutaneous tissue closure | Non-closure | Wound infection | 1.02 [0.69, 1.50] (5/1348) | Moderate | Anderson 2004 | IE |
| **Subcutaneous closure** | Subcutaneous tissue closure | Non-closure | Endometritis | 0.77 [0.46, 1.28] ( 1/590) | Moderate | Anderson 2004 | IE |
| **Subcutaneous closure** | Subcutaneous tissue closure | Non-closure | Mean blood loss (ml) | MD  9.0 [-24.29, 42.29] (1/590) | Moderate | Anderson 2004 | IE |
| **Subcutaneous closure** | Subcutaneous tissue closure | Non-closure | Haematoma formation | 0.74 [0.22, 2.42] ( 7/1663) | Moderate | Pergialotis 2017 | IE |
| **Subcutaneous closure** | Subcutaneous tissue closure | Non-closure | Duration of surgery (mins) | MD  0.6 (-2.29, 3.49) (1/590) | Moderate | Anderson 2004 | IE |
| **Subcutaneous closure** | Blunt needles for closure at caesarean section | Sharp needles for closure at caesarean section | Wound infection | 2.73 [0.54, 13.76] ( 1/203) | Low | Anderson 2004 | IE |
| **Skin closure** | Staples (all CS) | Absorbable subcuticular suture (all CS) | Skin separation | 3.82 [2.05, 7.12] (5/824) | Moderate | Mackeen 2012 | CEH |
| **Skin closure** | Staples (all CS) | Absorbable subcuticular suture (all CS) | Reclosure | 4.98 [1.82, 13.61] (2/516) | Very Low | Mackeen 2012 | IE |
| **Skin closure** | Staples (all CS) | Absorbable subcuticular suture (all CS) | Maternal length of stay (days) | MD  0.10 [‐0.01, 0.21] ( 1/416) | Moderate | Mackeen 2012 | CEND |
| **Skin closure** | Staples (all CS) | Absorbable subcuticular suture (all CS) | Wound infection | 0.85 [0.43, 1.71] (6/916) | Very Low | Mackeen 2012 | IE |
| **Skin closure** | Staples (all CS) | Absorbable subcuticular suture (all CS) | Wound complications | 1.52 [0.92, 2.52] ( 6/916) | Low | Mackeen 2012 | IE |
| **Skin closure** | Staples (all CS) | Absorbable subcuticular suture (all CS) | Presence of hematoma | 1.32 [0.10, 18.39] (3/283) | Very Low | Mackeen 2012 | IE |
| **Skin closure** | Staples (all CS) | Absorbable subcuticular suture (all CS) | Presence of seroma | 0.32 [0.01, 7.68] ( 2/15) | Low | Mackeen 2012 | IE |
| **Skin closure** | Staples (all CS) | Absorbable subcuticular suture (all CS) | Readmission | 0.56 [0.05, 6.08] (1/416) | Very Low | Mackeen 2012 | IE |
| **Skin closure** | Staples (all CS) | Absorbable subcuticular suture (all CS) | Pain scale at discharge (10 cm scale) | MD  0.57 [‐1.20, 2.33] ( 2/148) | Low | Mackeen 2012 | IE |
| **Skin closure** | Staples (all CS) | Absorbable subcuticular suture (all CS) | Pain scale postpartum (10 cm): 6 weeks | MD  0.59 [‐1.17, 2.36] (2/145) | Low | Mackeen 2012 | IE |
| **Skin closure** | Staples (all CS) | Absorbable subcuticular suture (all CS) | Cosmesis per physician (OSAS) at 2 months | MD  0.0 [‐2.76, 2.76] (1/125) | Very Low | Mackeen 2012 | IE |
| **Skin closure** | Staples (all CS) | Absorbable subcuticular suture (all CS) | Cosmesis per physician (OSAS) at 6 months | MD  1.69 [‐0.44, 3.83]  (2/228) | Low | Mackeen 2012 | IE |
| **Skin closure** | Staples (all CS) | Absorbable subcuticular suture (all CS) | Cosmesis per patient (PSAS) at 2 months | MD  0.20 [‐2.75, 3.15] (1/125) | Very Low | Mackeen 2012 | IE |
| **Skin closure** | Staples (all CS) | Absorbable subcuticular suture (all CS) | Cosmesis per patient (PSAS) at 6 months | MD  0.75 [‐2.08, 3.59] (2/226) | Very Low | Mackeen 2012 | IE |
| **Skin closure** | Staples (all CS) | Absorbable subcuticular suture (all CS) | Patient satisfaction (10 cm scale): at discharge | MD  ‐0.80 [‐1.85, 0.25] (1/98) | Low | Mackeen 2012 | IE |
| **Skin closure** | Staples (all CS) | Absorbable subcuticular suture (all CS) | Patient satisfaction (10 cm scale): 6‐8 weeks postoperatively | MD  0.12 [‐1.24, 1.49] (2/217) | Very Low | Mackeen 2012 | IE |
| **Skin closure** | Staples (all CS) | Absorbable subcuticular suture (all CS) | Patient satisfaction (10 cm scale): 6 months postoperatively | MD  ‐0.5 [‐1.17, 0.17] (1/95) | Very Low | Mackeen 2012 | IE |
| **Skin closure** | Staples (all CS) | Absorbable subcuticular suture (all CS) | Total operative time (minutes) | MD  ‐5.74 [‐12.49, 1.02] (2/226) | Low | Mackeen 2012 | IE |
| **Skin closure** | Staples (all CS) | Absorbable subcuticular suture (all CS) | Presence of hypertrophic scar at 6 months | 0.99 [0.58, 1.70] (1/95) | Very Low | Mackeen 2012 | IE |
| **Skin closure** | Staples (Pfannestield only) | Absorbable subcuticular suture (Pfannestield only) | Wound infection | 0.41 [0.12, 1.36] (5/500) | Very Low | Mackeen 2012 | IE |
| **Skin closure** | Staples (Pfannestield only) | Absorbable subcuticular suture (Pfannestield only) | Wound complications | 0.44 [0.14, 1.37] (5/500) | Very Low | Mackeen 2012 | IE |
| **Skin closure** | Subcuticular suture | Interrupted suture | Presence of hypertrophic scar at 6 months | 1.85 [1.33, 2.58] (1/65) | Low | Mackeen 2012 | PH |
| **Skin closure** | Barbed suture | PDS suture (subcuticular polydiaxanone suture) (slowly absorbable) | Wound infection | 0.96 [0.18, 5.10] (1/188) | Low | Mackeen 2012 | IE |
| **Skin closure** | Barbed suture | PDS suture (subcuticular polydiaxanone suture) (slowly absorbable) | Wound complication | 1.44 [0.30, 6.93] (1/188) | Low | Mackeen 2012 | IE |
| **Skin closure** | Barbed suture | PDS suture (subcuticular polydiaxanone suture) (slowly absorbable) | Time to skin closure of dermal and epidermal layer (minutes) | MD  0.60 [‐0.30, 1.50] (1/188) | Low | Mackeen 2012 | IE |
| **Skin closure** | Barbed suture | Conventional sutures | Combined postoperative morbidity | 0.88 (0.46, 1.65) (1/188) | Very low | Agarwal 2020 | IE |
| **Skin closure** | Clips | Sutures | Skin closure time (in minutes) | MD  −5.35 [−6.75 to−3.95] (8/1728) | Moderate | Zaman 2021 | CEB |
| **Skin closure** | Clips | Sutures | Wound separation | 2.33 [1.31–4.12] (9/2644) | Moderate | Zaman 2021 | CEH |
| **Skin closure** | Clips | Sutures | Wound infection | 1.12 [0.56–2.25] (9/2605) | Low | Zaman 2021 | IE |
| **Skin closure** | Clips | Sutures | Wound haematoma | 2.46 [0.56–10.75] (4/1364) | Low | Zaman 2021 | IE |
| **Skin closure** | Clips | Sutures | Wound seroma | 1.17 [0.48–2.83] (4/1364) | Low | Zaman 2021 | IE |
| **Skin closure** | Clips | Sutures | Maternal re-admission | 1.28 [0.32–5.02] (3/1462) | Very Low | Zaman 2021 | IE |
| **Skin closure** | Clips | Sutures | Length of hospital stay (in days) | MD  1.21 [0.14–2.29] (5/1636) | Very Low | Zaman 2021 | IE |
| **Skin closure** | Clips | Sutures | Patient scar assessment scale (PSAS) | MD  0.44 [−2.10–2.99] (7/1457) | Low | Zaman 2021 | IE |
| **Skin closure** | Clips | Sutures | Observer scar assessment scale (OSAS) | MD  0.32 [−0.75–1.40] (7/1457) | Low | Zaman 2021 | IE |
| **Skin closure** | Absorbable suture All studies | Nonabsorbable metal staples All studies | Wound Separation | 0.43 [0.32, 0.58 ( 11/2592) | Moderate | Mackeen 2022 | CEB |
| **Skin closure** | Absorbable suture All studies | Nonabsorbable metal staples All studies | Wound Infections | 0.93 (0.47–1.85) (14/3530) | Low | Mackeen 2022 | IE |
| **Skin closure** | Absorbable suture All studies | Nonabsorbable metal staples All studies | Hematoma | 1.52 (0.66–3.50) (7/1402) | Low | Mackeen 2022 | IE |
| **Skin closure** | Absorbable suture All studies | Nonabsorbable metal staples All studies | Seroma | 1.01 (0.44–2.35) (5/1188) | Very Low | Mackeen 2022 | IE |
| **Skin closure** | Absorbable suture All studies | Nonabsorbable metal staples All studies | Readmision for wound concerns | 1.08 (0.49–2.40) (3/1342) | Very Low | Mackeen 2022 | IE |
| **Skin closure** | Monofilament suture (poliglecaprone or polypropylene) | Multifilament suture (polyglactin) | SSI | 0.71 [0.52, 0. 98] (4/1845) | Low | Sobodu 2024 | PB |
| **Skin closure** | Monofilament suture (poliglecaprone or polypropylene) | Multifilament suture (polyglactin) | Hematoma | 0.70 [0.33, 1. 45] (4/1845) | Very Low | Sobodu 2024 | IE |
| **Skin closure** | Monofilament suture (poliglecaprone or polypropylene) | Multifilament suture (polyglactin) | Seroma | 0.79 [0.42, 1.51] (3/1604) | Low | Sobodu 2024 | IE |
| **Skin closure** | Monofilament suture (poliglecaprone or polypropylene) | Multifilament suture (polyglactin) | Wound dehiscence | 0.94 [0.65, 1.37] (3/1641) | Low | Sobodu 2024 | IE |
| **Wound healing** | Negative pressure wound therapy | Standard dressing | Surgical site infection | 0.78 [0.65, 0.95] (9/5529) | High | Norman 2022 | CEB |
| **Wound healing** | Negative pressure wound therapy | Standard dressing | SSI (superficial) | 0.70 [0.53, 0.92] (22/5539) | Moderate | Norman 2022 | CEB |
| **Wound healing** | Negative pressure wound therapy | Standard dressing | Skin blisters | 3.55 [1.43, 8.77] (11/5015) | Low | Norman 2022 | PH |
| **Wound healing** | Negative pressure wound therapy | Standard dressing | SSI (deep) | 0.95 [0.76, 1.18] (22/8521) | Moderate | Norman 2022 | CEND |
| **Wound healing** | Negative pressure wound therapy | Standard dressing | Dehiscence | 1.01 [0.82, 1.24] (6/5113) | High | Norman 2022 | CEND |
| **Wound healing** | Negative pressure wound therapy | Standard dressing | Haematoma | 0.79 [0.48, 1.30] (17/5909) | Low | Norman 2022 | IE |
| **Wound healing** | Negative pressure wound therapy | Standard dressing | Pain | 1.52 [0.20, 11.31] (2/632) | Very low | Norman 2022 | IE |
| **Wound healing** | Negative pressure wound therapy | Standard dressing | Seroma | 0.82 [0.65, 1.05] (15/5436) | Very low | Norman 2022 | IE |
| **Wound healing** | Negative pressure wound therapy | Standard dressing | Reoperation | 1.13 [0.91, 1.41] (18/6272) | Low | Norman 2022 | IE |
| **Wound healing** | Negative pressure wound therapy | Standard dressing | Readmission | 0.98 [0.70, 1.38] (15/5853) | Very low | Norman 2022 | IE |
| **Wound healing** | Negative pressure wound therapy | Standard dressing | Maternal mortality | 0.78 [0.47, 1.30] (11/6384) | Low | Norman 2022 | IE |
| **Wound healing** | Advanced dressing | Simple dressing | SSI | 0.81 (0.52–1.24) (6/2295) | Very low | Wijetunge 2021 | IE |
| **Wound healing** | Advanced dressing | Simple dressing | Endometritis | 1.43 (0.09–23.92) (3/1134) | Very low | Wijetunge 2021 | IE |
| **Wound healing** | Advanced dressing | Simple dressing | Wound dehiscence | 0.51 (0.19,1.34) (4/1496) | Low | Wijetunge 2021 | IE |
| **Wound healing** | Advanced dressing | Simple dressing | Rehospitalisation | 0.70 (0.24, 2.07) (5/ 1638) | Very low | Wijetunge 2021 | IE |
| **Wound healing** | Advanced dressing: Application of silver-impregnated dressings | Simple dressing | SSI and superficial SSI | 1.20 (0.77, 1.88) (2/1132) | Very low | Wijetunge 2021 | IE |
| **Wound healing** | Advanced dressing: DACC-impregnated | Simple dressing | SSI | 0.33 (0.14, 0.77) (2/685) | Low | Wijetunge 2021 | PB |
| **Wound healing** | Advanced dressing: DACC-impregnated | Simple dressing | Wound dehiscence | 0.43 (0.06, 2.88) (4/1496) | Very low | Wijetunge 2021 | IE |
| **Changing gloves** | Changing gloves | No changing gloves | SSI | 0.41 [0.26, 0.65] (4/1036) | High | Narice 2020 | CEB |
| **Changing gloves** | Changing gloves | No changing gloves | Endometritis | 0.96 [0.78, 1.20] (5/1706) | High | Narice 2020 | CEND |
| **Changing gloves** | Changing gloves | No changing gloves | Febrile morbidity | 0.73 [0.30, 1.81) (3/744) | Moderate | Narice 2020 | IE |
| **Changing gloves** | Changing gloves before placenta delivery | No changing gloves | SSI | 0.62 [0.15, 2.49] (2/208) | Very low | Narice 2020 | IE |
| **Changing gloves** | Changing gloves before placenta delivery | No changing gloves | Endometritis | 1 [0.80, 1,26] ( 3/979) | Moderate | Narice 2020 | IE |
| **Changing gloves** | Changing gloves before placenta delivery | No changing gloves | Febrile | 1.30 [0.67, 2.49] (2/208) | Low | Narice 2020 | IE |
| **Changing gloves** | Changing gloves after delivery of the placenta | No changing gloves | SSI | 0.39 [0.24, 0.63] (3/678) | High | Narice 2020 | CEB |
| **Changing gloves** | Changing gloves after delivery of the placenta | No changing gloves | Febrile morbidity | 0.45 [0.19, 1.04] (2/586) | Low | Narice 2020 | IE |
| **Changing gloves** | Changing gloves after delivery of the placenta | No changing gloves | Endometritis | 0.63, [0.30, 1.36] (2/726) | Low | Narice 2020 | IE |
| **Changing gloves** | Changing gloves timing of glove change: After delivery of the foetus | No changing gloves | SSI | 0.63 (0.18, 2.22) (4/408) | Low | Rattanakanokchai 2021 | IE |
| **Changing gloves** | Changing gloves timing of glove change: After delivery of the foetus | No changing gloves | Endometritis | 1.01 (0.81, 1.27) (4/1079) | Moderate | Rattanakanokchai 2021 | IE |
| **Changing gloves** | Changing gloves timing of glove change: After delivery of the foetus | No changing gloves | Febrile morbidity | 1.30 (0.67, 2.49) (2/208) | Low | Rattanakanokchai 2021 | IE |
| **Skin to skin contact** | Immediate skin‐to‐skin versus standard contact for healthy infants | Early skin‐to‐skin versus standard contact for healthy infants | Breastfeeding 1 month to 4 months post birth | 1.22 [1.04, 1.44] (2/220) | Low | Moore 2016 | PB |
| **Skin to skin contact** | Immediate skin‐to‐skin versus standard contact for healthy infants | Early skin‐to‐skin versus standard contact for healthy infants | Exclusive breastfeeding at hospital discharge to 1 month post birth | 1.0 [0.53, 1.88] (1/34) | Low | Moore 2016 | IE |
| **Skin to skin contact** | Immediate skin‐to‐skin versus standard contact for healthy infants | Early skin‐to‐skin versus standard contact for healthy infants | Exclusive breastfeeding 6 weeks to 6 months post birth | 1.16 [0.95, 1.43] (2/144) | Very low | Moore 2016 | IE |
| **Skin to skin contact** | Immediate skin‐to‐skin versus standard contact for healthy infants | Early skin‐to‐skin versus standard contact for healthy infants | Success of the first breastfeeding (IBFAT score) | 1.37 [0.12, 2.62] (2/124) | Very low | Moore 2016 | IE |
| **Skin to skin contact** | Immediate skin‐to‐skin versus standard contact for healthy infants | Early skin‐to‐skin versus standard contact for healthy infants | Maternal state anxiety 8 hours to 3 days post birth | MD  ‐2.70 [‐6.06, 0.66] (1/60) | Very low | Moore 2016 | IE |
| **Skin to skin contact** | Immediate skin‐to‐skin versus standard contact for healthy infants | Early skin‐to‐skin versus standard contact for healthy infants | Respiratory rate 75 minutes ‐ 2 hours post birth | MD  ‐4.48 [‐9.20, 0.24] (1/32) | Low | Moore 2016 | IE |
| **Skin to skin contact** | Immediate skin‐to‐skin versus standard contact for healthy infants | Early skin‐to‐skin versus standard contact for healthy infants | Maternal pain 4 hours post‐caesarean birth | MD  ‐1.38 [‐2.79, 0.03] (1/35) | Low | Moore 2016 | IE |
| **Surgical tapes** | Basic wound contact dressings | Silver dressings | SSI | 0.83 [0.51 to 1.37) (5/1353) | Very Low | Dumville 2016 | IE |

*All effect estimates that are not labelled, are RR.
