# Supplementary material for: Evidence-based surgical procedures to optimize caesarean outcomes: an overview of systematic reviews
Source: eClinicalMedicine. 2024 May 19;72:102632. doi: 10.1016/j.eclinm.2024.102632 (PMC11134562; doi:10.1016/j.eclinm.2024.102632)
Supplement: Supplementary material 6 [file mmc5.docx]

**Resumen (Español)**

**Antecedentes:** La cesárea es la cirugía mayor más practicada en todo el mundo. Las técnicas quirúrgicas utilizadas para la cesárea varían ampliamente, y no existe una estandarización aceptada internacionalmente. Se realizó un estudio de revisiones sistemáticas de ensayos controlados aleatorizados, para resumir las pruebas sobre las técnicas o procedimientos quirúrgicos relacionados con la cesárea.

**Métodos:** Se realizaron búsquedas en Cochrane Database of Systematic Reviews, PubMed, EMBASE, Lilacs y CINAHL, sin restricciones de fecha o idioma. Se utilizaron AMSTAR 2 y GRADE para evaluar la calidad metodológica de las revisiones sistemáticas y la certeza de la evidencia a nivel de resultado, respectivamente. Se usó el binomio *procedimiento-resultado* como unidad de análisis, y cada binomio se clasificó en una de ocho categorías según las estimaciones del efecto y la certeza de la evidencia. El protocolo de este estudio se registró en PROSPERO (CRD 42023208306).

**Resultados:** Se incluyeron 38 revisiones sistemáticas (16 Cochrane y 22 que no eran Cochrane) publicadas entre 2004 y 2024, que incluían 628 ensayos controlados aleatorizados con un total de 190.349 participantes. La mayoría de las revisiones fueron de calidad baja o críticamente baja (AMSTAR 2). Las RS presentaron 345 comparaciones procedimiento-resultado (237 procedimiento frente a procedimiento, 108 procedimiento frente a no tratamiento/placebo). Hubo pruebas insuficientes o no concluyentes para 256 comparaciones, pruebas claras de beneficio para 40 comparaciones, posible beneficio para 17, no diferencias en efecto para 14, pruebas claras de daño para 13 y posible daño para 5. Para 7 procedimientos no se encontraron revisiones sistemáticas. La limpieza de la piel con clorhexidina, la incisión abdominal basada en Joel-Cohen, la incisión uterina con disección roma y expansión céfalo-caudal, la tracción del cordón para la extracción de la placenta, la dilatación cervical manual en las cesáreas anteriores al inicio del parto, el cambio de guantes, la sutura de catgut crómico para el cierre uterino, el no cierre del peritoneo, el cierre del tejido subcutáneo y la terapia de heridas con presión negativa son procedimientos asociados con beneficios para resultados relevantes.

**Interpretación:** La evidencia sugiere que varios procedimientos quirúrgicos utilizados durante una cesárea mejoran los resultados, pero también revela una falta de evidencia o evidencia no concluyente para muchos procedimientos comúnmente utilizados. Se necesitan urgentemente directrices basadas en la evidencia que estandaricen las técnicas para una cesárea segura. También son necesarios ensayos controlados aleatorizados que cubran las lagunas de conocimiento existentes.

**Resumo (Português)**

**Introdução:** A cesariana (CS) é a cirurgia de grande porte mais frequente no mundo. As técnicas cirúrgicas usadas para realizar uma CS variam muito e não existe uma padronização internacional sobre isso. Realizamos um overview de revisões sistemáticas (RS) de ensaios clínicos randomizados (ECR) para sintetizar a evidência existente sobre técnicas ou procedimentos cirúrgicos relacionados à CS.

**Método:** Fizemos buscas nas bases de dados Cochrane Database of Systematic Reviews, PubMed, EMBASE, Lilacs e CINAHL sem limites de data ou idioma. Usamos o AMSTAR2 e GRADE para avaliar a qualidade metodológica das RS e a certeza da evidência dos desfechos, respectivamente. Classificamos cada par de procedimento-desfecho em uma dentre oito possíveis categorias conforme a estimativa de efeito e a certeza da evidência. O protocolo do overview foi registrado no PROSPERO (CRD 42023208306).

**Resultados:**  A análise incluiu 38 RS (16 Cochrane e 22 não-Cochrane) publicadas em 2004-2024 envolvendo 628 ECR com um total de 190.349 participantes. A qualidade metodológica da maioria das revisões foi baixa ou criticamente baixa (AMSTAR2). As RS apresentavam 345 comparações procedimento-desfescho (237 comparavam dois procedimentos diferentes e 108 comparavam um procedimento com nenhum tratamento ou um placebo). Encontramos evidência insuficiente ou inconclusiva para 256 comparações, clara evidência de benefício para 40, possível benefício para 17, ausência de diferença de efeito para 14, clara evidência de danos para 13 e possível dano para 5. Para 7 comparações pré-definidas, não encontramos nenhuma RS. A limpeza da pele com clorexidina, incisão abdominal usando a técnica de Joel-Cohen, incisão uterina com dissecção romba e expansão cefalocaudal, extração placentária com tração do cordão, dilatação cervical manual na CS fora do trabalho de parto, troca de luvas, sutura uterina com categute cromado, não fechamento do peritônio, sutura do tecido celular subcutâneo e terapia de pressão negativa para cicatrização da ferida operatória são procedimentos com benefícios para desfechos relevantes.

**Interpretação:** A evidência atualmente disponível indica que vários procedimentos cirúrgicos usados em CS estão associados a melhores desfechos. Porém, também existe ausência de evidência ou evidência insuficiente para muitos procedimentos comumente usados em CS. Existe uma necessidade urgente de criar diretrizes baseadas em evidência para padronizar as técnicas cirúrgicas usadas na CS e realizar mais ensaios clínicos para preencher as brechas de conhecimento existentes.

**Financiamento**: UNDP-UNFPA-UNICEF-WHO-World Bank Special Programme of Research, Development and Research Training in Human Reproduction (HRP), um programa copatrocinado executado pela Organização Mundial da Saúde (OMS).

**Résumé (Français)**

**Contexte :** La césarienne est l'intervention chirurgicale la plus pratiquée dans le monde. Les techniques chirurgicales utilisées pour la césarienne varient considérablement et il n'existe aucune pratique standardisées reconnue au niveau international. Nous avons réalisé une étude de revues systématiques des essais contrôlés randomisés afin de résumer les résultats obtenus sur les techniques ou procédures chirurgicales liées à la césarienne.

**Méthodes :** Des recherches ont été effectuées dans la base de données Cochrane Database of Systematic Reviews, PubMed, EMBASE, Lilacs et CINAHL sans restriction de date ou de langue. AMSTAR 2 et GRADE ont été utilisés pour évaluer la qualité méthodologique des revues systématiques et la qualité globale des preuves pour les résultats obtenus. Nous avons classé chaque paire *procédure-résultat* dans huit catégories en fonction des estimations de l'effet de la procédure et de la confiance que l’on peut avoir dans l’effet observé. Le protocole de l’étude a été enregistrée sur PROSPERO (CRD 42023208306).

**Résultats :** Au final 38 revues systématiques ont été inclues (16 revues Cochrane et 22 revues non-Cochrane) publiées entre 2004 et 2024, regroupant 628 essais contrôlés randomisés avec un total de 190 349 participants. La plupart des revues étaient de faible voir de très faible qualité - (AMSTAR 2). On présente 345 comparaisons procédure-résultat (237 procédure vs procédure, 108 procédure vs pas de traitement/placebo). Le niveau de preuve était fort de bénéfice pour seulement 40 comparaisons procédure-résultat paires, possible de bénéfice pour 17, pas de différence d'effet pour 14, des preuves fort de préjudice pour 13, un préjudice possible pour 5 et des preuves insuffisantes ou non concluantes pour 256. Nous n'avons pas trouvé de revues systématiques pour 7 procédures prédéfinies. Le nettoyage de la peau à la chlorhexidine, l'incision abdominale basée sur la méthode de Joel-Cohen, l'incision utérine avec divulsion digitale et expansion cranio-caudale, la traction du cordon pour la délivrance du placenta, la dilatation cervicale manuelle en cas de CS avant travail, le changement de gants, hystérorraphie avec catgut chromé, la non-fermeture du péritoine, la fermeture du tissu sous-cutané et la cicatrisation dirigée par pression négative sont des procédures associées à un bénéfice de l’intervention.

**Interprétation :** Les données actuelles suggèrent que plusieurs procédures chirurgicales de la césarienne améliorent les résultats, mais révèlent également un manque de preuves suffisant pour de nombreuses procédures couramment utilisées. Il y a un besoin urgent de recommandations internationales sur les techniques de la césarienne basées sur des preuves, ainsi que des essais pour combler les lacunes dans les connaissances.
