# Supplementary material for: Evidence-based surgical procedures to optimize caesarean outcomes: an overview of systematic reviews
Source: eClinicalMedicine. 2024 May 19;72:102632. doi: 10.1016/j.eclinm.2024.102632 (PMC11134562; doi:10.1016/j.eclinm.2024.102632)
Supplement: Supplementary Material 7 [file mmc6.docx]

| **Procedure-outcome comparisons** | | | |
| --- | --- | --- | --- |
|  | **procedure-procedure** | **procedure-NT*/placebo** | Total |
| clear evidence of benefit (CEB) | 27 | 13 | 40 |
| possible benefit  (PB) | 10 | 7 | 17 |
| clear evidence no difference  (CEND) | 8 | 4 | 12 |
| possible no difference (PEND) | 1 | 0 | 1 |
| clear evidence of harm (CEH) | 10 | 4 | 14 |
| possible harm  (PH) | 4 | 1 | 5 |
| insufficient evidence  (IE) | 177 | 79 | 256 |
| Total | 237 | 108 | 345 |

*N/T: no treatment
